# Understanding coenzyme Q

**DOI:** 10.1152/physrev.00040.2023

**Published:** 2024-05-09

**Authors:** Ying Wang, Noah Lilienfeldt, Siegfried Hekimi

**Affiliations:** Department of Biology, McGill University, Montreal, Quebec, Canada

**Keywords:** coenzyme Q, CoQ, CoQ deficiency, mitochondrial disease, ubiquinone

## Abstract

Coenzyme Q (CoQ), also known as ubiquinone, comprises a benzoquinone head group and a long isoprenoid side chain. It is thus extremely hydrophobic and resides in membranes. It is best known for its complex function as an electron transporter in the mitochondrial electron transport chain (ETC) but is also required for several other crucial cellular processes. In fact, CoQ appears to be central to the entire redox balance of the cell. Remarkably, its structure and therefore its properties have not changed from bacteria to vertebrates. In metazoans, it is synthesized in all cells and is found in most, and maybe all, biological membranes. CoQ is also known as a nutritional supplement, mostly because of its involvement with antioxidant defenses. However, whether there is any health benefit from oral consumption of CoQ is not well established. Here we review the function of CoQ as a redox-active molecule in the ETC and other enzymatic systems, its role as a prooxidant in reactive oxygen species generation, and its separate involvement in antioxidant mechanisms. We also review CoQ biosynthesis, which is particularly complex because of its extreme hydrophobicity, as well as the biological consequences of primary and secondary CoQ deficiency, including in human patients. Primary CoQ deficiency is a rare inborn condition due to mutation in CoQ biosynthetic genes. Secondary CoQ deficiency is much more common, as it accompanies a variety of pathological conditions, including mitochondrial disorders as well as aging. In this context, we discuss the importance, but also the great difficulty, of alleviating CoQ deficiency by CoQ supplementation.

## Graphical abstract

**Figure:**
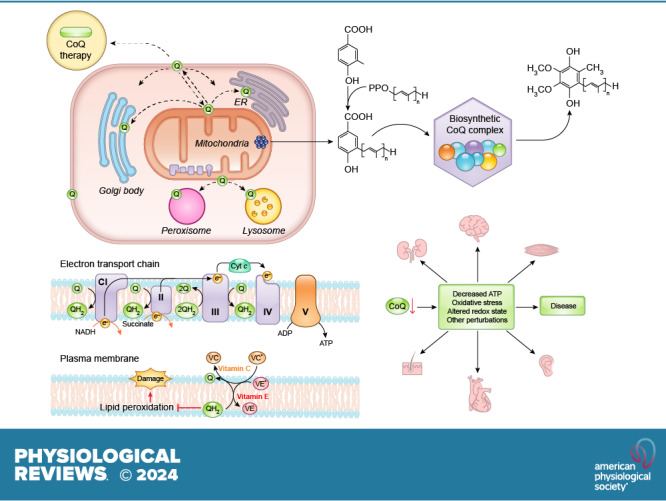

CLINICAL HIGHLIGHTS Coenzyme Q_10_ (CoQ_10_) was discovered more than half a century ago for its key role in mitochondrial respiration. It also participates in several other important cellular functions such as reactive oxygen species (ROS) generation during mitochondrial respiration, protection against oxidation of membrane lipids, and the redox balance of the cell. Mutations in the genes required for the biosynthesis of CoQ_10_ lead to primary CoQ_10_ deficiency (PCD) and present with heterogeneous clinical symptoms ranging from birth- or infantile-onset multisystem disorders to isolated symptoms involving single organs or systems. Overall, PCD frequently resembles mitochondrial disease syndromes. However, it is unknown whether the same pathophysiology underlies each symptom. For example, some studies in mice suggest that it is increased oxidative stress due to low CoQ, and not damaged mitochondrial function, that is responsible for renal symptoms. In addition to PCD, a variety of diseases and conditions have been found to be associated with secondary CoQ_10_ deficiency (SCD), which refers to all the conditions in which the etiology of the CoQ_10_ deficiency is not a molecular lesion in the CoQ_10_ biosynthetic pathway. These include mitochondrial disorders, multiple system atrophy, ataxia due to *APTX* mutations, mutations in *ETFDH*, and Parkinson’s disease. In addition to patients with documented CoQ_10_ deficiency and/or mutations of biosynthetic genes, CoQ_10_ is frequently recommended to mitochondrial disease patients as well as for treating a wide range of other conditions (e.g., heart failure and neurodegenerative diseases). Oral CoQ_10_ supplementation is the only currently available treatment option for CoQ_10_ deficiency. However, a recent systematic review of all PCD patients who have been treated with CoQ_10_ suggests that oral supplementation is virtually without effect, despite the fact that the lack of CoQ_10_ is the primary cause of these patients’ symptoms. Future research will be necessary to develop effective therapies to treat or prevent CoQ_10_ deficiency.

## 1. INTRODUCTION

Coenzyme Q (CoQ), also known as ubiquinone (UQ), is a lipophilic molecule that is essential for several distinct cellular processes, including energy production, and is thus essential for life. It is one of the most conserved molecules across all kingdoms of life. Frederick Crane and colleagues (1) at the Enzyme Institute of the University of Wisconsin in Madison first isolated it in 1957 from beef heart mitochondria as a yellow-orange lipophilic substance with redox properties, and it was proposed to function as a coenzyme for mitochondrial electron transfer. As such, it was given the name coenzyme Q. Its other name, ubiquinone, which was officially given to the substance in 1975 by the IUPAC-IUB Commission on Biochemical Nomenclature, refers to the fact that it has a ubiquitous presence from bacteria to humans.

**FIGURE 1.**
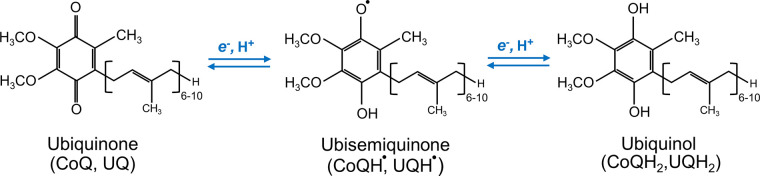
Structure and redox states of coenzyme Q (CoQ). CoQ exists in 3 redox states: the fully oxidized form (CoQ) accepts 2 electrons to form CoQH_2_ or accepts 1 electron to form the ubisemiquinone intermediate, followed by acceptance of an additional electron to form CoQH_2_. The number of isoprene units in the tail varies between species from 6 to 10.

The chemical structure of CoQ was determined by Karl Folkers and coworkers at Merck. Its full chemical name is often given as 2,3-dimethoxy-5-methyl-6-multiprenyl-1,4-benzoquinone. Another possible formalism is 2-methyl-3-multiprenyl-5,6-dimethoxy-1,4-benzoquinone, which we are following in this review, including in the figures. CoQ is composed of a redox-active benzoquinone ring conjugated to an isoprenoid unbranched side chain whose length is species specific, ranging from 6 to 10 isoprenoid repeats (FIGURE 1). For example, in humans, the side chain is 10 isoprene subunits long and the molecule is therefore abbreviated as CoQ_10_. Rodents and *Caenorhabditis elegans* (*C. elegans*) mainly produce CoQ_9_, and *Saccharomyces cerevisiae* (*S. cerevisiae*) and *Escherichia coli* (*E. coli*) produce CoQ_6_ and CoQ_8_, respectively. Some species make more than one form of CoQ. For example, although CoQ_9_ is the main form in mice, small amounts of CoQ_10_ also occur in most tissues, with tissue-specific ratios of the two forms. Why different organisms have CoQ with varying side chain lengths is not understood. The CoQ benzoquinone ring is the functional group of the molecule, capable of reversible oxidation-reduction states without change in structure. The benzoquinone ring of quinone can exist in nine different redox states (2, 3). However, functionally there are three redox states of CoQ, that is, fully oxidized (CoQ, UQ), partially reduced (a semiquinone anion radical with a reactive unpaired electron, CoQ^•^**^−^**, UQ^•^**^−^**), and fully reduced (CoQH_2_, UQH_2_) (4, 5) (FIGURE 1). The redox chemistry of CoQ, which is able to accept/donate one or two electrons at a time, is at the core of its best-understood biological functions (6). The isoprenoid tail is responsible for the extreme hydrophobicity of CoQ and its solubility in membrane bilayers (6).

CoQ is likely found in all eukaryotic lipid membranes (7, 8). The best-known function of CoQ is to act as an electron carrier in the electron transport chain (ETC) in the inner membrane of mitochondria (IMM). In fact, mitochondria are the most enriched in CoQ among all subcellular compartments (9, 10). Other functions described for CoQ include participation in trans-plasma membrane electron transport, regulation of the mitochondrial permeability transition pore (mPTP), and activation of uncoupling proteins (UCPs), as well as an important dual role as pro- and antioxidant (7, 11–13). It has been proposed that CoQ may also play a role in the physicochemical properties of the lipid membranes in which it resides, but this is not yet well established or understood (14–23). In addition, new aspects of the function of CoQ are regularly reported. For example, a recent study suggests that the ratio of reduced to oxidized CoQ (CoQ/CoQH_2_) helps metabolic adaptation by acting as a sensor of the efficiency of the mitochondrial ETC (24). CoQ deficiency, no matter its cause, is currently defined as a decrease in the CoQ content in cells or organisms that can potentially impair many cellular functions, with mitochondria respiration expected to be the most vulnerable.

All cells rely on endogenous synthesis for their CoQ supply. CoQ biosynthesis is a complex and highly conserved pathway in which at least 10 proteins are involved. In eukaryotes, CoQ is synthesized from precursors in the IMM, from which it is then distributed to other subcellular compartments (25–27). To date, it is well established that several CoQ biosynthetic pathway components are recruited into a supramolecular complex that catalyzes sequential reactions that modify the aromatic ring (25–28). In animals, complete loss of CoQ biosynthesis is embryonic lethal in most species (8, 29–32). However, see the description in sect. 5.2 of the special case of *clk-1* mutants of the nematode *C. elegans*, which can survive with a mixture of dietary CoQ and the CoQ biosynthetic intermediate demethoxyubiquinone (DMQ) (33–36). In humans, deleterious mutations in genes required for CoQ biosynthesis frequently cause severe multisystem disease due to impaired mitochondrial respiration (11, 26, 37–39). After diagnosis, the patients are generally treated with oral CoQ_10_ supplementation. Unfortunately, there is only very weak evidence for the efficacy of the treatment (40). Efforts are underway to develop methods for more effective CoQ_10_ delivery to overcome its extremely poor water solubility and limited oral bioavailability (41, 42). Moreover, in view of its essential role in mitochondrial respiration and its antioxidant capabilities, CoQ_10_ has been recommended to treat conditions with no evidence of CoQ_10_ deficiency as a causative factor, such as congestive heart failure, neurodegenerative diseases, cancer, and more (43). However, in our view, whether CoQ_10_ supplementation truly provides benefits to any type of patient remains in need of a clear demonstration.

## 2. THE FUNCTION OF CoQ AS MITOCHONDRIAL ELECTRON TRANSPORTER

### 2.1. Requirement of CoQ in Aerobic Respiration

After its discovery, the most crucial function that CoQ has been shown to perform is as an electron carrier in the mitochondrial ETC (44–46). This key function of CoQ became evident in the late 1960s when it was demonstrated that depletion of CoQ_10_ from beef heart submitochondrial particles (SMPs) by pentane extraction caused inhibition of both the NADH and succinate oxidase activities and that the activities were restored upon reconstitution of extracted SMPs with CoQ at physiological concentrations (47, 48).

**FIGURE 2.**
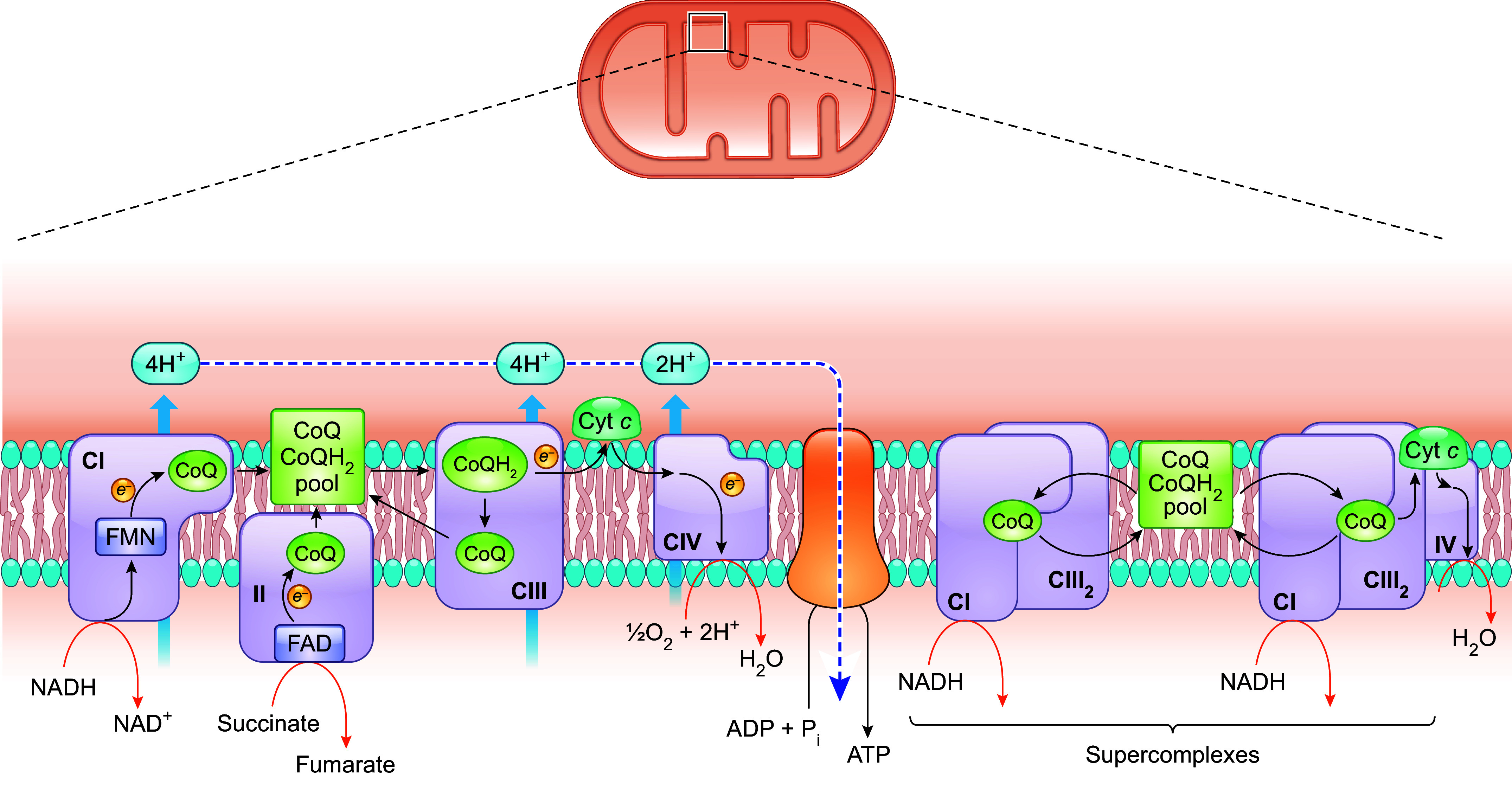
Functions of CoQ in the mitochondrial respiratory chain. CoQ is a pivotal component of the mitochondrial electron transport chain, acting as a mobile electron carrier shuttling electrons from CI and CII to CIII. During this process, CoQ cycles between reduced and oxidized states. In addition to moving randomly and colliding with CI and CII, CoQ is also present in CI- and CIII-containing respiratory supercomplexes (SCs), formed by the dynamic association of ETC complexes. In SCs, CIII is normally observed as a dimer (CIII_2_). All CoQ in the IMM likely behaves as a single functional pool, that is, CoQH2 can diffuse out of the CI and CIII assembled in SCs and become oxidized by CIII found outside of SCs. Conversely, CoQH_2_ generated independently of SCs can diffuse in, and be oxidized by, CIII attached to CI assembled in SCs. See glossary for other abbreviations.

Mitochondrial complex I (CI) is the nicotinamide adenine dinucleotide (NADH)-CoQ oxidoreductase. Reduction of CoQ by electrons from CI is the last step of the electron transfers between sites across CI, which, as a whole, powers proton (H^+^) translocation across the IMM into the intermembrane space (FIGURE 2). CoQ also accepts electrons from complex II (CII is the succinate CoQ reductase), a process that does not translocate protons across the inner membrane directly but participates in respiratory chain function by increasing the size of the pool of reduced CoQ (CoQH_2_). CoQH_2_ resulting from electron transfer from CI and CII and other metabolic enzymes enters complex III (CIII is the cytochrome *bc*_1_ complex), where it transfers electrons to cytochrome *c* (cyt *c*) and thus becomes reoxidized (FIGURE 2). In CIII, CoQ undergoes the Q cycle (see below), whose net result is the translocation of 4 protons across the IMM for the full oxidation of each CoQH_2_ molecule (46). This ends the role of CoQ in electron transport and in creating the mitochondrial transmembrane potential and proton gradient (49).

It has been suggested that only 10–32% of total mitochondrial CoQ is bound to membrane proteins (50, 51). The classic liquid-state or random-collision model postulates that in the IMM there exists a bulk CoQ pool that is accessible to all the dehydrogenases that need to donate reducing equivalents to CoQ. In this model, CoQ diffuses freely within the lipid bilayer and electron transfer occurs after random collisions between CoQ and the enzymes that are themselves diffusing in the plane of the IMM, including the ETC complexes (52). This view, however, has been partially abandoned, after the discovery that individual ETC complexes can assemble into a variety of supramolecular structures known as supercomplexes (SCs) (53–57). The major SCs identified comprise CI/CIII_2_ (CI associated with a CIII dimer), CI/CIII_2_/CIV, and CIII_2_/CIV. The SC formed by CI, CIII_2_, and CIV is also known as the respirasome because, in principle, it has all the elements required to carry out respiration (53). There is evidence that molecules of CoQ are present in SC assemblies, more specifically in the lipid boundary between CI and CIII, and that the electron transfer between the two complexes occurs through CoQ trapped within (53, 58, 59). In fact, purified SCs (CI/CIII_2_ and CI/CIII_2_/CIV) were shown to be functional, being able to transfer electrons without the addition of any external CoQ (60).

One early hypothesis was that the purpose of SCs might be to mediate substrate channeling to enhance metabolic efficiency. This would mean that each SC sequesters its own subpopulation of the mobile electron carriers (CoQ and/or cytochrome *c*) and electron transfer between two sequential enzymes (CI-CIII and CIII-CIV) occurs by successive reduction and reoxidation of the mobile intermediates that are enclosed in internal channels connecting one enzyme active site to another (50, 61). Such channels would prevent the reaction intermediates from diffusing into the bulk membrane pool, thus minimizing the distance that the intermediates must travel between active sites, with an overall effect of increasing electron transport efficiency (50, 60). However, no robust evidence has yet been found that indicates the presence in SCs of confined spaces that connect active sites and retain mobile redox cofactors within (57, 62, 63). Rather, enzyme kinetic analyses and experiments where the addition of an alternative CoQH_2_ oxidase (AOX) to bovine heart mitochondrial membranes caused a substantial increase of the electron flux through the CI/CIII_2_/CIV suggest that there is no sealed-in CoQ pool in SCs (64, 65). AOX from plants directly oxidizes CoQH_2_, using oxygen (O_2_) as the terminal electron acceptor. The fact that it can compete with the CIII/CIV pathway for electrons indicates that it has access to the CoQH_2_ pool. In mammalian mitochondria, CI is shown to be mostly associated with other complexes in SCs (54). Therefore, if there is direct substrate channeling of CoQ in SCs, the presence of AOX outside of the SC structure should have a negligible effect on electron flux from CI to oxygen. The fact that the addition of AOX was found to increase the NADH oxidation rate suggests that CoQH_2_ can diffuse out of SCs to react with AOX (65). Furthermore, functional and structural characterization of mammalian SCs (from ovine heart mitochondria) demonstrated the existence of CoQ in three of four possible CoQ-binding sites in CI/CIII_2_ SCs: the two Q_i_ sites and one of the two Q_o_ sites of the two CIII. The study also showed that CoQ trapping in the SC actually reduces CI activity, which is also inconsistent with the substrate channeling hypothesis (59).

Even without direct channeling within SCs, the assembly of SCs could still decrease the traveling distance for the electron carriers and thus facilitate more efficient electron transfer (59, 66). As discussed in sect. 3.1, the ETC is the major site of ROS production in the cell. During respiration, electrons can escape from the ETC and be captured by molecular oxygen, thus generating superoxide (O_2_^•−^). CoQ in the ubisemiquinone state is known to be one of the sources of electron leakage. Overall, although the exact nature and role of SC formation are not yet clear, an often-accepted view is that it is beneficial. By facilitating electron transfer, it potentially increases respiration rate and lowers electron leakage to molecular oxygen, thus boosting OXPHOS efficiency and minimizing ROS generation (67, 68). Furthermore, a role in supporting the structural stability of the individual complexes has been proposed (56). Supporting evidence shows that respiratory activity is enhanced when more SCs are formed and an organism’s fitness is compromised when SCs formation is impaired (69–71).

As mentioned above, in mammalian mitochondria, it is believed that all or most of CI (≥90%) is associated with other complexes in SCs (54). Whether CII participates in any SC formation is still an open question (72). It has been proposed that, given the likelihood of free CoQ diffusion in and out of SCs, the overall electron flux through the ETC occurs by a mixture of electron transfer in SCs and random collision events between the two mobile electron carriers (CoQ and cyt *c*) and individual ETC complexes. This is consistent with data that show that all CoQ in the IMM (whether or not associated with SCs) behaves as a single functional pool (57, 59, 73). In other words, CI, CII, and other enzymes that deliver electrons to CoQ (see sect. 2.2) compete for the same CoQ pool (64, 74). However, it is worth noting that this is still a matter of controversy (50, 56, 65). Furthermore, as discussed further in sect. 2.3, CoQ deficiency is usually found to be associated with a partial loss of both CI- and CII-mediated respiration, which is not in support of the existence of two segregated CoQ pools.

### 2.2. Other Electron Transport Pathways That Deliver Electrons to Mitochondrial CoQ

In addition to the electrons that the two respiratory complexes CI and CII transfer to CoQ, it also receives electrons from at least seven other dehydrogenases that are associated with the IMM, either on the intermembrane space side or on the matrix side. These include *1*) the mitochondrial glycerol 3-phosphate dehydrogenase (G3PDH), a part of the glycerophosphate shuttle (75), *2*) the mitochondrial dihydroorotate dehydrogenase (DHODH), an enzyme involved in a key step in the production of pyrimidine nucleotides (76), *3*) the electron transport flavoprotein dehydrogenase (ETFDH), a key enzyme of fatty acid β-oxidation and amino acid catabolism, *4*) proline dehydrogenase (PRODH) and proline dehydrogenase 2 (PRODH2), both of which are involved in proline, glyoxylate, and arginine metabolism, *5*) choline dehydrogenase (CHDH), which is primarily found in liver and kidney in humans and catalyzes the oxidation of choline to glycine betaine (77), and *6*) sulfide-quinone oxidoreductase (SQOR), which is essential for detoxification of hydrogen sulfide (H_2_S) (78). Like CII, these dehydrogenases reduce flavin adenine dinucleotide (FAD) to FADH_2_, which then transfers electrons to CoQ, but these processes are not coupled to proton translocation to the mitochondrial intermembrane space (IMS) because FADH_2_ and CoQ have similar reduction potentials and therefore these transfers do not result in a sufficiently large gain in Gibbs free energy to power proton translocation. To date, there is not much known about how tightly the rates at which these metabolic pathways function are linked to the level of CoQ in the IMM or whether an impact on these pathways contributes to the pathophysiology of CoQ deficiency. Below we briefly describe two of the enzymes whose activities have been reported to be affected by CoQ deficiency.

**FIGURE 3.**
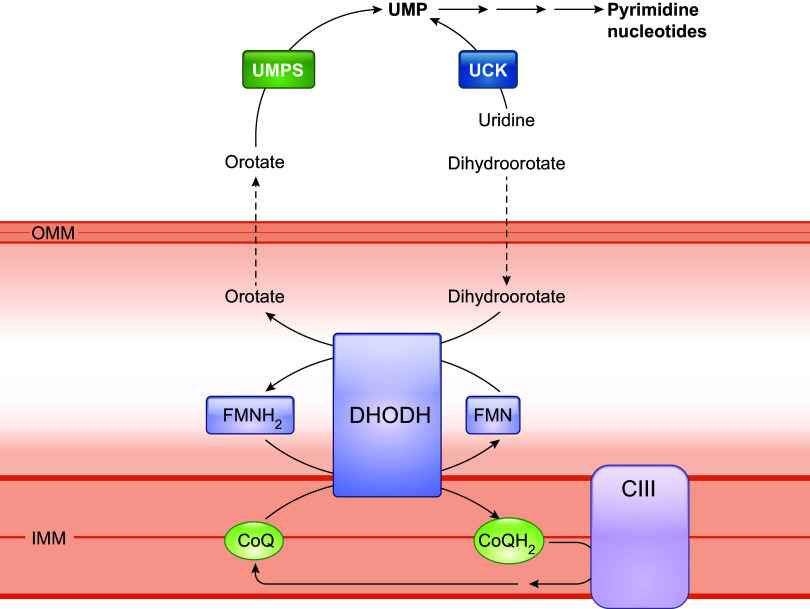
CoQ is a cofactor for mitochondrial dihydroorotate dehydrogenase (DHODH). DHODH catalyzes the oxidation of dihydroorotate to orotate during the fourth step of the de novo biosynthesis of pyrimidine. The reaction is coupled to the reduction/oxidation of flavin mononucleotide (FMN) and CoQ. Orotate diffuses back to the cytosol to be converted by uridine monophosphate synthase (UPMS) to uridine 5*-*monophosphate (UMP), the precursor of all pyrimidine nucleotides. Preexisting uridine can be phosphorylated to UMP by uridine kinase (UCK), thereby bypassing the need for the DHODH-catalyzed step. See glossary for other abbreviations.

Eukaryotic cells devoid of mitochondrial DNA (ρ^0^) need supplementation with uridine to sustain viability. This is because DHODH, which catalyzes a crucial step in intracellular de novo pyrimidine biosynthesis (conversion of dihydroorotate to orotate), needs CoQ as a cofactor (FIGURE 3). The activity of DHODH is inhibited in ρ^0^ cells because of the loss of the ETC and the resultant lack of oxidized CoQ to which electrons can be transferred. Uridine is a downstream product of DHODH and therefore needs to be provided to ρ^0^ cells to compensate for the lack of endogenous pyrimidine biosynthesis, which is essential for RNA/DNA synthesis (76, 79). Uridine was reported to improve the growth rate of human *COQ2* mutant fibroblasts that have <20% residual CoQ_10_, suggesting the possibility of a deficit of pyrimidine biosynthesis in these cells (80, 81). In contrast, no exogenous addition of uridine to the culture medium was needed for *Pdss2/Coq7* double-knockout mouse embryonic fibroblasts (MEFs), despite being completely devoid of detectable CoQ, and in fact these cells showed no sign of any growth defect under standard culture conditions in medium that contained sufficient glucose (82, 83). Normal culture medium contains a minimal amount of CoQ_10_. Thus, in contrast to ρ^0^ cells, *Pdss2/Coq7* double-knockout cells sustain some ETC activity at an extremely low level despite a complete lack of CoQ biosynthesis (83). This low level of CoQ and respiratory function appears to allow for adequate pyrimidine synthesis, suggesting a very minimal requirement for mitochondrial respiratory function to maintain sufficient DHODH activity for cells to survive.

**FIGURE 4.**
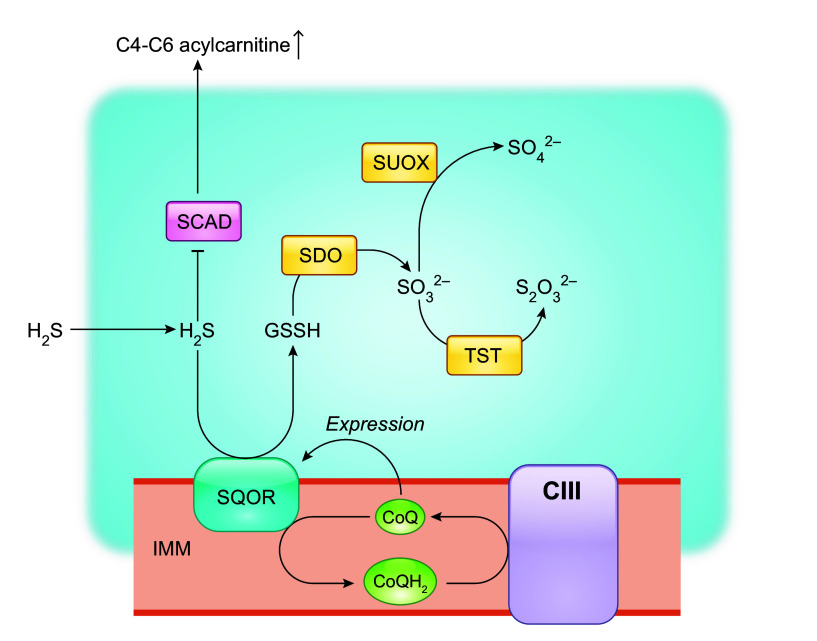
CoQ levels modulate sulfide-quinone oxidoreductase (SQOR) activity. SQOR catalyzes the initial oxidation of hydrogen sulfide (H_2_S) and utilizes CoQ as the electron acceptor. Sulfur is primarily transferred to glutathione (GSH) under physiological conditions, forming glutathione persulfide (GSSH), which is then oxidized by sulfur dioxygenase (SDO), to produce sulfite ($SO_{3}^{2-}$) and regenerate GSH. SDO is also known as ethylmalonic encephalopathy protein1 (ETHE1), as its mutations are associated with ethylmalonic encephalopathy, an infantile metabolic disorder. GSSH is also a substrate for thiosulfate sulfurtransferase (TST), which converts $SO_{3}^{2-}$ to thiosulfate ($S_{2}O_{3}^{2-}$). Alternatively, $SO_{3}^{2-}$ is converted to sulfate ($SO_{4}^{2-}$) by sulfite oxidase (SUOX) residing in the intermembrane space. A further effect of H_2_S accumulation is inhibition of the enzymatic activity of short-chain acyl-CoA dehydrogenase (SCAD) that catalyzes the first reaction in the β-oxidation of short-chain fatty acids. Elevated blood butyrylcarnitine (C4) is the hallmark biomarker of SCAD deficiency. In addition to acting as a cofactor of SQOR, CoQ levels regulate SQOR transcriptionally by an unknown mechanism. See glossary for other abbreviations.

The IMM flavoprotein protein sulfide-quinone oxidoreductase (SQOR) is the first enzyme to act in the mitochondrial metabolism of hydrogen sulfide (H_2_S). It catalyzes two-electron oxidation of H_2_S and utilizes CoQ as the electron acceptor, thus coupling the reaction to CoQ in the ETC (84–86) (FIGURE 4). The oxidized sulfur is transferred to a small-molecule acceptor, which is predicted to be primarily glutathione (GSH) under physiological conditions (84). Glutathione persulfide (GSSH) produced by SQOR is converted to sulfite ($SO_{3}^{2-}$) which is further catabolized by thiosulfate sulfurtransferase (TST, also known as rhodanese) or sulfite oxidase (SUOX) to produce thiosulfate ($S_{2}O_{3}^{2-}$) or sulfate ($SO_{4}^{2-}$) (84). This sulfide oxidation pathway plays a key role in governing cellular H_2_S levels (85). H_2_S has toxic properties but also functions in regulating homeostasis as a cell signaling molecule (78). In human skin fibroblasts, a ≤50% reduction in CoQ_10_ levels was shown to cause an impairment of SQOR-driven oxygen consumption (87). Moreover, accumulation of H_2_S, a direct consequence of impaired sulfide oxidation, was reported for CoQ-deficient fission yeast and mouse tissues (87–89). Other abnormalities related to H_2_S accumulation include depletion of GSH, reduction of thiosulfate ($S_{2}O_{3}^{2-}$), increased protein sulfhydration, and increased blood levels of C4-C6 acylcarnitines, consistent with inhibition of short-chain acyl-CoA dehydrogenase (SCAD), a known toxic effect of H_2_S (87, 89–91). Interestingly, among the mouse tissues examined, including the kidney, brain, and muscle, the kidney showed the most pronounced accumulation of H_2_S (87, 89). High levels of sulfide were observed in the kidney of two different CoQ_9_-deficient mouse models (*Pdss2^kd/kd^* and *Coq9^R239X^*) which have <15% residual CoQ_9_ levels, whereas in the cerebrum of *Coq9^R239X^* mice (with 10–15% residual CoQ_9_) and the whole brain of *Pdss2^kd/kd^* mice (with ≈30% residual CoQ_9_), the levels of sulfides were shown to be similar to those in wild-type control mice (87, 89). Somewhat surprisingly, CoQ deficiency decreases SQOR levels, worsening the effect on sulfide metabolism (87, 89, 90). Conversely, supraphysiological levels of CoQ_10_ (>2,300 fold!) were shown to upregulate SQOR expression in cultured skin fibroblasts, and a similar effect was observed in the liver of wild-type mice after supplementation with CoQ_10_H_2_ (92). Moreover, the amount of reduced SQOR in mutant HeLa cells with ≈50% residual CoQ_10_ was shown to be elevated after CoQ_10_ supplementation (90). Long-term CoQ_10_ treatment was shown to partially rescue decreased SQOR protein levels in the kidney of *Pdss2^kd/kd^* mutant mice despite only a small rise in CoQ_10_ levels, suggesting a high sensitivity of SQOR levels to CoQ levels (90). The mechanisms underlying the connection between the levels of CoQ and SQOR expression are not understood. It also remains to be elucidated how altered sulfide metabolism participates in the development and progression of kidney disease due to CoQ deficiency and what possible significance the CoQ-SQOR connection could have for CoQ_10_ supplementation therapy.

### 2.3. CoQ Concentration and Respiratory Capacity

Most CoQ (>84%) is believed to be free in the bilayer (74). A direct measurement of the amount of CoQ associated with mitochondrial membrane proteins in five different mammalian species (namely mouse, rat, rabbit, pig, and cow) has shown values between 10% and 32% of total CoQ to be protein bound (51, 93). Kinetics studies of CoQ reduction, performed in vitro on mitochondria or submitochondrial particles (inverted vesicles of the IMM), suggest that mitochondrial CoQ concentration is limiting for NADH oxidation by CI. That is, endogenous CoQ concentration in the mitochondria appears to be lower than that allowing maximal NADH oxidation rates (94–97). On the other hand, the normal concentration of CoQ in the IMM appears to be saturating for succinate oxidation by CII (94, 96, 97). Furthermore, in agreement with the kinetic data, it was shown that the incorporation of excess CoQ_10_ into native beef heart SMPs (by cosonication) induces an increase in NADH oxidation rate, but no rate increase was found for succinate oxidation (97). These are important observations because if the normal endogenous CoQ concentration is limiting, then increasing it could improve respiration, possibly even in the presence of defects in mitochondrial function. However, it should be added that most of the kinetic studies required lyophilization and the use of organic solvents (e.g., pentane) to extract and reconstitute CoQ back into membranes. These are harsh treatments that may seriously perturb the native membrane environment. For example, they could cause SC disassembly. Furthermore, the kinetic studies were mostly conducted with beef heart mitochondria. Thus, how much these in vitro observations are relevant to the in vivo situation and mitochondria of different organisms and tissues needs to be further established.

Under in vitro culture conditions, adding CoQ_10_ to cells with normal CoQ levels has only sometimes been found to have positive effects on mitochondrial respiration. For example, one study reported that supplementation with a water-soluble CoQ_10_ formulation resulted in an elevation of uncoupled cellular respiration in T67 human glioma and H9C2 rat myoblast cell lines, measured with a respirometry chamber (41). Other studies showed that treatment with CoQ_10_ had no effect on mitochondrial respiration in human skin fibroblasts and in a rat pancreatic beta cell line (INS-1), measured with a Seahorse XF Analyzer (98, 99).

Extensive studies have been conducted on the effect of CoQ deficiency on mitochondrial respiration. Overall, as expected, CoQ deficiency impairs respiratory function, but this is only observed under conditions of severe CoQ deficiency. In *E. coli*, CoQ_8_ functions in the aerobic respiratory chain in the cytoplasmic membrane, where it serves to transfer electrons from various substrate-specific dehydrogenases to two terminal oxidases, cytochrome *bo*_3_ and cytochrome *bd* (100). *E. coli* mutants without CoQ_8_ biosynthesis (Δ*ubiA*, Δ*ubiB*, Δ*ubiE*, Δ*ubiF*, Δ*ubiH*, Δ*ubiG*) or with a very low level of CoQ_8_ (<15%) (Δ*ubiX*) grow poorly on nonfermentable succinate, which is indicative of a respiratory defect (101–105). In contrast, Δ*ubiI* and Δ*ubiK* mutants that produce 15–20% of the normal level of CoQ_8_ showed no growth defect on nonfermentable carbon sources, whereas the Δ*ubiI*Δ*ubiK* double mutant, which produces no CoQ_8_, cannot grow at all on succinate (106–109). As discussed in sect. 5.1.1., yeast mutants lacking CoQ_6_ biosynthesis are also respiration defective. Interestingly, some findings suggest that CoQ is actually required to stabilize CIII, but how much the effect on CIII stability contributes to the mutant phenotype is not clear (110).

In mammalian cells, a decrease of CoQ levels below ≈60–70% of normal levels was shown to cause an inhibition of CoQ-dependent ETC activities (CI-III and CII-III) as well as a reduction in respiratory capacity and ATP levels. These observations were mostly made in dermal fibroblasts obtained from patients or in mouse embryonic fibroblasts (MEFs) from mutants with defective CoQ biosynthesis (80, 83, 111–118). For other cell types, a pronounced depression of respiration was shown for mature murine brown adipocytes and T67 human glioma cells whose CoQ content was depleted to a similar degree (≈50–60% reduction of CoQ) by treatment with a COQ biosynthesis inhibitor (119, 120). It is worth noting that it is likely that the requirement for CoQ, especially for functions other than mitochondrial respiration, varies considerably among different cell types and under different physiological and pathological conditions. Therefore, the conclusions of any study about CoQ must be viewed in the context of cell types and experimental conditions.

Studies at the tissue level are confined to the measurements of CoQ-dependent ETC functions in whole tissues or mitochondria from genetic CoQ deficiency models in mice. The effects of reduced CoQ production on ETC function have been reported for the heart, skeletal muscle, brain, kidney, and liver, which revealed significant variation in the sensitivity to CoQ deficiency across different tissues (30, 82, 90, 121–124). In the liver, an almost complete depletion of CoQ obtained by genetic means in hepatocytes causes only mild or moderate impairment of ETC function (30, 82). However, in the kidney, brain, and heart, which are known to be more energy demanding, a greater loss of respiratory function was found to always accompany severe CoQ deficiency (121, 123). Nonetheless, full respiratory function was observed in mouse kidney mitochondria with less than half of the normal level of CoQ, which is in contrast to what was observed in the heart, where ≈35% of wild-type CoQ levels were found to only sustain about one-half of full oxidative phosphorylation capacity (state 3 respiration) (121, 123). It still remains poorly understood how CoQ deficiency affects individual tissues and cell types. The variation in the relation of CoQ level to mitochondrial respiration may reflect, at least in part, tissue differences in other CoQ functions besides its role in the ETC. The heart is one of the most energy-consuming organs in the body and rich in mitochondria. Thus, likely there is an unusually high proportion of cellular CoQ associated with the ETC in cardiomyocytes. One therefore expects a high correlation between CoQ levels and mitochondrial respiration in cardiomyocytes. In contrast, as further discussed in sect. 3.3.1.2, studies of *Pdss2^kd/kd^* mutant mice showed that oxidative stress, apparently caused by impaired H_2_S oxidation, is most prominent in the kidney, and kidney failure is the primary phenotypic consequence of CoQ deficiency in this strain. A small increase in tissue CoQ_10_ level after long-term supplementation is sufficient to alleviate oxidative stress and kidney pathology of the mutant (90, 124). We postulate that, although the kidney is also relatively enriched in mitochondria, respiration is not the main consumer of CoQ. As CoQ is made in mitochondria, in the kidney the requirement of CoQ for ETC function might be relatively easily met but not the requirements for CoQ functions that require CoQ export from the mitochondria and distribution to other membranes, which might suffer more. And this might be the case for the place where the antioxidant function of CoQ is so crucially needed. For the liver, whose respiratory function appears to require very little CoQ, the explanation could be in the fact that it is in hepatocytes that dietary CoQ accumulates and is incorporated into lipoproteins (see sect. 3.2.5).

## 3. DUAL PROOXIDANT AND ANTIOXIDANT ROLES OF CoQ

A free radical is an atom or molecule that contains one or more unpaired electrons. Because of the possession of odd electrons, free radicals are usually unstable, short lived, and highly reactive (125). They can be stabilized by losing or gaining electrons through interactions with other atoms or molecules (to which they provide or from which they steal an electron). This, in turn, can alter the chemical properties of the entities with which they interact. In biological systems, free radicals are mostly oxygen- or nitrogen-containing species, namely reactive oxygen species (ROS) and reactive nitrogen species (RNS), respectively. Their production is part of normal metabolism and an inevitable consequence of aerobic life (126). Under normal physiological conditions, the intracellular levels of ROS and RNS are maintained at low concentrations. Conversely, when produced in excess, their highly reactive nature makes them potentially harmful through their ability to damage macromolecules, such as lipids, proteins, and DNA, which can lead to irreparable cell damage and death (127, 128). ROS and RNS have also been recognized as signaling molecules involved in regulating various physiological processes (129, 130). Therefore, for cell health and survival, a delicate balance must be maintained between ROS and RNS production and elimination (131, 132). In general, an antioxidant is defined as any substance that is capable of neutralizing reactive free radicals into a relatively stable unreactive form. Cells are equipped with antioxidant defense systems, consisting of both ROS-scavenging enzymes (such as superoxide dismutase and catalase) and various nonenzymatic compounds, to neutralize ROS or RNS directly or through enzymatic reactions (133).

The principal ROS produced spontaneously or enzymatically in biological systems is the superoxide anion radical (O_2_^•−^), which results from the one-electron transfer to an oxygen molecule. The discovery of superoxide dismutase (SOD), a unique enzyme that converts O_2_^•−^ into hydrogen peroxide (H_2_O_2_), helped launch the free radical theory of aging, which is centered on the accumulation of ROS-caused damage with time (134). ROS are generated by various sources, among which the mitochondrial ETC is one of the principal endogenous ROS generators. There are 12 sites in the mitochondria, with links to the ETC, that have been identified in mammalian cells to be capable of leaking electrons to oxygen and generating O_2_^•−^ (135). CoQ is one of the major ROS-generating sources in the ETC. During CoQ-mediated electron transport a partially reduced state of CoQ, ubisemiquinone (CoQ^•−^) is produced as an intermediate that can donate one electron to molecular oxygen, resulting in the formation of O_2_^•−^ at the CoQ binding sites of ETC complexes. Yet it remains to be established to what extent the amount of CoQ and its redox state contribute to total mitochondrial ROS in a given cell or cell type in a particular physiological state. On the other hand, CoQH_2_, the fully reduced form, can neutralize free radicals or regenerate other antioxidants, by giving up its own electrons, especially in the lipid membranes where it resides. In fact, CoQ is widely hailed as an antioxidant, and this property along with its key role in mitochondrial bioenergetics is the rationale given for providing CoQ_10_ as a health supplement. In this section, we summarize findings and analyses in support of the dual pro- and antioxidant role of CoQ.

### 3.1. Roles of CoQ in Mitochondrial ROS Generation

Superoxide (O_2_^•−^) is produced by one-electron reduction of molecular oxygen. SOD converts O_2_^•−^ to H_2_O_2_, which is believed to play a central role in redox signaling. However, the reactivity of H_2_O_2_ itself can in turn lead to the formation of other reactive species, such as the very damaging hydroxyl radical (^•^OH) (136). O_2_^•−^ also reacts with nitric oxide (NO^•^) to produce peroxynitrite (ONOO^•^), a toxic RNS. In fact, the reaction rate constant of O_2_^•−^ with NO^•^ (6.7 × 10^9^ M^−1^ s^−1^) is several times faster than the rate constant of the action of SOD on O_2_^•−^ (1.6 × 10^9^ M^−1^ s^−1^) (137). Thus, changes in NO^•^ levels can potentially affect O_2_^•−^ levels and hence the cellular redox state. Conversely, excessive O_2_^•−^ can have an impact on the level of NO^•^ as a signaling molecule and on nitrosative stress as a result of increased production of ONOO^•^ (138, 139).

In most cells, the ETC is the major O_2_^•−^ production site, except in phagocytes, where ROS are deliberately produced by NADPH oxidases (NOX) to produce an oxidative burst designed to kill pathogens in the phagosome (140–142). It is commonly repeated that mitochondria generate ∼90% of cellular ROS and during mitochondrial respiration ∼0.2–2% of the molecular oxygen consumed is reduced to O_2_^•−^ (143–146). However, the actual numbers are still debated. One commonly used method to measure total ROS produced by isolated intact mitochondria is to use Amplex Red dye, which, in the presence of H_2_O_2_, can be oxidized by horseradish peroxidase to give rise to a fluorescent oxidation product, resorufin (147). Although O_2_^•−^ does not readily cross membranes, SOD is provided at a high concentration in the assay’s medium to ensure that all O_2_^•−^ produced is actually converted to H_2_O_2_. It is because of this method that in the text below we sometimes refer to O_2_^•−^/H_2_O_2_ generation, although the species that is expected to be formed at a site of interest is O_2_^•−^.

Studies with isolated ETC complexes and mitochondria have identified a number of sites of ROS production including the CoQ binding sites of CI and CIII. CII is not normally a substantial source of ROS production by mitochondria. In conditions under which ROS production is induced from mammalian CII, it is the flavin site, not the CoQ binding site II_Q_, that is the most likely source of electron leak (148–150). Interestingly, it is also worth noting that among the other IMM dehydrogenases, mitochondrial G3PDH (mGPDH) was shown to be capable of producing significant amounts of ROS, with CoQ suggested to be the source of ROS in this process (150, 151).

**FIGURE 5.**
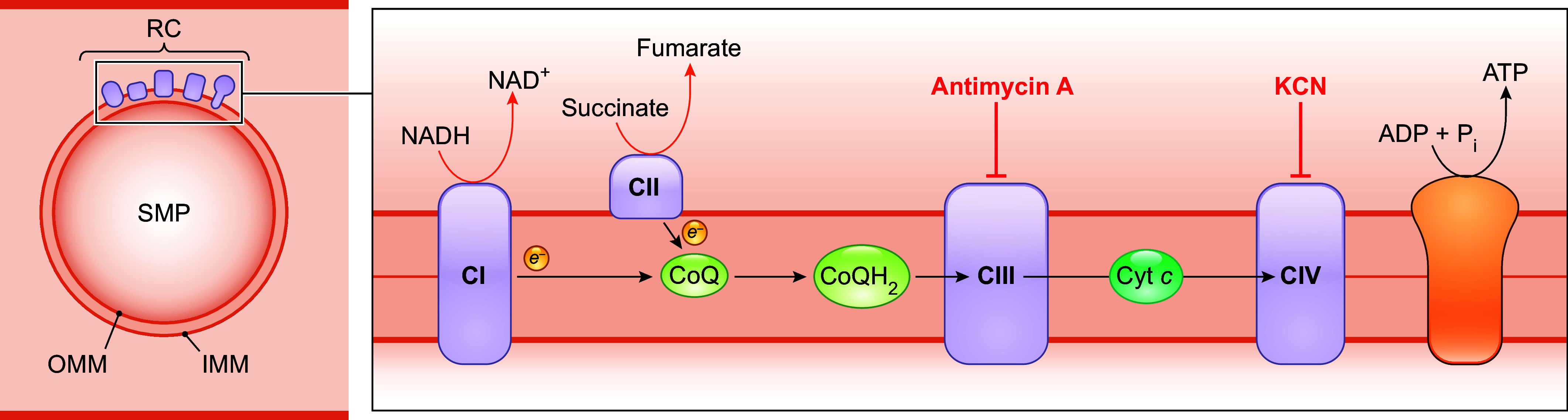
Diagram of a submitochondrial particle (SMP). A SMP is an inside-out vesicle of the inner mitochondrial membrane (IMM). It retains all the respiratory chain (RC) components, and the inversion of the IMM exposes CI and CII to the medium, allowing unrestricted access to oxidation substrates, including NADH, which could not pass through the IMM. See glossary for other abbreviations.

Reduction and oxidation of CoQ in mitochondria occur in two sequential one-electron steps (152). Inevitably, the process involves the creation of a partially reduced form of CoQ (CoQ^•−^) as an intermediate (FIGURE 1) (153, 154). As mentioned, CoQ^•−^ is a source of mitochondrial O_2_^•−^ because of its propensity to donate its unpaired electron to O_2_. Indeed, CoQ^•−^ signals were detected at the CoQ binding sites of the ETC complexes CI, CII, and CIII by electron paramagnetic resonance (EPR) (152, 154–157). The capability of CoQ to participate in O_2_^•−^ formation was first demonstrated in beef heart SMPs from which CoQ_10_ was extracted and then replenished (153). SMPs are inverted (inside out) vesicles of the IMM (FIGURE 5). As they maintain the structural integrity of the IMM and have the substrate binding sites exposed to the outer surface, they have been a valuable tool for mitochondrial functional studies. Later studies also used electron transport inhibitors specific to particular ETC complexes or sites (such as antimycin A and potassium cyanide) and, more recently, electron leak suppressors for specific CoQ sites (147, 158–160). These studies further established the contribution of different CoQ binding sites to ROS production by mitochondria during the oxidation of different substrates. Yet, not surprisingly given the chemo-physical complexity of the reactions involved, many uncertainties remain.

#### 3.1.1. Role of CoQ in ROS production from complex I.

##### 3.1.1.1. ros production by complex i during forward electron flow.

**FIGURE 6.**
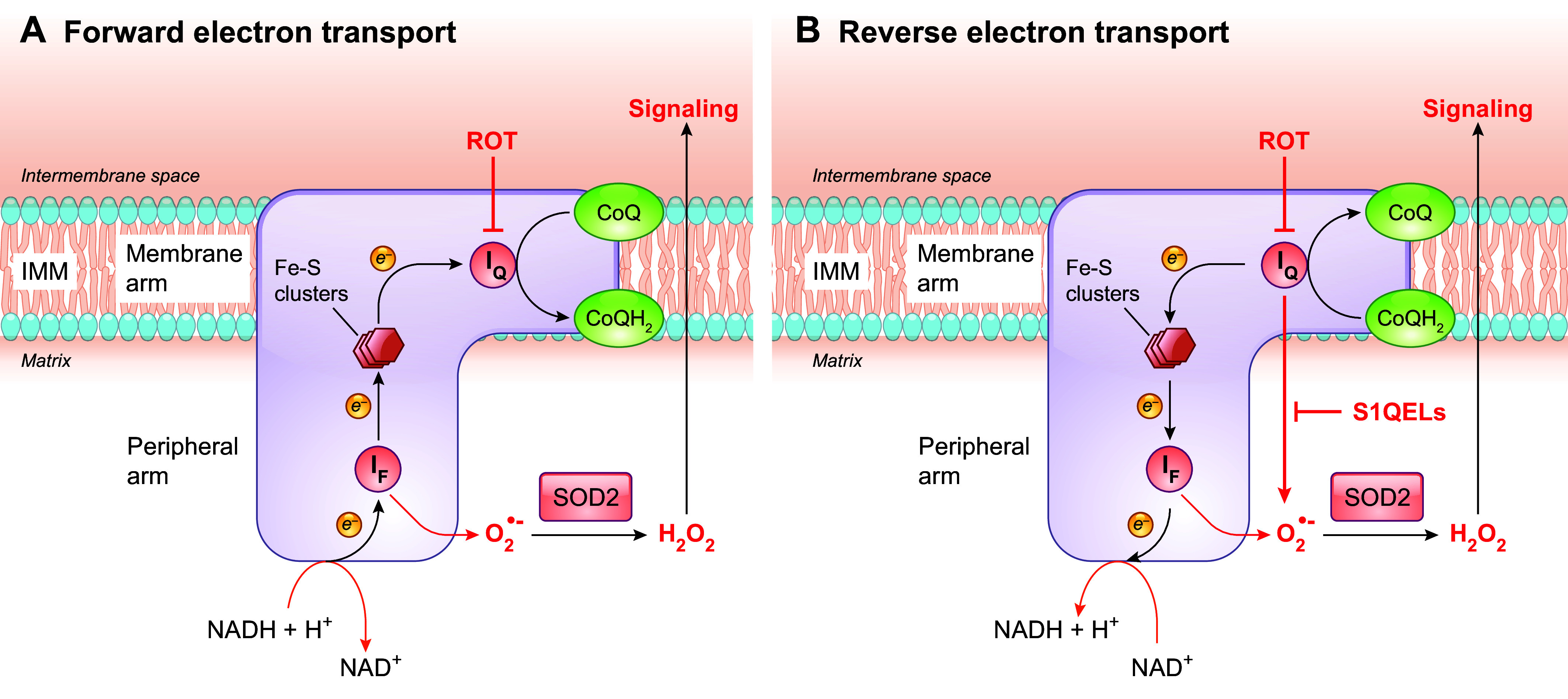
ROS production sites in CI. *A*: with forward electron transport, NADH is oxidized at the flavin mononucleotide (FMN, the I_F_ site). Electrons are then passed via several iron-sulfur (Fe-S) clusters to the CoQ binding site (I_Q_), where CoQ is reduced before it dissociates from CI. *B*: reverse electron transport occurs when electrons from an overreduced CoQ pool flow back to CI and reduce NAD^+^. The I_F_ site has been considered to be the main site of ROS production from CI under the oxidation of NADH-linked substrates. ROS production during reverse electron transport mainly originates from electron leakage from reduced CoQ formed at the I_Q_ site, but the I_F_ site has also been shown to contribute. Rotenone (ROT) blocks the flow of electrons by inhibiting the binding of CoQ to I_Q_, whereas S1QELs suppress electron leak from CoQ^•−^ to oxygen at the I_Q_ site specifically. They do this without interfering with normal electron flow, and therefore this is expected to affect ROS generation during reverse electron transport. The O_2_^•−^ produced by CI is released into the matrix, where superoxide dismutase 2 (SOD2) converts it to H_2_O_2_. See glossary for other abbreviations.

At CI, electrons move from NADH to the flavin mononucleotide (FMN, the I_F_ site) to iron-sulfur clusters, and finally to CoQ (FIGURE 6). CI from the yeast *Yarrowia lipolytica* and *E. coli* were shown to contain 0.2–1 CoQ molecules per complex (161). The CoQ reduction site (the I_Q_ site) is located at the junction of the hydrophobic membrane arm and the hydrophilic matrix arm (162). CI-linked substrates (i.e., glutamate or pyruvate in combination with malate) that feed electrons from NADH to the respiratory chain in the forward direction (starting from the I_F_ site) are generally considered to give low rates of O_2_^•−^ production, and ROS production under these conditions largely originates from the I_F_ site (FIGURE 6). In other words, the I_Q_ site normally does not dominate ROS production from CI, although this is debated (147, 163–165).

##### 3.1.1.2. ros production by complex i during reverse electron flow.

CI can also produce ROS when electrons flow through CI in the reverse direction. That is, electrons flow back from CoQH_2_ to CI and reduce NAD^+^ to NADH. Reverse electron transport (RET) has been known since the 1960s. It was first associated with ROS production in well-coupled SMPs, and it was later also shown to take place in isolated mitochondria from different tissues (166, 167). The conventional substrate to drive RET is the CII substrate succinate. In fact, in the setting of isolated mitochondria, succinate-induced RET produces the highest rate of ROS production (167, 168). Although the question of exactly where ROS are produced during RET is still controversial, it has been largely accepted that the CoQ binding site in CI, the I_Q_ site, is one of the prime loci of electron leak during RET (FIGURE 6) (135). Superoxide production by CI during RET is sensitive to the classic I_Q_ site inhibitors, such as rotenone and piericidin A (147, 169, 170). These inhibitors block the binding of CoQ to I_Q_, thus preventing the possibility of electron escape from CoQ^•−^ to oxygen (171). However, their use also inhibits reverse electron flow into CI, potentially affecting O_2_^•−^ production from other sites as well. In fact, more recently, both I_F_ and I_Q_ sites were shown to generate ROS in mitochondria isolated from rat skeletal muscle when respiring on succinate (also see sect. 3.1.1.3) (172). Notably, in recent studies by Martin Brand’s group, a novel approach was developed that allows estimation of the rate of rotenone-sensitive O_2_^•−^ production from the site I_Q_ while considering any change of ROS production by the two other key sites (the I_F_ site of CI and the Q_o_ site of CIII). With this approach it was estimated that in rat skeletal muscle mitochondria under succinate oxidation, ≈83% of O_2_^•−^ originates from the I_Q_ site, whereas that site made little or no contribution when the substrates were glutamate plus malate (163). Furthermore, in a step toward understanding ROS production in muscles in vivo, it was shown ex vivo that under conditions that mimic those in resting muscles a quarter of the total O_2_^•−^ production of rat skeletal muscle mitochondria could be attributed to the I_Q_ site, whereas the I_F_ site of CI became the dominant contributor (≈99%) under conditions mimicking intense exercise, when total O_2_^•−^ production is much lower (173).

RET is energetically uphill (i.e., against the difference of redox potentials). For I_Q_ to generate O_2_^•−^ at high rates, a highly reduced CoQ pool (to provide the electrons) and a high protonmotive force (PMF) that drives protons back into the matrix through CI are necessary (167, 174, 175). These conditions are thought unlikely to occur often under normal physiological conditions. Moreover, mitochondrial succinate levels and succinate dehydrogenase (SDH) activity in normal cells are low (176). Therefore, succinate-driven RET had initially been proposed to be minimal under normal conditions but capable of being triggered by particular stresses and thus leading to damage. One of the most cited examples is ischemia-reperfusion (I/R) injury. Succinate and other metabolic substrates accumulate during ischemia, but upon reperfusion succinate is rapidly oxidized, leading to a burst of ROS production through RET, which may contribute significantly to reperfusion injury (168, 177, 178). It is noteworthy, however, that the role of succinate-driven RET in I/R injury still remains to be fully elucidated, as it has been challenged by some studies (179). RET-dependent ROS is now thought to exist also beyond pathological conditions. It has been associated with various cellular processes, including differentiation of myoblasts into myotubes, initiation of macrophage inflammatory responses, oxygen sensing by the carotid body chemoreceptors, and uncoupling of mitochondria in brown adipose tissue (180–183). Of particular interest, ROS generation from RET has been implicated in metabolic adaptation, with the CoQ redox status acting as a sensor to adjust the respiratory chain organization for optimal efficiency. During a metabolic shift from glucose to fatty acids, which increases electron flux through FAD, it has been shown that accumulation of reduced CoQ (CoQH_2_) induces RET and results in the local generation of ROS that oxidizes CI proteins. These events, in turn, lead to CI degradation, which liberates CIII from CI + CIII SCs to receive FADH_2_ electrons from CII in order to adapt to substrate utilization (24).

##### 3.1.1.3. findings with specific suppressors of the i_q_ site electron leak.

Additional insight on CI ROS generation and CoQ has been provided by more recent studies with novel site-selective suppressors of electron leak. As mentioned above, the classic I_Q_ site inhibitors, such as rotenone, block the electron transfer from, or to, CoQ through the I_Q_ site (171). This inevitably perturbs regular electron flow and thus affects ROS production from other sites as well. In the forward direction under oxidation of NADH-linked substrates, rotenone suppresses electron flow to CIII but raises ROS production from the CI sites upstream of I_Q_ as a result of increased FMN and iron-sulfur center reduction. Conversely, during succinate oxidation, by inhibiting electrons flowing back to CI, rotenone reduces ROS production at the I_Q_ site as well as at the I_F_ site (172). In contrast, the newly developed S1QELs (suppressors of site I_Q_ electron leak) can specifically suppress electrons leaking from the I_Q_ site without interfering with normal electron flow and respiration, making them a better tool for studying ROS generation from the I_Q_ site (172, 184). With this tool, it was shown that S1QELs can suppress ROS generation from CI without affecting reverse electron flow during RET, providing better evidence for I_Q_ being a source of mitochondrial ROS when CoQ becomes overly reduced by electrons from CII or other enzymes. Moreover, with this tool, it was shown that ≈12% of the total rate of H_2_O_2_ release in C2C12 mouse myoblasts comes from the I_Q_ site. After differentiation into myotubes, total ROS release was increased, and the relative contribution of the I_Q_ site doubled (185). A similar study with several other cell lines further showed that although the absolute cellular H_2_O_2_ production rates vary considerably, the relative contribution of the I_Q_ site to total H_2_O_2_ release is similar (range 11–26%) among the diverse cell types under unstressed conditions (186). In isolated mitochondria from rat muscle incubated in media mimicking the cytosol of resting muscle, ≈12–18% of total ROS emission was sensitive to S1QELs, consistent with the above-mentioned measurements made using endogenous reporters of H_2_O_2_ levels (173, 185). S1QELs have also demonstrated a protective effect against stress-induced stem cell hyperplasia in the *Drosophila* intestine and in mice I/R injury models (184). These findings argue for the physiological significance of ROS production at the I_Q_ site. Whether all I_Q_ site ROS production is via RET is not yet clear (165, 187).

Emerging studies suggest that RET could be favored by other conditions besides a high concentration of succinate. This includes an elevation of the activity of the other metabolic pathways that feed electrons to the CoQ pool (see sect. 2.2) and a slowdown of CoQH_2_ reoxidation at CIII (24, 75, 160, 163, 188). For example, the I_Q_ site has been shown to contribute substantially (≈33%) to the total O_2_^•−^ production rate when glycerol 3-phosphate is provided as a respiratory substrate (163).

Finally, it should be mentioned that different subpopulations of CoQ^•−^ have been reported to be associated with CI (189). Rotenone-sensitive and -nonsensitive CoQ^•−^ were first described to be detectable in bovine heart purified CI upon reduction by NADH and in SMPs from bovine heart mitochondria under oxidation of NADH or succinate (157, 190). Later studies with bovine heart SMPs and isolated CI from different species also demonstrated the presence of at least two types of CI-associated CoQ^•−^ species with distinct spin relaxation behaviors: namely, the fast-relaxing ubisemiquinone (SQ_Nf_) and the slowly relaxing ubisemiquinone (SQ_Ns_) (154, 156, 191–196). The SQ_Nf_ signal is sensitive to uncouplers and rotenone and was more obvious in the presence of the ATP synthase inhibitor oligomycin (156, 194, 196). The presence of two different EPR-detectable CI-associated CoQ^•−^ species has been taken to indicate the presence of two spatially separated CoQ binding sites in CI. However, this is still debated (154, 156, 161, 192, 195, 197).

#### 3.1.2. The role of CoQ in ROS production from complex III.

##### 3.1.2.1. the q cycle.

**FIGURE 7.**
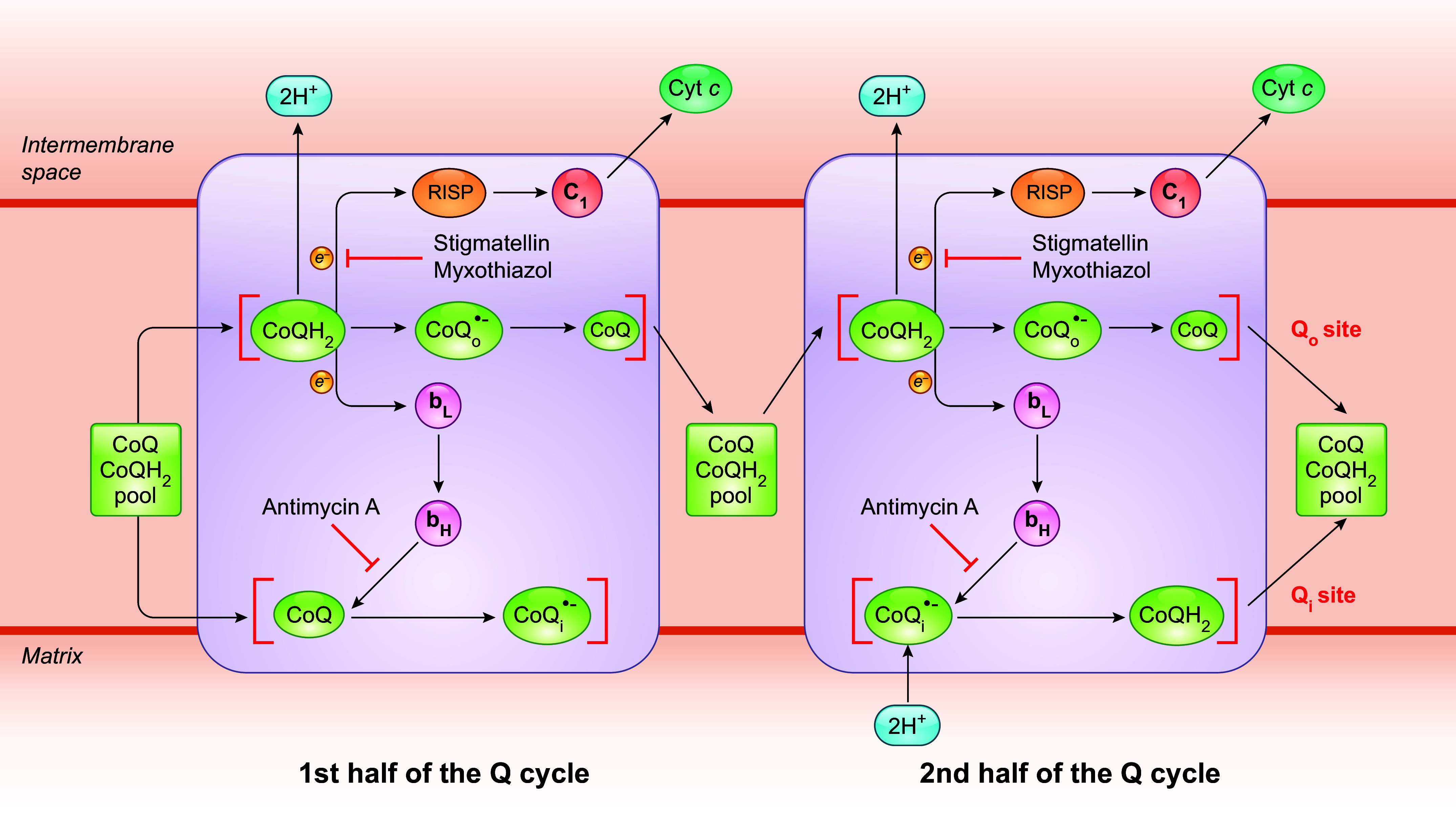
Schematics of the mechanism of the Q cycle. The Q cycle mechanism defines 2 reaction sites in CIII: CoQH_2_ oxidation (Q_o_) and CoQ reduction (Q_i_). The Q_o_ site is located between the Rieske iron-sulfur protein (RISP) and heme *b*_L_, toward the intermembrane space, whereas the Q_i_ site is close to the matrix side. It takes 2 CoQH_2_ oxidation cycles to complete the Q cycle. At first, a CoQH_2_ moves into the Q_o_ site and undergoes oxidation, with 1 electron being transferred to RISP and then to cyt *c* via cyt *c*_1_. The other electron passes through 2 *b-*type hemes (*b*_L_ and *b*_H_) across the membrane to the Q_i_ site, where a bound CoQ is reduced to CoQ^•−^ and finally to CoQH_2_. CoQ and CoQH_2_ are recycled back to the CoQ pool from the Q_o_ and Q_i_ sites, respectively, after being fully oxidized or reduced. Oxidation of each CoQH_2_ molecule releases 2 protons into the intermembrane space, and in the second half of the cycle 2 protons from the matrix are used to reduce CoQ^•−^. Given that it takes 2 electrons to fully reduce a CoQ molecule, a CoQ^•−^ intermediate is expected to be formed at the 2 distinct CoQ binding sites. Stigmatellin and myxothiazol are Q_o_ site inhibitors, whereas antimycin A blocks electron transfer from *b*_H_ to the CoQ molecule at the Q_i_ site. See glossary for other abbreviations.

CIII, which is also often called the cytochrome *bc*_1_ complex (cyt *bc*_1_), harbors two separate CoQ binding sites: Q_o_ (also called Q_p_) and Q_i_ (also called Q_n_), which face the compartments on opposite sides of the IMM. Q_o_ is located close to the outer surface of the IMM in mitochondria and on the periplasmic side in bacteria, whereas Q_i_ is facing the matrix (mitochondria) or cytoplasm (bacteria) (198). The Q_o_ and Q_i_ sites are connected electronically by two cyt *b* hemes, *b*_L_ and *b*_H_ (3). As mentioned above, CIII catalyzes a reaction of net oxidation of CoQH_2_ and reduction of cyt *c.* Oxidation of CoQH_2_ occurs at the Q_o_ site and is accompanied by a CoQ reduction reaction at the other site (Q_i_) (199, 200). The electron transfer reactions are coupled with proton movement. That is, protons are taken up by CoQ at the Q_i_ site, carried across the membrane by CoQH_2_, and released at the Q_o_ site (200). The mechanism by which electrons are transferred from CoQH_2_ to cyt *c* and by which, at the same time, protons get translocated into the intermembrane space is known as the Q cycle (FIGURE 7). This mechanism was originally proposed by Peter Mitchell almost a half-century ago but has since been modified by several groups (46, 201–203). In essence, in the version generally adopted nowadays, the Q_o_ site oxidizes two CoQH_2_ molecules in two successive steps, which provides two electrons needed to fully reduce one CoQ molecule at the Q_i_ site. In the first step, following the binding of one CoQH_2_ molecule to the Q_o_ site, the transfer of two electrons from that molecule is bifurcated. That is, one electron moves through the “Rieske” iron-sulfur protein (RISP), a component of CIII, and the cyt *c*_1_ heme before it is accepted by cyt *c.* The other electron enters the Q cycle, where it is routed through the cyt *b*_L_ and cyt *b*_H_ hemes and moves across the membrane to reach the Q_i_ site, where it acts as an electron donor to reduce CoQ to CoQ^•−^. The second step is the repeat of the first, where a new CoQH_2_ binds to the Q_o_ site and again one electron is sent through the cyt *b* chain but now it encounters a CoQ^•−^ at site Q_i_. Therefore, at the end of a complete Q cycle, as a net result two CoQH_2_ molecules are oxidized at the Q_o_ site and four electrons move through the Q cycle, resulting in the passaging of two electrons to cyt *c* and sequential reduction of one CoQ molecule to CoQH_2_ at the Q_i_ site before it is released to the CoQ pool (198). Concurrently, there is a net release of four protons (H^+^) into the intermembrane space from the two CoQH_2_ molecules oxidized at the Q_o_ site and uptake of two H^+^ from the mitochondrial matrix into the Q_i_ site.

##### 3.1.2.2. mechanism of coqh_2_ reoxidation at the q_o_ site.

A key feature of the mechanism of the Q cycle is that there are two distinct CoQ reaction sites: a CoQH_2_ oxidation center (the Q_o_ site) and a CoQ reduction center (the Q_i_ site). There has been much research aimed at understanding electron bifurcation at the Q_o_ site, which is believed to be the only known reaction of its kind in biology. One model of Q_o_ site catalysis postulates two sequential electron transfer steps: the first electron transfer from CoQH_2_ to the [2Fe-2S] cluster (ISC) of RISP, leading to the generation of a CoQ^•−^ radical intermediate, CoQ_o_^•−^, followed by the oxidation of this intermediate by heme *b*_L_ in the second reaction (3, 152, 204). So far, native CoQ or CoQH_2_ molecules have not been resolved at the Q_o_ position in X-ray crystallography studies (205, 206). Characterization of the bindings of different Q_o_ inhibitors, through observations of their effects on the absorption spectrum of heme *b*_L_ or the EPR spectrum and redox properties of the ISC, and later crystallographic studies of cyt *bc*_1_ complexes, suggests that separate functional domains might be present within the Q_o_ site_._ For example, although myxothiazol, stigmatellin, and 5-undecyl-6-hydroxy-4,7-dioxobenzothiazol (UHDBT) all bind to the Q_o_ site and prevent CoQH_2_ oxidation, myxothiazol binds to the proximal region near cyt *b*_L_, whereas the binding site of UHDBT is at a greater distance from cyt *b*_L_ and interacts specifically with the ISC of RISP and stigmatellin overlaps both the distal and proximal positions (204, 207–209). Based on studies with the inhibitors, two subsites within the Q_o_ site have been proposed: the proximal part of the site, close to the *b*_L_ heme (≈7 Å from the heme *b*_L_ and ≈12 Å from the ISC), and the distal part of the site, closer to RISP (≈7 Å from the ISC and ≈12 Å from heme *b*_L_) (208, 209).

Although the binding of a CoQH_2_ molecule to the different regions of the Q_o_ site still remains to be formally confirmed, the possibility of two separate CoQ binding regions in the Q_o_ pocket leads to a hypothesis that a CoQ_o_^•−^ intermediate generated after the first electron transfer from CoQH_2_ to RISP might diffuse from one subsite to another before the second electron transfer from the CoQ_o_^•−^ to heme *b*_L_ occurs. To be more specific, it is postulated that CoQH_2_ is first bound in the distal part of the Q_o_ pocket where CoQH_2_ transfers one electron to RISP, generating a CoQ_o_^•−^ intermediate. After having formed, the partially oxidized intermediate moves into the pocket at the proximal end of the site, near heme *b*_L_. The movement, together with a conformation change of the site, provides the mechanistic barrier for preventing any CoQ_o_^•−^ formed from further interaction with the oxidized ISC of RISP (208). Separately, an alternative double-occupancy model proposes that the Q_o_ site can accommodate two CoQH_2_ molecules at the proximal and distal regions simultaneously (209–211). However, this hypothesis has been challenged by crystal structure studies suggesting there might not be enough room in the Q_o_ site to accommodate two CoQH_2_ at the same time (205).

Experimental detection of CoQ^•−^ bound at the Q_o_ site has proven difficult. It has been variously interpreted as indicating a high instability of CoQ_o_^•−^, or extreme difficulty in its detection, possibly due to magnetic coupling between CoQ_o_^•−^ and the reduced ISC of RISP, or the possibility that, if both electron transfers to RISP and heme *b*_L_ occur simultaneously, no CoQ_o_^•−^ intermediate would actually be formed (3, 152, 204, 212). However, several recent studies have reported successful CoQ_o_^•−^ detection when its reoxidation is blocked, by the use of either a Q_i_ site-specific inhibitor or a heme *b*_H_ knockout by genetic means (155, 213, 214). These findings, though not accepted by everyone, argue against a Q_o_ site model of simultaneous two one-electron transfers from CoQH_2_.

##### 3.1.2.3. coq reduction at the q_i_ site.

The Q_i_ site is at the end of the cyt *b*_L_-cyt *b*_H_ electron transfer chain and is situated near the matrix side of mitochondria and the cytoplasmic side of the bacterial membrane, where protons are taken up during catalysis for reduction of CoQ (215). In contrast to the situation with the Q_o_ site, X-ray and cryo-electron microscopy (cryo-EM) structural studies of the cyt *bc*_1_ complexes have documented a CoQ occupancy within the Q_i_ site (198, 206, 216–218). Mechanistically, heme *b*_H_ reduces CoQ to CoQ_i_^•−^ after an electron is transferred from the first CoQH_2_ that moves to the Q_o_ site and reduces CoQ_i_^•−^ to CoQH_2_ after a second oxidation event. As the CoQ_i_^•−^ intermediate that is formed after every first CoQH_2_ oxidation at Q_o_ needs to remain at the Q_i_ site until the second CoQH_2_ oxidation takes place, CoQ_i_^•−^ is predicted to be more tightly bound than CoQ_o_^•−^. This has been regarded as a plausible explanation of why CoQ_i_^•−^ intermediate is easier to detect than CoQ_o_^•−^. Antimycin A, the best-known Q_i_ site inhibitor, blocks the electron transfer from cyt *b*_H_ to the Q_i_ site and thus inhibits the reduction of the CoQ pool.

##### 3.1.2.4. ubisemiquinone is the source of mitochondrial ros generated by complex iii.

We have discussed that an unstable CoQ^•−^ can directly reduce O_2_, forming O_2_^•−^, and that the CoQ^•−^ formed at the Q_o_ site is less stable than that at the Q_i_ site (219, 220). Moreover, only the CoQ^•−^ at the Q_o_ site is thought to have a sufficiently low redox potential to be able to give an electron to oxygen (221). Thus, the Q_o_ site, not the Q_i_ site, is the most likely electron donor in the production of O_2_^•−^ at CIII. This has been demonstrated experimentally in various types of preparations (intact mitochondria, SMPs, and isolated cyt *bc*_1_ complexes) by the use of inhibitors that bind specifically to only one of the two distinct CoQ-binding sites (3, 147). Generally, Q_i_ site inhibitors (the most classic one being antimycin A) are found to induce high rates of ROS production, whereas Q_o_ site inhibitors (e.g., stigmatellin and myxothiazol) suppress antimycin A-induced ROS production (209, 222, 223). These findings can be best explained by the Q cycle mechanism of CIII. In the presence of antimycin A, hemes *b*_H_ and *b*_L_ cannot be reoxidized by electron transfer to the Q_i_ site. Thus, CoQ^•−^ formed upon the first oxidation of the first CoQH_2_ at the Q_o_ site is unable to donate electrons to hemes *b*_L,_ resulting in longer residence of CoQ_o_^•−^ at the Q_o_ site and greater probability of electron transfer to oxygen resulting in O_2_^•−^ formation (224). Q_o_ site inhibitors stigmatellin and myxothiazol on the other hand prevent the formation of CoQ^•−^ at the Q_o_ site and thus eliminate the stimulation of O_2_^•−^ production by antimycin A. But, notably, in contrast to stigmatellin, which completely blocks O_2_^•−^ production by CIII, myxothiazol only partially (by ≈70%) prevented antimycin A-induced O_2_^•−^ production (225, 226). Furthermore, the effect of myxothiazol on its own also leads for O_2_^•−^ formation. The rate of myxothiazol-induced O_2_^•−^ production is lower than that observed with antimycin A and is highly sensitive to stigmatellin as well (209, 225, 227). Stigmatellin, as mentioned above, binds to the Q_o_ site in the distal part of the site, near RISP. A crystal structure with bound stigmatellin shows it binding in the same position as CoQH_2_, to a histidine ligand of the ISC of RISP via a hydrogen bond. This would be expected to exclude CoQH_2_ and prevent CoQ_o_^•−^ formation [Crofts (198)]. Myxothiazol, however, is binding to the proximal part of the Q_o_ site, near cyt *b*_L_ (209, 216). This suggests that myxothiazol does not entirely exclude CoQH_2_ from binding at Q_o_. Overall, these findings suggest a model in which the distal part of the Q_o_ site pocket is the main source of CoQ^•−^ formation at Q_o_ but both the distal and proximal parts of the CoQ binding sites transiently contain CoQ^•−^, with the potential to reduce oxygen and contribute to CIII ROS production.

With regard to the relative contribution of the Q_o_ site to mitochondrial ROS, it was shown that, in rat skeletal muscle mitochondria, the Q_o_ site made only a modest contribution (≈10%) to the total O_2_^•−^ production under succinate oxidation but accounted for ≥30% of the total production rate when CI substrates (glutamate or malate) or substrates of β-oxidation (palmitoylcarnitine plus carnitine) were being oxidized. This estimate was made possible by using the state of cyt *b*_L_ reduction as an endogenous reporter for the rate of O_2_^•−^ formation at the Q_o_ site (163, 228). Under ex vivo conditions that mimic rest or mild aerobic exercise, ≈15% of total O_2_^•−^ was produced from the Q_o_ site, whereas very little or no ROS was produced from the Q_o_ site under conditions that mimic intense aerobic exercise in skeletal muscles (173). These findings are in good agreement with the reported value obtained with several S3QELs (Suppressors of site III_Qo_ electron leak), which selectively suppress O_2_^•−^ formation from the Q_o_ site but do not block electron flow or affect OXPHOS (185, 229). S3QELs were also used to assess the contribution of Q_o_-derived O_2_^•−^ to cell physiology and pathology. In various cell types, treatment with S3QELs was shown to suppress the total rate of extracellular H_2_O_2_ release by a similar extent within a 13–30% range, despite the fact that the absolute cellular H_2_O_2_ production rates vary greatly among the diverse cell types (141, 185, 186). In vivo studies with *Drosophila* reported that feeding S3QELs protects against ROS-induced stem cell hyperplasia in the intestine, and S3QELs also decrease diet-induced intestinal barrier disruption in both flies and mice, suggesting a key role for Q_o_ ROS in these pathologies (184, 230).

Finally, it is important to note that, in contrast to most mitochondrial ROS forming sites, which release O_2_^•−^ into the matrix, the Q_o_ site emits at least some O_2_^•−^ directly into the IMS (145, 160, 186, 231, 232). As mentioned above, O_2_^•−^ cannot easily diffuse through the IMM, so after being released into the matrix it is mostly confined to the matrix, where it can directly oxidize the Fe-S clusters of enzymes such as aconitase or be converted to H_2_O_2_ by SOD2 (233). H_2_O_2_ can then diffuse out of the mitochondria and function as a signaling agent. A minor fraction of cytosolic SOD1 is found in the IMS, where it can catalyze the dismutation of O_2_^•−^ into H_2_O_2_ (234–236). Unlike the IMM, the OMM is porous, allowing the easy passage of small molecules including H_2_O_2_. Moreover, some of the O_2_^•−^ in the IMS may also escape into the cytosol via voltage-dependent anion channels in the OMM (237). Consequently, O_2_^•−^ and H_2_O_2_ produced in the IMS should have easier access to the cytosol, where they can act as signaling molecules. For example, several studies showed that ROS from the Q_o_ site is required for the stabilization of the HIF-1α protein during hypoxia, although conflicting findings have also been published (238, 239).

### 3.2. The Antioxidant Function of CoQ

It is well known that by virtue of its chemical properties, CoQ can act as an antioxidant. In its reduced state, it can give up its electrons to free radicals, thus stabilizing them and neutralizing their reactivity (240, 241). CoQ is also known for its ability to help regenerate other antioxidants such as vitamin E back to their active states. CoQ appears present in all cellular membranes, where its reduced form (CoQH_2_) can be restored from the oxidized form (CoQ) by various enzymatic mechanisms, and its primary antioxidant role is believed to act on lipid radicals generated when lipids are peroxidized (12). In fact, significant amounts of CoQH_2_ can be measured in various membrane fractions (including the plasma membrane and endomembranes) (242, 243). The antioxidant activity/capacity of CoQ is doubtlessly dependent on both the total amount of CoQ and the ratio between reduced and oxidized forms (CoQH_2_/CoQ). In the IMM, where CoQ passes through oxidation and reduction reactions during electron transport, the balance of reduced and oxidized CoQ is maintained by the activity of the respiratory chain. Yet what affects total CoQ content and the ratio between CoQH_2_ and CoQ in the IMM remains largely elusive. Outside the mitochondria, the plasma membrane redox system (PMRS), also called the trans-plasma membrane electron transport (PMET) system, is the best-understood mechanism that involves the redox cycling of CoQ.

#### 3.2.1. Redox cycling of CoQ in the plasma membrane redox system.

**FIGURE 8.**
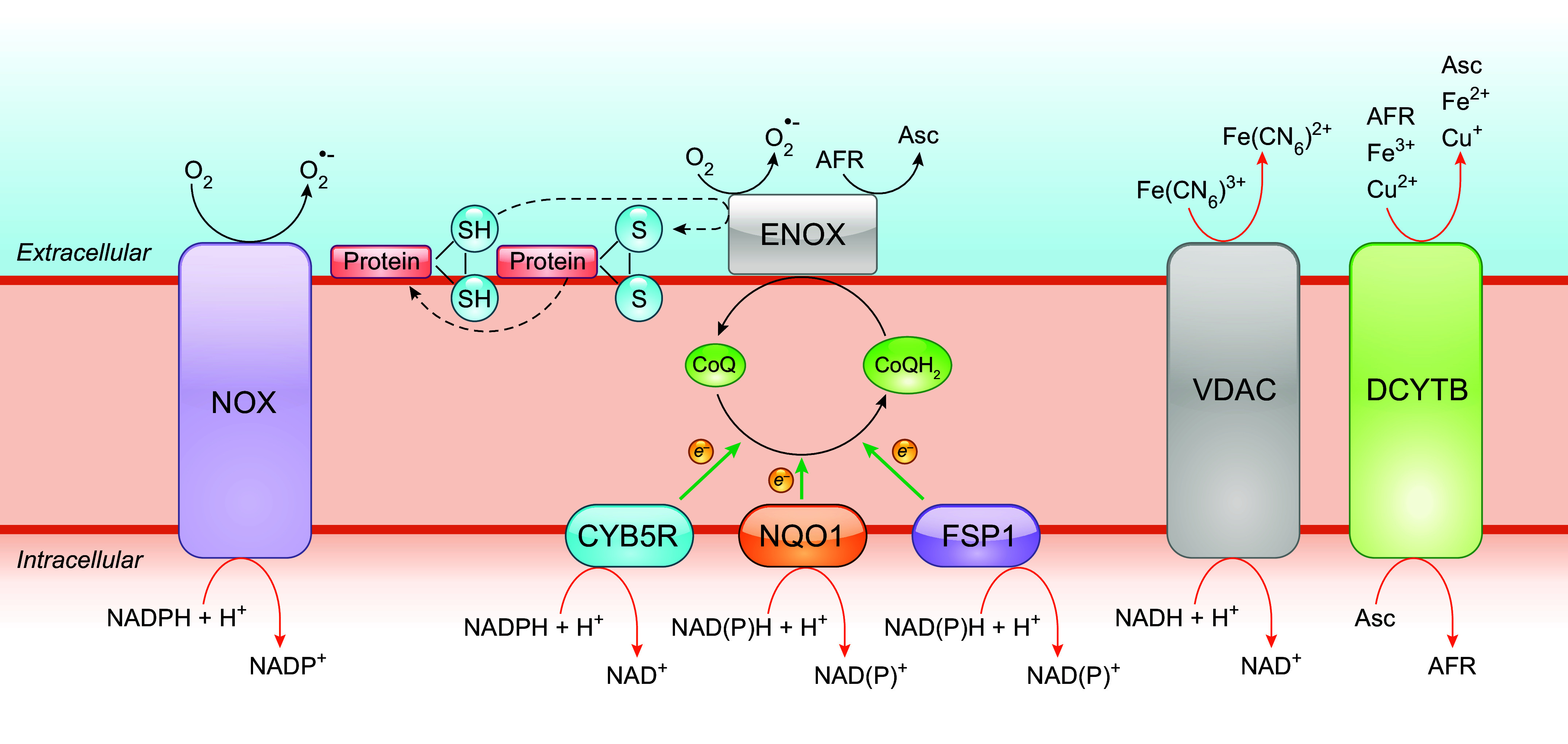
Role of CoQ in the plasma membrane redox system (PMRS). The PMRS consists of multiple component operations that result in electron transfer from cytosolic reducing equivalents to extracellular electron acceptors. NADH-cytochrome *b*_5_ reductase (CYB5R), NAD(P)H:quinone oxidoreductase 1 (NQO1), and ferroptosis suppressor protein 1 (FSP1) are CoQ reductases that oxidize NADH or NADPH to reduce CoQ. The cell surface protein ENOX is the terminal oxidase by catalyzing electron transport from CoQH_2_ to extracellular electron acceptors, including oxygen (O_2_) and ascorbyl (monodehydroascorbate) free radical (AFR). Besides oxidizing CoQH_2_, ENOX also possesses an alternative activity, which is catalyzing protein disulfide-thiol interchange. Other enzymes of the PMRS include the NADPH/NADH oxidase (NOX) that directly catalyzes the 1-electron transfer from cytosolic NADPH to molecular oxygen, the voltage-dependent anion-selective channel (VDAC) that reduces extracellular ferricyanide using NADH as electron donor, and the duodenal cytochrome *b* (DCYTB) that utilizes ascorbate (Asc) in the cytosol as an electron donor to reduce either extracellular ferric iron (Fe^3+^), cupric copper (Cu^2+^), or AFR. See glossary for other abbreviations.

The PMRS operates in all living cells, from bacteria to humans, although its components may vary depending on cell type (244, 245). Mainly, it allows electrons from intracellular substrates to flow outward to extracellular electron acceptors, by a process that is centered on CoQ (7, 246, 247). Physically, the classic description of the PMRS in mammalian cells consists of cytosolic and plasma membrane-associated oxidoreductases that transfer electrons, derived from NADH or NADPH, to the membrane-embedded intermediate electron carrier CoQ and finally to extracellular electron acceptors such as oxygen (248) (FIGURE 8). The enzymes involved in CoQ-dependent PMRS activity include NADH-cytochrome *b*_5_ reductase (CYB5R), NAD(P)H:quinone oxidoreductase 1 (NQO1), formerly known as DT-diaphorase, and the recently identified ferroptosis suppressor protein 1 (FSP1) (249–253). CYB5R, present at the inner surface of the plasma membrane (also in mitochondria and the endoplasmic reticulum), catalyzes the one-electron reduction of CoQ by NADH, resulting in the formation of ubisemiquinone (CoQ^•^**^−^**), which can be further reduced to CoQH_2_, whereas NQO1 reduces CoQ with either NADH or NADPH by a two-electron reaction, directly to CoQH_2_ (245, 254). NQO1 is known to be primarily cytosolic but can be translocated to the inner surface of the plasma membrane under stress conditions (255). FSP1 (also known as AIFM2), like NQO1, utilizes both NADH and NADPH as electron donors to reduce CoQ. The NH_2_ terminus of FSP1 contains a canonical myristoylation site that is essential for its plasma membrane localization (252, 253, 256). The reduced CoQH_2_ can then shuttle electrons to the cell surface NADPH/NADH oxidase (NOX) (external NOX, ENOX) that is able to reduce oxygen to yield O_2_^•–^ or use the oxidized form of ascorbate (Asc), the ascorbyl free radical (AFR), as the terminal electron acceptor (246, 257–259). Other PMRS activities that do not involve CoQ include electron transfer by the transmembrane NOX proteins, the duodenal cytochrome *b* (DCYTB), or the NADH:ferricyanide reductase [known as the voltage-dependent anion-selective channel (VDAC)] (260–265) (FIGURE 8). Of note, it was suggested that CoQ might also function as a physiological substrate of VDAC (261).

One of the recognized roles of the PMRS, perhaps the most important one, is the control of the cytosolic NAD^+^-to-NADH ratio, thus modulating the cellular energy balance and redox homeostasis (see also sect. 4.4) (245). The PMRS is also implicated in iron uptake and immune cell function (246). Furthermore, the PMRS allows for the reduction of CoQ at the expense of intracellular reducing equivalents. This reduced pool of CoQ is believed to play an important role in antioxidant protection, mainly against membrane lipid peroxidation and also via regenerating other antioxidants (see below).

#### 3.2.2. Antioxidant role of CoQ by regenerating vitamin C and E.

##### 3.2.2.1. role of coq in vitamin c regeneration.

**FIGURE 9.**
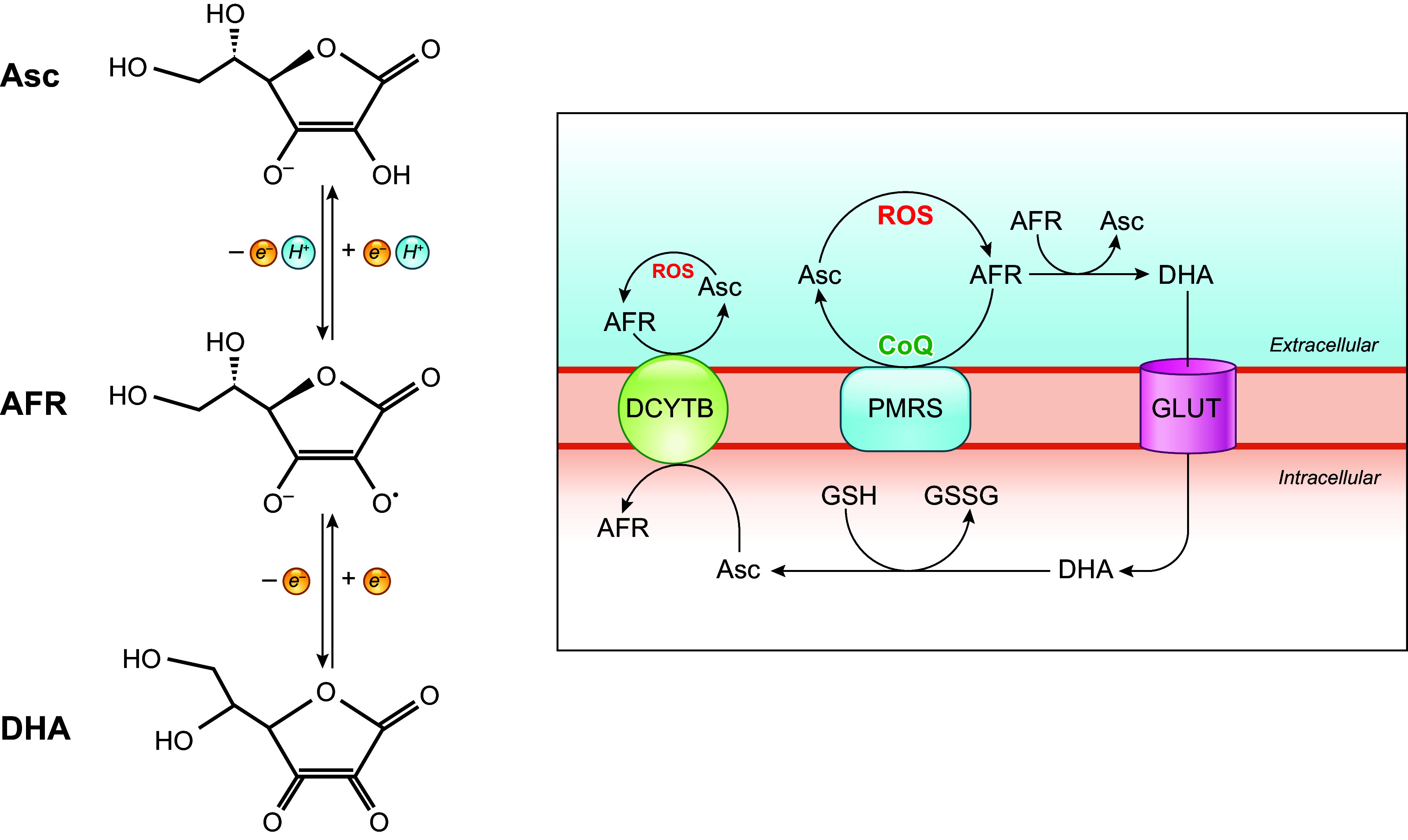
Redox metabolism of ascorbate. Ascorbate (Asc) can undergo 2 consecutive 1-electron oxidations that generate the ascorbyl free radical (AFR) as an intermediate and the complete oxidation product dehydroascorbate (DHA). Free radical-mediated oxidative stress results in the oxidation of Asc, yielding AFR. The CoQ-dependent plasma membrane redox system (PMRS) transfers reducing equivalents from intracellular electron donors to AFR outside of the cell, converting AFR back to reduced Asc. Two molecules of AFR can react with each other to form 1 DHA and 1 Asc. DHA made extracellularly can be transported through glucose transporters (GLUT) into the cell, where it can be recycled back to Asc using glutathione (GSH) as a reductant, yielding glutathione disulfide (GSSG). Extracellular AFR can also be reduced by duodenal cytochrome *b* (DCYTB) using intracellular Asc as an electron donor in some species and tissues. See glossary for other abbreviations.

As indicated above, in addition to oxygen, oxidized vitamin C (VC) is one of the extracellular targets of the PMRS. Vitamin C, also known as ascorbic acid or ascorbate (Asc), is the most abundant water-soluble antioxidant in the extracellular fluid. Because of its low redox potential (+0.282 V at pH 7), Asc can readily donate electrons to stabilize free radicals (266). Asc reacts with all kinds of biologically generated radicals (267). It is a particularly effective scavenger of aqueous peroxyl radicals (^•^OH), with a rate constant of 7.9 × 10^9^ M^−1^ s^−1^ to 1.1 × 10^10^ M^−1^ s^−1^ (268, 269), although, according to its rate constant toward O_2_^•−^ (2.7 × 10^5^ M^−1^ s^−1^), it is not an effective scavenger of O_2_^•−^. Nonetheless, the reaction between O_2_^•−^ and Asc is likely to happen in vivo given the abundance of Asc in tissues (270–272). For its antioxidant action, Asc preferably serves as a one-electron donor, generating a relatively stable AFR (also written as Asc^•−^) (FIGURE 9) (273, 274). Losing the second electron from AFR leads to its transformation into dehydroascorbic acid (DHA). Asc cycles predominantly between the fully reduced form and AFR. DHA is produced mainly by disproportionation of AFR, reactions of 2 AFR to yield 1 DHA and 1 ascorbate molecule (FIGURE 9) (275). More importantly, as AFR is the major product of Asc oxidation, the ability to recycle AFR back to the reduced form is most crucial for the regeneration of antioxidant Asc. As indicated above, in addition to oxygen, AFR is a terminal electron acceptor in the PMRS as well. Thus, the CoQ-dependent PMRS serves to reduce AFR in the extracellular space and restore the Asc pool. But other mechanisms have also been identified. DHA produced extracellularly can be transported into the cell, where it can be reduced back to Asc, for example by glutathione-dependent DHA reductase (275, 276). In human erythrocytes, it was shown that DCYTB can contribute to extracellular Asc recycling by using intracellular Asc as an electron donor (FIGURE 9) (277–279). Moreover, Asc export to the extracellular space has been reported, which could help replenish the Asc pool (277, 278).

A number of experimental findings point to an important role of the CoQ-dependent PMRS in ascorbate regeneration. CoQ extraction (with heptane) from lyophilized plasma membranes (from pig liver or K562 human leukemia cells) results in inhibition of NADH-ascorbate free radical (AFR) reductase (250). Incorporation of CoQ_10_ stimulates NADH-AFR reductase activity, and supplementation of K562 cells with CoQ_10_ is associated with a dose-dependent increase of extracellular ascorbate stabilization, which indicates a higher rate of plasma membrane AFR reduction (280–282). Furthermore, a yeast *coq3* mutant defective in CoQ_6_ production was documented to have diminished NADH-AFR reductase activity and reduced extracellular ascorbate stabilization, and both activities were rescued after restoration of CoQ_6_ levels (283, 284). However, the physiological and pathological significance of the plasma membrane CoQ in contributing to extracellular antioxidant defense by allowing ascorbate regeneration needs further study and clarification.

##### 3.2.2.2. role of coq in vitamin e regeneration.

CoQ is also believed to play a role in regenerating another primary exogenous antioxidant: vitamin E (VE), also known as α-tocopherol. Like CoQ, VE is lipophilic and located in membranes and lipoprotein fractions (285). A role for VE in membrane structural stabilization has been generally recognized, although how it is achieved is not yet well defined (286). Another important function of vitamin E is to act as an antioxidant protecting membrane lipids against peroxidation by scavenging lipid radicals.

**FIGURE 10.**
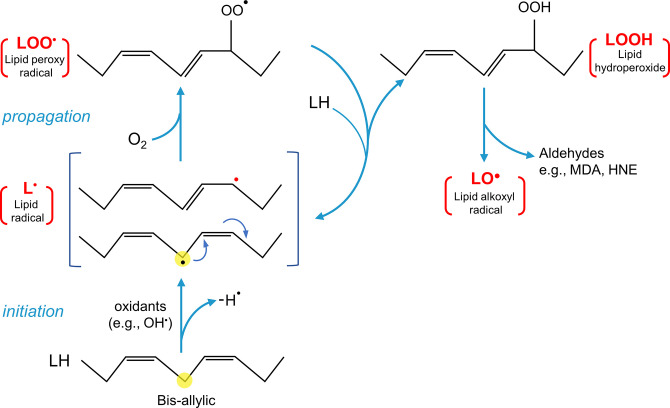
The lipid peroxidation process. It is initiated by the radical-mediated abstraction of a hydrogen atom from a bis-allylic methylene group in a lipid (LH). This leads to the formation of a carbon-centered lipid radical (L^•^), which undergoes molecular rearrangement and then reacts with oxygen to form a peroxyl radical (LOO^•^). In turn, LOO^•^ propagates a chain reaction through the formation of a new L^•^ by hydrogen abstraction from other lipids, while it itself is converted to a lipid hydroperoxide (LOOH). Decomposition of LOOH yields lipid alkoxyl (LO^•^) and the formation of aldehydes such as malondialdehyde (MDA) and 4-hydroxynonenal (4-HNE) and other secondary and end products of lipid peroxidation.

Lipids in the form of polyunsaturated fatty acids (PUFAs) are the major components of cellular membranes. Owing to their multiple carbon–carbon (C=C) double bonds, PUFAs are especially labile to oxidation, given the relative ease with which hydrogen atoms can be removed from the bis-allylic methylene (–CH_2_–) between double bonds (287). Free radicals (such as OH^•^) can rip off hydrogen from the methylene group in PUFAs, resulting in the formation of a carbon-centered lipid radical that upon rearrangement forms a stable carbon-centered radical (L^•^). As L^•^ has an unpaired electron on carbon, this makes it reactive and inclined to react with oxygen, yielding a lipid peroxyl radical (LOO^•^). LOO^•^ has a long half-life (10 s) and in turn can abstract a hydrogen atom from another adjacent lipid molecule to produce a lipid hydroperoxide (LOOH), while producing a new L^•^. This new L^•^ can then initiate a new oxidation reaction (288). Thus, the whole process becomes self-propagating and will keep going until the substrate is consumed or termination occurs (289). The process by which lipids are attacked by radicals leading to the formation of LOOH is called lipid peroxidation (FIGURE 10). It is one of the most important types of oxidative damage in biological systems. Because the process is self-perpetuating, the effect of the original free radical will be amplified, leading to numerous modified lipids. This oxidative degradation of membrane lipids can perturb the bilayer structure and alter membrane properties such as fluidity, permeability, conductivity, and ion transport (290). Furthermore, although LOOH is stable compared to lipid radicals, by the process of Fenton chemistry when interacting with a metal ion, it can decompose into a lipid alkoxyl radical (LO^•^) that may continue to propagate new peroxidation reactions. In addition, the decomposition of LOOH can form various aldehydes that are potentially toxic. For example, the LOOH breakdown products malondialdehyde (MDA) and 4-hydroxynonenal (4-HNE) are capable of damaging membrane-bound receptors and enzymes, which in turn can trigger cell death (291, 292). There are many excellent reviews on various aspects of lipid peroxidation, such as Refs. 289, 293–295, to which readers can turn for more information.

**FIGURE 11.**
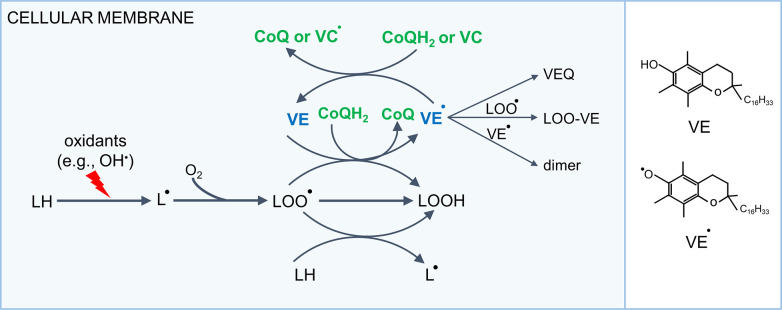
Antioxidant action of vitamin E (VE) against lipid peroxidation. VE scavenges lipid peroxyl radical (LOO^•^) before it attacks other lipids (LH) to form lipid hydroperoxide (LOOH) and a new lipid radical (L^•^), by which it terminates lipid peroxidation chain reactions. This leaves behind the vitamin E radical (VE^•^). VE^•^ can be converted back to the reduced antioxidant form by CoQ or vitamin C (VC). VE^•^ can also react with another LOO^•^, forming poorly reactive nonradical adducts, decay by reaction with another VE^•^ molecule to give inactive dimers, or be completely oxidized to vitamin E quinone (VEQ). VC^•^, vitamin C radical. See glossary for other abbreviations.

The chain reaction of lipid peroxidation is terminated by lipid radicals that take part in the chain reaction, quenching each other or combining with a similar radical to form nonradical products, or by antioxidants. The intermediate product LOO^•^ is responsible for the propagation of the lipid peroxidation process. VE, by virtue of its ability to donate electrons to LOO^•^ species, is able to neutralize LOO^•^ while it itself is oxidized to a vitamin E radical (VE^•^), also known as an α-tocopherol radical (FIGURE 11) (296–298). VE^•^ is not very reactive and can be regenerated to VE. Thus, VE is known as a chain-breaking antioxidant, as it intercepts LOO^•^ and thus interrupts lipid peroxidation cascades. The cell’s ability to regenerate VE from its radical form back to its reduced native state determines the antioxidant activity of VE. The one-electron redox potential for CoQH_2_ [CoQH_2_/CoQ^•−^ = −0.2 V] is more negative than that for vitamin E [VE/VE^•^ = 0.5 V], and therefore CoQH_2_ is capable of reducing oxidized VE, thus regenerating its activity (266). VE can also be regenerated by Asc, but it has been claimed that regeneration by CoQ is favored (10). Other potential fates of VE^•^ include reacting with another VE^•^ or LOO^•^ forming nonradical stable products or being further oxidized to VE quinone (288, 299).

By electron spin resonance (ESR) and stopped-flow spectroscopy, it was shown that VE^•^ disappears after the addition of CoQ_10_H_2_ (but not CoQ_10_) (300, 301). Moreover, mitochondrial CII integrated into proteoliposomes slowed the accumulation of the VE^•^ ESR signal (induced by lipoxygenase-catalyzed linolenic acid oxidation) only when succinate and CoQ_10_ were both also incorporated into these liposomes (302). Similarly, in rat liver SMPs, the simultaneous addition of NADH and CoQ_1_ was shown to produce a maximum decrease of VE^•^ signals (303). These observations can be interpreted if CoQH_2_ reduces VE^•^ and thus mitochondrial enzyme-linked reduction of CoQ to CoQH_2_ prevents accumulation of VE^•^.

#### 3.2.3. Direct antioxidant properties of CoQ by scavenging lipid radicals.

Shortly after the discovery of CoQ in 1961, it was suggested that CoQ itself can act as an antioxidant against lipid peroxidation. It was proposed that it could directly quench LOO^•^ as follows (304):

(*1*) $${\text{CoQH}}_{2}+{\text{LOO}}^{•}\to {\text{CoQ}}^{•-}+\text{LOOH}$$

(*2*) $${\text{CoQ}}^{•-}+{\text{LOO}}^{•}\to \text{CoQ}+\text{LOOH}$$

where CoQH_2_ undergoes one electron oxidation to give LOOH and CoQ^•^**^−^** (*reaction 1*). The reaction of CoQ^•^**^−^** with another LOO^•^ produces LOOH and a fully oxidized CoQ (*reaction 2*). Because, as VE, CoQH_2_ eliminates LOO^•^ by direct scavenging, thus terminating the propagation of peroxidation, it is considered to be primarily a chain-breaking antioxidant (12). Of note, the possibility that CoQH_2_ may also be able to quench L^•^, thus inhibiting the initiation of lipid peroxidation, has also been considered but is still under study (240, 305).

The chemical reactivity of CoQH_2_ with peroxyl radicals was demonstrated in solvent solutions, and its reactivity toward peroxyl radicals was shown to be lower than that of VE (300, 306, 307). This means that VE scavenges peroxyl radicals faster than CoQ. In liver microsomal and mitochondrial membranes, it was shown that, in comparison to VE, treatment with externally added CoQ_9_ and CoQ_10_ showed much weaker inhibition of lipid peroxidation induced by Fe^2+^ plus ascorbate (or by Fe^2+^ plus NADPH or *tert*-butyl hydroperoxide) (303). Considered together with the reported observation that the concentrations of CoQ in membranes are usually close to, or lower than, those of VE, CoQ seems unlikely to be the major lipid-soluble antioxidant in cellular membranes but might instead defend against lipid peroxidation by regenerating VE (303, 308). However, it is noteworthy that in beef heart SMP preparations that contain no detectable amount of VE, inhibition of MDA formation was reported to be observed again after reincorporation of CoQ_10_, indicating some direct effect of CoQ (309).

#### 3.2.4. Other experimental evidence for the antioxidant effects of CoQ.

Most available direct evidence for an antioxidant effect of CoQ came from studies using in vitro subcellular systems such as liposomes and SMPs. Liposomes are small artificial spherical structures, with a lipid bilayer primarily made by the self-assembly of phospholipids. Thus, they have been actively used as a simple model of cell membranes. Lipophilic molecules such as CoQ can be incorporated into the lipid bilayers of liposomes. Indeed, the addition of CoQH_2_ to liposomes was shown to inhibit the formation of lipid peroxidation induced by different free radical generators. For example, in liposomes made of phosphatidylcholine (PC, a major type of phospholipid of cell membranes), PC hydroperoxide formation induced by 2,2′-azobis(2,4-dimethylvaleronitrile) (AMVN), a lipophilic free radical initiator, was decreased when CoQ_10_H_2_ was added (at a molar ratio to phospholipids similar to that in human tissues), and no effect was seen after addition of the oxidized form CoQ_10_ (304). Similarly, in reconstituted egg yolk PC liposomes containing NADH-cytochrome *b*_5_ reductase or DT-diaphorase and a high amount of CoQ_10_ (50 µM), incubation with NADH led to an increased pool of CoQ_10_H_2_ and a simultaneous reduction of lipid peroxidation induced by exposure to free radical-generating compounds, such as 2,2′-azobis(2-amidinopropane) dihydrochloride (AAPH) or AMVN (249, 310).

A correlation between CoQ and lipid peroxidation levels was also demonstrated in SMPs. As they retain OXPHOS activity when provided with oxidizable substrates (NADH or succinate) to provide reducing equivalents, they are capable of generating CoQH_2_ from CoQ and the conversion of CoQ to CoQH_2_ can be further maximized by simultaneously blocking its oxidation at CIII (FIGURE 5). For example, CoQ can be kept in a highly reduced state (>80%) in SMPs under oxidation of NADH or succinate in the presence of CIII inhibitors (e.g., antimycin A) or CIV inhibitors (e.g., potassium cyanide, KCN) (240).

MDA is one of the most frequently used biomarkers of lipid peroxidation. In bovine heart SMPs, it was shown that MDA formation induced by the addition of NADH or NADPH in the presence of ADP and Fe^3+^ was inhibited by succinate, and the effect of succinate was further enhanced by antimycin A and KCN (311). Similar findings were reported in studies using ADP/Fe^3+^ plus Asc to induce lipid peroxidation (309). In contrast, the antiperoxidative effect of CoQ was not observed in beef heart SMPs from which most CoQ was removed, but it appeared again upon reincorporation of CoQ (240, 309, 311, 312).

Finally, given the high hydrophobicity and membrane location of CoQ, most in vitro studies of its antioxidant properties have been focused on lipid peroxidation. However, a protective effect of endogenous CoQ against protein and DNA oxidation has also been suggested. Incubation of isolated rat liver mitochondria with ADP/Fe^3+^/Asc resulted in substantial protein oxidation (measured by protein carbonylation) and an increase in the amount of 8-hydroxy-2-deoxyguanosine (8-OHdG), a product of oxidative damage to DNA. Both were shown to be inhibited by the addition of succinate and antimycin A (313). Similarly, increasing the pool of CoQH_2_ in beef heart SMPs in the presence of succinate and antimycin A was reported to inhibit the stimulation of both lipid peroxidation [measured with the thiobarbituric acid reactive substances (TBARS) assay] and protein carbonylation by ADP/Fe^3+^/Asc (314). However, these studies could not determine whether the oxidative damage to proteins and DNA was independent of lipid peroxidation, and thus whether the effect of CoQH_2_ was direct or indirect (240, 313).

#### 3.2.5. CoQ in lipoproteins.

An additional antioxidant role of CoQ in mammals appears to be to protect lipoproteins from oxidation. Lipoproteins are lipid-protein complexes that are synthesized in the liver and secreted into the circulation, where they function as vehicles for lipids. A small amount of CoQ is found in the blood, where it is carried by lipoproteins, mostly in low-density lipoproteins (LDL) and very low-density lipoproteins (VLDL), in which it has been shown to exist predominantly in its reduced form (315–317). Several in vitro investigations into lipoprotein oxidation reported a protective effect of the presence of CoQH_2_. For example, exposure of human plasma or lipoproteins isolated from human subjects to peroxyl radicals [such as 2,2′-azobis(2-amidinopropane) dihydrochloride or 2,2′-azobis(2,4-dimethylvaleronitrile)] led to rapid oxidation of CoQ_10_H_2_ (315, 318). Moreover, CoQ_10_H_2_ was shown to be the first consumed after exposure to such ex vivo oxidation, and the onset of lipoprotein lipid oxidation (measured by HPLC detection of lipid hydroperoxides) corresponded closely with the complete conversion of CoQ_10_H_2_ to CoQ_10_. That is, lipid peroxidation of lipoproteins proceeded rapidly only after most CoQ_10_H_2_ was consumed (318–320). Furthermore, an increase of CoQ_10_ in plasma (≈2- to 7-fold increase) or plasma lipoproteins (≈3- to 4-fold increase) from dietary CoQ_10_ supplementation was demonstrated to enhance the resistance to ex vivo-induced oxidation of lipoproteins (315, 317, 321–323). Thus, it was long proposed that CoQ is crucial for lipoprotein protection against oxidation (318). But whether the action is direct or indirect via regeneration of VE is not clear. The average number of CoQ_10_H_2_ molecules per native LDL was reported to be 0.5–1.0 versus 6–12 for VE (315, 322). Correspondingly, the mean CoQ_10_ level in human serum was reported to be >10 times less than that of VE (316).

In vivo, LDL oxidation is thought to occur mainly in the subendothelial space in the vascular wall and is widely regarded as a key factor in the pathogenesis of atherosclerosis (324). However, the importance of CoQ for preventing LDL oxidation in vivo and thus reducing the risk of atherosclerosis is unknown. It was shown that in apolipoprotein E-deficient mice fed a high-fat diet, which is a widely used murine model for atherosclerosis, supplementation of CoQ_10_ resulted in a variable increase of the total aortic content of CoQ and in reduced aortic LOOH accumulation as well as in a smaller area of atherosclerotic lesions in the aorta (317, 323). This suggests a benefit of CoQ in inhibiting oxidized LDL (ox-LDL)-related pathophysiology. However, several studies also reported a lack of any effect of CoQ_10_ supplementation on arterial lesions or endothelial function, despite decreased aortic lipid oxidation and increased resistance of plasma lipids to ex vivo oxidation (319, 321). CoQ is packaged into LDL and VLDL in the liver, and the liver is one of the few organs that readily takes up oral CoQ_10_ (8, 30, 42, 82, 123, 325). Thus, although it might be possible to significantly boost CoQ_10_ levels in lipoproteins, more research is needed to determine whether CoQ_10_ supplementation can inhibit the development or progression of atherosclerosis in humans.

#### 3.2.6. Summary.

The chemical properties of CoQ and its abundant distribution in membranes where its active antioxidant form can be continuously regenerated by enzymatic mechanisms suggest that CoQ is an important participant in redox control. Moreover, the fact that CoQ is actually in the immediate vicinity of the site of ROS production in the IMM is also thought to contribute to its function as an antioxidant. As we have seen, there is a fair number of early in vitro studies that point to CoQ’s antioxidant bona fides. Perhaps the most direct evidence is provided by its ability to inhibit lipid peroxidation in membrane vesicles when its reduced form is readily regenerated. However, it remains unclear how important the antioxidant properties of CoQ are in vivo, compared to other antioxidant mechanisms, and how exactly it acts. In the extraction and reincorporation studies with artificial membrane systems such as liposomes and SMPs, the amount of CoQ reincorporated was likely not physiological. Moreover, most of the in vitro studies examined the effect of CoQ on lipid peroxidation induced by direct exposure to oxidant compounds. The level of oxidative insults to which tissue cells are exposed under normal conditions is likely many orders of magnitude lower than that produced by such artificial conditions. In summary, although CoQ has an intrinsic free radical-scavenging potential, its ability to regenerate other antioxidants, especially vitamin E, might be of greater importance. Overall, despite the reputation of CoQ as an antioxidant, its action as a prooxidant is actually better documented.

### 3.3. CoQ Level and Oxidative Stress

Generally speaking, oxidative stress is defined as a state in which ROS levels are high enough to lead to a deleterious level of molecular damage. As outlined in the sections above, the capability of CoQ to function as a source of mitochondrial ROS as well as an antioxidant itself is well recognized. However, the relative importance of these two functions in vivo, in different tissues and under different conditions, is yet to be clearly understood. On this front, a better understanding is needed for elucidating the pathophysiology that accompanies CoQ deficiency in patients (see sect. 6), as well as for addressing the rational use of CoQ_10_ as an antioxidant health supplement.

#### 3.3.1. Less CoQ and oxidative stress.

As we have seen, mitochondria are considered to be the major source of ROS in most cell types, and CoQ^•−^, the reactive intermediate of CoQ redox cycling, is one of the sources of mitochondrial ROS (146, 326). An inadequate CoQ concentration in the IMM could mean insufficient CoQ available to carry electrons to CIII. This could have consequences for O_2_^•–^ generation at CI, at CIII, and during electron transfer from other CoQ-dependent hydrogenases (such as DHODH and G3PDH). All this will depend on the type of cells, the sources and usage of respiratory substrates, and the severity of the CoQ shortage (327). Less CoQ redox cycling at CIII likely decreases O_2_^•–^ release at that site. However, a reduction of CoQ-dependent CI + III activity may increase the NADH-to-NAD^+^ ratio in the mitochondrial matrix, leading to the hyperproduction of O_2_^•–^ at the FMN site of CI (see sect. 3.1.1) (166). Moreover, an overall decrease of respiratory chain activity caused by CoQ deficiency may influence mitochondrial ROS production by an effect on membrane potential or induction of some compensative or adaptive alterations of ETC function, such as altered supercomplex structure and mPTP opening. For example, under conditions in which mitochondrial respiration is strongly inhibited, CV (the F_0_F_1_-ATP synthase) can run in reverse, hydrolyzing ATP and extruding protons out of the matrix (328). A study with human SH-SY5Y neuronal cells has suggested that this can result from CoQ deficiency (at an ≈50% decrease in CoQ_10_ level) (329). It is still unknown whether this occurs in other cell types and under milder, or more severe, CoQ deficiency. Finally, as discussed above, CoQ is widely believed to be an endogenous antioxidant, acting directly through its radical-scavenging chemical property and indirectly by regenerating the antioxidant form of VE and VC. Hence, the consequences of CoQ deficiency on the cellular redox state and oxidative damage, determined by the balance between ROS production and clearance, are unlikely to be straightforward and universal.

##### 3.3.1.1. sensitivity to oxidative stress of fully coq-deficient yeast mutants.

In the yeast *S. cerevisiae*, a common feature of fully CoQ-deficient *coq* deletion mutants (including *coq1Δ*, *coq2Δ*, *coq3Δ*, *coq5Δ*, *coq6Δ*, *coq7Δ*, *coq8Δ*, and *coq9Δ*) is hypersensitivity to treatment with the PUFA linolenic acid (αLnn) (330–333). Exogenously supplemented αLnn can readily incorporate into the yeast cell membrane, and autoxidation of linolenic acid gives rise to LOOH and many secondary products, some of which are toxic, causing cell death (334, 335). All the *coq* mutants lacking CoQ display lower survival after exposure to PUFAs, which are known to undergo an autocatalytic oxidation process. In contrast, they are not affected by monounsaturated oleic acid that does not autooxidize easily and is much less vulnerable to peroxidation (331–333). In contrast, the *cor1* and *atp2* mutants, with defects in CIII and CV, respectively, remain resistant to PUFA treatment (331). Consistently, quantification of lipid peroxides and late-stage aldehyde products (TBARS) as markers of lipid peroxidation showed higher levels in αLnn-treated *coq3Δ* yeast compared to the wild type (332). These findings indicate a protective role of CoQ against the toxicity of PUFA oxidation, which is further supported by the observation that the sensitivity of *coq3Δ* yeast to αLnn can be reduced by vitamin E and the antioxidant butylated hydroxytoluene (BHT) (331, 332). These studies, however, did not examine the effect of exogenous CoQ on the lipid peroxidation sensitivity of the mutants. In addition, surprisingly, vitamin C showed no effect on αLnn sensitivity (331). Likely, the lipid peroxidation stress imposed by exogenous supplementation of αLnn is much higher than what the cells normally encounter. Thus, these studies cannot establish unambiguously the importance of CoQ in membrane protection under normal physiological conditions. The yeast *coq1*-null mutant (*coq1Δ*) was shown to exhibit enhanced sensitivity to H_2_O_2_ killing and elevated O_2_^•–^ levels compared to wild-type yeast (330). However, a similar hypersensitivity to H_2_O_2_ was also reported for the mutants with deletions of subunits of mitochondrial CIII (*cor1Δ*, *cyt1Δ*), suggesting that the elevated O_2_^•–^ and the sensitivity to H_2_O_2_ are more likely to be attributable to a consequence of respiratory dysfunction rather than a lack of the antioxidant role of CoQ (330). Finally, the *coq10Δ* yeast mutant, which has a near wild-type content of CoQ_6_ in the stationary phase but inefficient CoQ_6_ biosynthesis during the log phase (≈30% relative to wild type), was also found to be sensitive to αLnn exposure during mid-log phase (though less than *coq3Δ* mutant), and, consistently, αLnn-treated *coq10Δ* mutants showed significantly increased lipid peroxidation levels compared to the wild type (331). Whether this implies insufficient CoQ_6_ biosynthesis in the *coq10* mutant for protection against PUFA stress or whether there is a requirement for Coq10 for the antioxidant function of CoQ remains to be resolved.

##### 3.3.1.2. coq deficiency and oxidative stress in mammalian cells and tissues.

**FIGURE 12.**
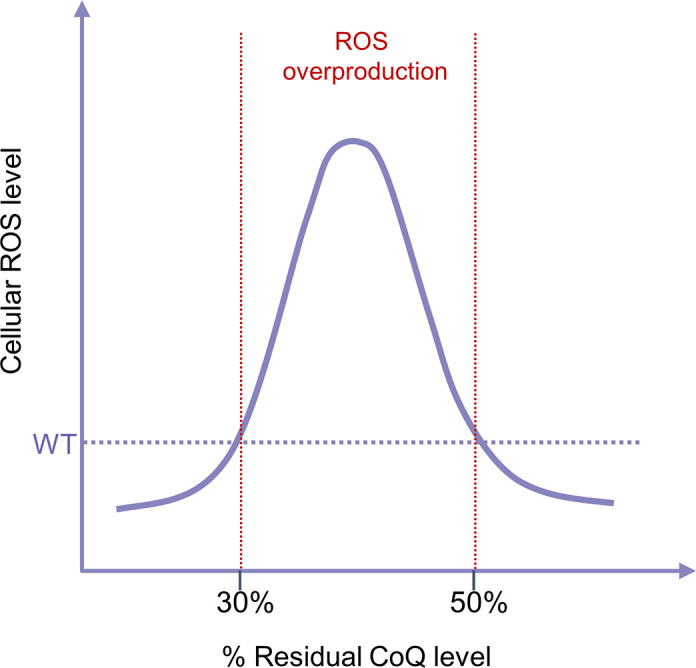
Relationship between the levels of CoQ and ROS in human skin fibroblasts. Severe (<30% of residual CoQ_10_) and moderate (>50% of residual CoQ_10_) defects are not associated with induction of oxidative stress. However, an intermediate defect (30–50% of residual CoQ_10_) results in an increase of cellular ROS levels. WT, wild type. See glossary for other abbreviations.

Using cultured skin fibroblasts from patients that carry genetic lesions in the CoQ biosynthetic pathway, Quinzii et al. were the first to assess the consequences of different CoQ deficiency levels on the cells’ ROS level and oxidative stress status. They found that in *COQ2* mutant fibroblasts that have 30–40% normal level of CoQ there were signs of excess ROS, as measured by MitoSOX (a mitochondrial fluorescent probe with high reactivity to O_2_^•–^) and lipid peroxidation markers (MDA and 4-HNE), but the markers of oxidative stress were absent in the patient cells with 12–18% (due to *PDSS2* or *COQ9* mutation) or >50% (due to *ADCK3* mutations) of CoQ_10_ (113, 336). See sect. 5.1 on biosynthesis for more information about the function of these genes. A similar finding was reported for CoQ_10_ deficiency induced by inhibition of CoQ synthesis with 4-nitrobenzoic acid (4-NB, a competitive inhibitor of COQ2). Lowering the CoQ_10_ level to 40–50% (in controls and *ADCK3* mutant fibroblasts) led to an increase in MitoSOX fluorescence (336). A later study also showed a higher ROS level (measured with both 2′-7′-dichlorodihydrofluorescein diacetate (DCFH-DA) and MitoSOX] in *ADCK3* mutant fibroblasts with ≈50% CoQ_10_, compared to nonmutant controls (337). And the measures of increased ROS levels were associated with increased sensitivity to H_2_O_2_ as well as elevated levels of lipid peroxidation and protein tyrosine nitration, a marker of nitro-oxidative damage (337). Taken together, an inverted U-shaped relationship is postulated for levels of CoQ and oxidative stress: an increase of oxidative stress is only manifested under moderate CoQ_10_ deficiency (30–50% residual CoQ_10_), whereas a more severe or milder deficit of CoQ_10_ (<30%, or >50% of normal level) is not accompanied by overproduction of ROS (FIGURE 12) (113, 336). Such a relationship might be due to the dual pro- and antioxidant properties of CoQ.

Whether such a relationship can be expected in other cell types has not been systematically investigated. Induction of CoQ deficiency in wild-type cells relies on pharmacological inhibition of CoQ biosynthesis with compounds that interfere with the normal CoQ biosynthesis pathway. Most of the studies use reagents such as *para*-aminobenzoic acid (pABA) and 4-NB, which are competitive inhibitors of the COQ2 enzyme. With such an approach, it can be challenging to produce different CoQ deficiency levels or a very severe loss in CoQ without negatively affecting cell viability. In mouse adipocyte 3T3-L1 cells, it was shown that an ≈25–50% decrease of mitochondrial CoQ_9_, due to treatment with 4-NB or knockdown of the CoQ biosynthetic gene *Coq7* or *Coq9*, led to increased mitochondrial peroxiredoxin (PRDX) dimerization, which was used as an indicator of H_2_O_2_ burden, as well as increased concentration of mito-2-hydroxyethidium, an O_2_^•–^-specific oxidation product of MitoSOX (338). The effect of CoQ deficiency at levels outside of this range was not investigated. In pABA-treated human myeloid leukemia HL-60 cells, ≈50% decrease in the level of cellular CoQ_10_ was also shown to display, in addition to a decrease of CoQ-dependent respiratory activity, a higher level of O_2_^•–^ compared to a no-treatment control (339). Moreover, a similar level of reduction of CoQ_10_ levels in 4-NB-treated T67 human glioma cells (by ≈50%) was demonstrated to cause higher cellular and mitochondrial oxidative stress (demonstrated by increased DCFH-DA and MitoSOX signals) as well as an elevation of nitric oxide (NO^•^) levels (338). In both cell lines, treatment with exogenous CoQ_10_ reversed the levels of oxidative stress markers to control values, supporting a causal link between the CoQ decrease and the higher ROS reporter intensity (120, 339). In human neuronal SH-SY5Y cells, increased mitochondrial oxidative stress (measured by MitoSOX probe), but no sign of increased lipid peroxidation (visualized by C11-BODIPY staining), was observed at both 46% and 76% residual CoQ_10_ levels produced by treatment with pABA. This type of cell appears to display higher ROS production at slightly higher CoQ levels than skin fibroblast. Conceivably, endogenous CoQ levels and the contributions of CoQ to ROS production and cellular antioxidant status differ in different cell types and under different conditions. It should be noted that so far all the in vitro studies with CoQ-deficient or supplemented cells were conducted under standard cell culture conditions where the O_2_ levels are hyperoxic with respect to the 1–6% experienced by most mammalian cells in vivo (340). However, the level of oxygen that cells are exposed to is known to be able to impact biochemical reactions and cellular activities, especially energy metabolism and ROS production. It is possible therefore that O_2_ levels affect experimental outcomes and the conclusions drawn from them (340–342). Thus, the potential implications of in vitro findings for in vivo conditions remain to be evaluated. It is also not known how intracellular O_2_ levels could affect CoQ levels. Interestingly, a recent study showed that culturing human endothelial cells under hypoxia (1% O_2_) leads to a marked decrease in CoQ_10_ content (343).

With isolated mouse heart mitochondria, an ≈90% decrease in CoQ levels has been shown to cause a significant reduction in ROS production rate. The mitochondria used in the study were isolated from the heart of inducible *Coq7* knockout (KO) mice (123). The hearts were harvested from ≈8-mo-old mice, ≈6 mo after the gene was excised. Mitochondrial ROS production rate was assessed by the commonly used Amplex Red/HRP assay. A decrease of net O_2_^•−^ release was seen with both CI and CII substrates in the presence of the CI CoQ site inhibitor rotenone or the Q_i_ inhibitor antimycin A, suggesting lower ROS production at both CI and CIII and in both forward and reverse electron transfer modes (123). Moreover, it was shown that despite a global deficit of CoQ in the mutant mice (here an average of 10–30% CoQ left), no sign of systemic oxidative stress was detected, as indicated by normal levels of plasma F_2_-isoprostanes (a result of lipid peroxidation) and plasma content of 8-OHdG (an oxidative DNA damage marker) (123). An increase in the expression of antioxidant enzymes often occurs in response to elevated oxidative stress. However, in the kidneys and heart of the *Coq7* KO mice that contain only 10–15% of CoQ relative to that in non-KO control mice, a lower level of the cytoplasmic superoxide dismutase 1 (SOD1) was detected, as well as a markedly decreased level of catalase in the kidney (123). These are likely to be compensatory responses that may be induced by low cytoplasmic ROS and that help maintain ROS at physiological levels necessary for optimal cell function. Thus, overall, a very low level of CoQ in these mice appears to be associated with a lower oxidative stress state, most likely because of reduced mitochondrial ROS production. It is noteworthy, however, that *Coq7* mutants accumulate the biosynthetic intermediate DMQ, which might have phenotypic consequences on ROS metabolism.

The effect of CoQ deficiency on oxidative stress has also been examined in the kidney of *Pdss2^kd/kd^* mice harboring a homozygous kidney disease (*kd*) mutation in *Pdss2*. The partial loss-of-function mutation of *Pdss2* was identified as a spontaneous mutation in a colony of inbred mice and was designated *kd* because renal failure is the most prominent disease manifestation in mutant *kd/kd* homozygotes (30, 344–346). *Pdss2^kd/kd^* kidney displayed an increase of dihydroethidine fluorescence, indicating elevated ROS production, and this is associated with 20–30% residual CoQ_9_ content (124). In contrast, levels of fluorescence comparable to the wild type were not observed in other tissues of the mutant (brain and muscle), despite similar reductions in CoQ_9_ levels (124). Moreover, two separate markers of oxidative stress, anti-nitrotyrosine and anti-4-HNE immunostaining, were also found to be elevated only in the glomerulus of *Pdss2^kd/kd^* mice where tissue damage occurs (124). Lower CoQ-dependent ETC complex activities (CI-CIII and CII-CIII) were noted in *Pdss2^kd/kd^* kidney but also in other tissues (124). Taken together, it has been postulated that in the kidney (in particular in the glomeruli) CoQ deficiency is more liable to lead to high oxidative stress than in other tissues. This would explain why *Pdss2^kd/kd^* mutants mainly develop a renal phenotype, despite widespread CoQ_9_ deficiency (30, 124). A beneficial effect in preventing renal lesions in these mice of treatment with CoQ_10_ or with probucol, a hypolipidemic agent with antioxidant action, lends further support to this idea (347, 348). This is in stark contrast to findings in other mouse mutants of CoQ biosynthetic genes. No sign of oxidative damage in the kidney (measured as 8-OHdG levels) and normal appearance of kidney glomeruli and tubules were found in inducible *Coq7* knockout mice at 6 mo of age, despite a severe depletion of total CoQ (≈10% of residual CoQ levels). And no renal dysfunction (as measured by blood urea nitrogen) was observed in *Coq7* knockout mice treated with 2,4-dihydroxybenzoic acid (2,4-DHB), which showed ≈ a 30% residual CoQ level in the kidney (see sect. 5.4 for additional details about 2,4-DHB) (123). A study with a *Coq9* mutant mouse (*Coq9^R239X^*) reported that despite a severe loss of total CoQ (<30% of the normal level) across tissues and fatal mitochondrial encephalomyopathy, the phenotype showed no renal involvement (349). This study also reported increased 8-OHdG and 4-HNE staining in the brain, suggesting elevated oxidative stress, but the possible role of ROS in the brain pathology observed has not yet been elucidated (349). The reasons for these phenotypic discrepancies are not understood. A more recent study showed that an increase of the lipid peroxidation product MDA was associated with ≈25% residual CoQ levels (CoQ_9_ + CoQ_10_) in a brown adipose tissue (BAT)-specific in vivo model of CoQ deficiency obtained by targeting *Pdss2* (119). Overall, it appears that in certain situations, but not all, CoQ deficiency causes elevated ROS generation and increased oxidative damage. Why this is and its role in CoQ deficiency-associated pathophysiology are not yet clear.

### 3.4. Utility of Exogenous CoQ_10_ Supplementation

#### 3.4.1. In vitro and in vivo CoQ_10_ supplementation.

For in vitro supplementation, CoQ that has been dissolved in an organic solvent such as ethanol is often directly added to culture media. When added in this manner it can be taken up efficiently by cells, after which it can reach mitochondria and most likely other cellular compartments as well (41, 42, 82, 110, 350–353). In fact, a dramatic increase of CoQ_10_ level is often observed in cells after culture in the presence of CoQ_10_ (TABLE 1). The respiration deficiency of CoQ-deficient cells can be rescued by exposure to CoQ, demonstrating the ability of exogenous CoQ to reach the mitochondrial inner membrane (42, 82, 115, 351, 358–360). The cellular mechanisms whereby CoQ is taken up from the culture medium and transported to various locations are not well known. In mammalian cells, the class B scavenger receptor CD36 and Niemann–Pick C1-like 1 (NPC1L1, an intestinal cholesterol transporter) have been shown to be involved in exogenous CoQ uptake (42, 356, 361). In yeast, several genes have been identified that affect CoQ uptake and intracellular trafficking. The products of most of these genes are associated with endosomal trafficking, but their exact roles are yet to be understood (352, 360, 362).

**Table 1.** Reported effects of in vitro CoQ_10_ supplementation on cellular CoQ_10_ levels

Cell Type	Total Dosage	Treatment Effects		Refs.
		On CoQ_10_ levels	On ETC function	
*Cells with intact CoQ biosynthesis*				
SH-SY5Y neuroblastoma cells	5 µM 3 days	↑↑↑ (cells)	ND	(354)
Human astrocytoma cells & rat embryonic cardiomyocytes	10 µM 1 day	↑↑↑ (cells)	NE	(41)
Breast cancer cell lines	9 µM 2 day	↑↑↑ (mito)	NE	(355)
Mouse brown adipocytes	10 µM 3 h	↑↑↑ (cells)	NE	(356)
Human dermal fibroblasts	5 µM 1 day	↑↑↑ (cells)	NE	(351)
Mouse embryonic fibroblasts	2.5 µM 3 days	↑↑ (cells)	ND	(42)
*Cells with defective CoQ biosynthesis*				
Human dermal fibroblasts	5–10 µM 1–7 days	ND	ATP ↑, respiration↑, CI-CIII ↑, CII+CIII ↑	(80, 115, 351, 357)
Mouse embryonic fibroblasts	2.5–10 µM 3–4 days	↑↑ (cells)↑↑ (mito)	Respiration↑	(42, 83)

Cell treatment with CoQ_10_ was carried out in the listed studies by adding CoQ_10_ dissolved in a solvent (such as ethanol) to the culture media. CI-CIII, complex I-III activity, CII-CIII, complex II-III activity; ETC, electron transport chain; mito, mitochondria; ND, not determined; NE, no effect. ↑, <2-fold increase; ↑↑, 2- to 10-fold increase; ↑↑↑,  ≥10-fold increase.

To investigate the effects of CoQ supplementation in vivo, the most commonly used route is oral. Specifically, CoQ_10_ supplementation in food or water has been used in most rodent studies, as the human isoform can be easily distinguished from the rodents’ predominant endogenous isoform (CoQ_9_). Although direct experimental evidence is still lacking, it is generally believed that, like other dietary lipids, orally administered CoQ_10_ is absorbed into the intestinal enterocytes. In the enterocytes, CoQ_10_ is packaged into chylomicrons that, once formed, are released from the enterocytes into the lymphatic system, and eventually into the bloodstream (363, 364). CoQ_10_ can go back into the circulation after liver uptake of chylomicron remnants and assembly and secretion of CoQ_10_-containing lipoproteins (363, 364). However, because of its high hydrophobicity and large molecular weight, the gastrointestinal absorption of CoQ_10_ is known to be slow and poor, resulting in low bioavailability (364, 365). In fact, animal studies suggest that typically <5% of orally administered CoQ_10_ can be absorbed and reach the bloodstream (366). TABLE 2 lists the reported effects of CoQ_10_ supplementation in mice on tissue CoQ levels and ETC function. Consistent with the role of the liver in the metabolism of oral CoQ, it is in the liver that a significant response is most often observed. For other tissues, much more variable responses have been reported. Finally, it is worth noting that, to overcome the low oral bioavailability of CoQ_10_ and the disappointing outcomes of patient treatment with CoQ_10_ (40, 370–372), novel CoQ_10_ formulations have been proposed. Here are a few examples: CoQ_10_ nano-liposomes, lipid-CoQ_10_ conjugate nanodispersion (BPM31510), multicomposite CoQ_10_ terclatrate (Q-TER), CoQ_10_/β-cyclodextrin complexes, and nano-micellar CoQ_10_ formulations (Ubisol-Q_10_ and micellular formation with caspofungin) (42, 373–378). These formulations were demonstrated to have higher delivery efficiency in vitro compared to the addition of free CoQ_10_ to the culture medium, and some of them have already been shown to deliver better bioavailability of CoQ_10_ in vivo (41, 42, 365, 377–380). Administration via alternative routes, especially intravenous, which is possible with some formulations, is particularly appealing with respect to overcoming the poor and variable absorption of oral CoQ_10_ (42, 381, 382). However, the effects of these treatments on tissue CoQ levels and on in vivo efficacy in relieving deficiency and deficiency symptoms remain to be determined.

**Table 2.** Reported effects of CoQ_10_ supplementation on tissue CoQ_10_ levels

Model Type	CoQ_10_ Formulation	Total Dosage	Treatment Effects		Refs.
			On CoQ_10_ Levels	On ETC Function	
*Oral feeding*					
Wild-type mice					
	CoQ_10_ in chow	0.5% CoQ_10_, 3 mo	Kidney ↑, liver ↑↑, brain ↔	ND	(90)
	CoQ_10_ in chow	93 or 371 mg/kg body wt/day, 3.5 or 17.5 mo	Heart ↑, liver ↑↑, kidney ↑↑, skeletal muscle ↑↑, brain ↔, mito (liver, heart, kidney, skeletal muscle) ↑	Respiration of liver mito. ↔	(367)
	Liposomal CoQ_10_ in drinking water	∼300–400 mg/kg body wt/day, 3–7 mo	Liver ↑↑↑, muscle ↔, heart ↔, kidney ↔	NE	(82, 123)
	CoQ_10_ in chow	2.81 mg/g CoQ_10_ in food, 3 wk	Liver and liver mito. ↑↑↑	NE	(8)
	Liposomal CoQ_10_ in drinking water	0.4 mg/mL, 12–13 wk	Liver ↑↑↑; ovaries ↑↑, kidney ↔	oocyte mito. respiration and ATP ↑	(325)
	Liquid CoQ_10_	15 mg/kg/day for 3 mo	Liver ↑↑; muscle ↔,	ND	(117)
CoQ deficiency mouse models					
* Pdss2^kd/kd^*	CoQ_10_ in chow	0.5% CoQ_10_ in food, 3 mo	Kidney ↑, liver ↑↑, brain ↔	Kidney CI-III and CI-III ↔	(90)
* Pdss2^kd/kd^*	Liposomal CoQ_10_ in drinking water	∼200 mg/kg body wt/day, 3.5 mo	Kidney ↔	ND	(347)
* Coq7* KO	Liposomal CoQ_10_ in drinking water	∼300–400 mg/kg body wt/day, 3–7 mo	Liver ↑↑↑, muscle ↔, heart ↔, kidney ↔	Respiration of liver mito. ↑	(82, 123)
* Coq7^+/^*	CoQ_10_ in chow	2.81 mg/g CoQ_10_ in food, 3 wk	Liver and mito ↑↑↑	Liver CI-CIII ↑	(8)
* Coq9^R239X^*	Liquid CoQ_10_	240 mg/kg body wt/day, 2 mo	Liver ↑↑, muscle ↑↑, heart ↔, kidney ↔, brain ↔, kidney ↔	Cerebrum mito. CI-CIII ↔, CII-CIII ↔	(368)
* Coq9^R239X^*	Liquid CoQ_10_H_2_	240 mg/kg body wt/day, 2 mo	Liver ↑↑, kidney ↑, muscle ↑↑, heart ↑, kidney ↑, brain ↑, cerebrum mito. ↑	Cerebrum mito. CI-CIII ↑ CII-CIII ↑	(89, 368)
* ADCK2^+/−^*	Liquid CoQ_10_	15 mg/kg/day for 3 mo	Liver ↑↑; muscle ↑	ND	(117)
*Intraperitoneal injection*					
Wild-type mice	Intralipid-solubilized CoQ_10_	200 µL of 1.45 mM once	Liver ↑↑↑, BAT ↑↑, skeletal muscle ↔, serum ↔	NE	(356)
	CoQ_10_ in 10% Tween 20	0.1 mg/g/day for 3 days	Heart mito. ↔	NE	(369)
*Intravenous administration*					
Wild-type mice	Micellar CoQ_10_ solution prepared with caspofungin	12.0 mg/kg body wt/day, 10 days	Plasma ↑↑↑, liver↑↑↑, heart ↑↑, muscle ↑↑, kidney↑, spleen↑↑, lung ↑↑, brain ↑, heart mito ↑	ND	(42)

BAT, brown adipose tissue; CI-CIII, complex I-III activity; CII-CIII, complex II-III activity; ETC, electron transport chain; mito., mitochondria; ND, not determined; NE, no effect. ↑<2-fold increase of CoQ_10_ levels; ↑↑2- to 10-fold increase of CoQ_10_ levels; ↑↑↑≥10-fold increase of CoQ_10_ levels; ↔no change in CoQ_10_ levels.

#### 3.4.2. Physiological effects of added exogenous CoQ.

As stated in sect. 2.3, boosting CoQ levels in cells without CoQ deficiency is not associated with increased respiratory rate, indicating that the normal CoQ amount is sufficient for mitochondrial respiratory function. On the other hand, increasing CoQ content may potentially augment antioxidant protection against oxidative stress-related damage. Many studies with cultured cells describe cytoprotective effects against oxidative stress of treatment with exogenous CoQ_10_. For example, in human skin fibroblasts, human umbilical vein endothelial cells (HUVECs), and rat pheochromocytoma (PC12) cells, treatment with CoQ_10_ ameliorates H_2_O_2_-induced cytotoxicity, suppresses senescent phenotypes induced by H_2_O_2_, or attenuates the increase of DCFH-DA fluorescence and MDA formation after H_2_O_2_ stress (383–385). In human neuroblastoma SH-SY5Y cells, protective effects of CoQ_10_ pretreatment were described for the toxicity of paraquat (1,1′-dimethyl-4,4′-bipyridinium), a redox cycler widely used to stimulate O_2_^•−^ production in mitochondria (386). However, where exactly the exogenous CoQ_10_ intervenes in the cytotoxic actions of H_2_O_2_ or paraquat is not clear. Another example is a finding with isolated rat hepatocytes. Here it was shown that CoQ_10_ prevented cytotoxicity induced by CI inhibition by rotenone but not by the CIII inhibitor antimycin A and that the cytoprotection against rotenone was abolished after preincubation of the cells with dicumarol to inhibit DT-diaphorase (NQO1) (387). As expected, both rotenone and antimycin A treatments increase overall ROS levels as measured by DCFH-DA (387). This finding suggested that NQO1 may have a major role in maintaining CoQH_2_ levels in the cell. Note that reduction of CoQ by NQO1 oxidizes NADPH, which could alter the cellular NAD(P)^+^/NAD(P)H redox balance, thus affecting many processes.

Because of its extreme hydrophobicity, effective treatment with CoQ_10_ for whole animals has been challenging. In short, CoQ_10_ can only be given orally, but the oral bioavailability of CoQ_10_ is insufficient to ensure uptake in most organs. Hepatic uptake of oral CoQ_10_, however, can be substantial (8, 123, 388, 389). For these reasons, here we only take note of some studies that examined oxidative stress-related pathologies induced in the liver and the effect of CoQ_10_ supplementation on them. One of the earliest studies showed that injection of bacterial endotoxin (lipopolysaccharide) induces liver damage in mice and that this is associated with elevated liver lipid peroxidation. A drop in the levels of CoQ_9_H_2_ and vitamin E was also observed, presumably because of their oxidation. Administration of CoQ_10_ together with the endotoxin was shown to suppress lipid peroxidation, prevent the decrease in CoQ_9_H_2_ and vitamin E, and increase the survival rate of treated mice compared to endotoxin-only control mice (390). Oral CoQ_10_ supplementation in mice or rats was also shown to inhibit hepatic lipid peroxidation and liver toxicity induced by chemical exposure, such as to carbon tetrachloride, acetaminophen, or valproic acid (391–393). On the other hand, although the levels of lipid hydroperoxides were shown to increase significantly in the liver of aged PUFA-fed rats, this was not improved by CoQ_10_ supplementation (389). Thus, whether CoQ_10_ has a protective effect on liver lipid peroxidation remains ambiguous. Exploring the effects of exogenous CoQ on the redox status in other tissues will only become feasible when new methods to boost CoQ levels throughout the animals will become widely used (42).

## 4. OTHER FUNCTIONS OF CoQ

In this section, we focus on a number of CoQ functions that are less known and characterized compared to transporting electrons in the inner membrane of mitochondria and roles in ROS metabolism.

### 4.1. Role in Protection from Ferroptosis

The plasma membrane redox system (PMRS) was discovered in 1960 (245). CoQ at the plasma membrane is a vital part of the PMRS (see sect. 3.2.1). The ferroptosis suppressor protein 1 (FSP1, also known as AIFM2) was recently identified as a new member of the CoQ oxidoreductases that can regenerate CoQH_2_ in the plasma membrane. In this role, FSP1 can inhibit ferroptosis, a distinct form of regulated cell death that is characterized by excessive iron-dependent oxidation of PUFAs in cell membranes (252, 253, 256). Although many details of ferroptosis are yet to be worked out, this special form of cell death has been implicated in various pathophysiological conditions and diseases (394). Oxidation of PUFA during oxidative stress generates LOOH, which is considered a pivotal trigger of ferroptosis. The excessive accumulation of LOOH can lead to an unusual increase in the tension and cation permeability of the membrane, ultimately altering the ion content of the cell and rupturing the membrane (395).

**FIGURE 13.**
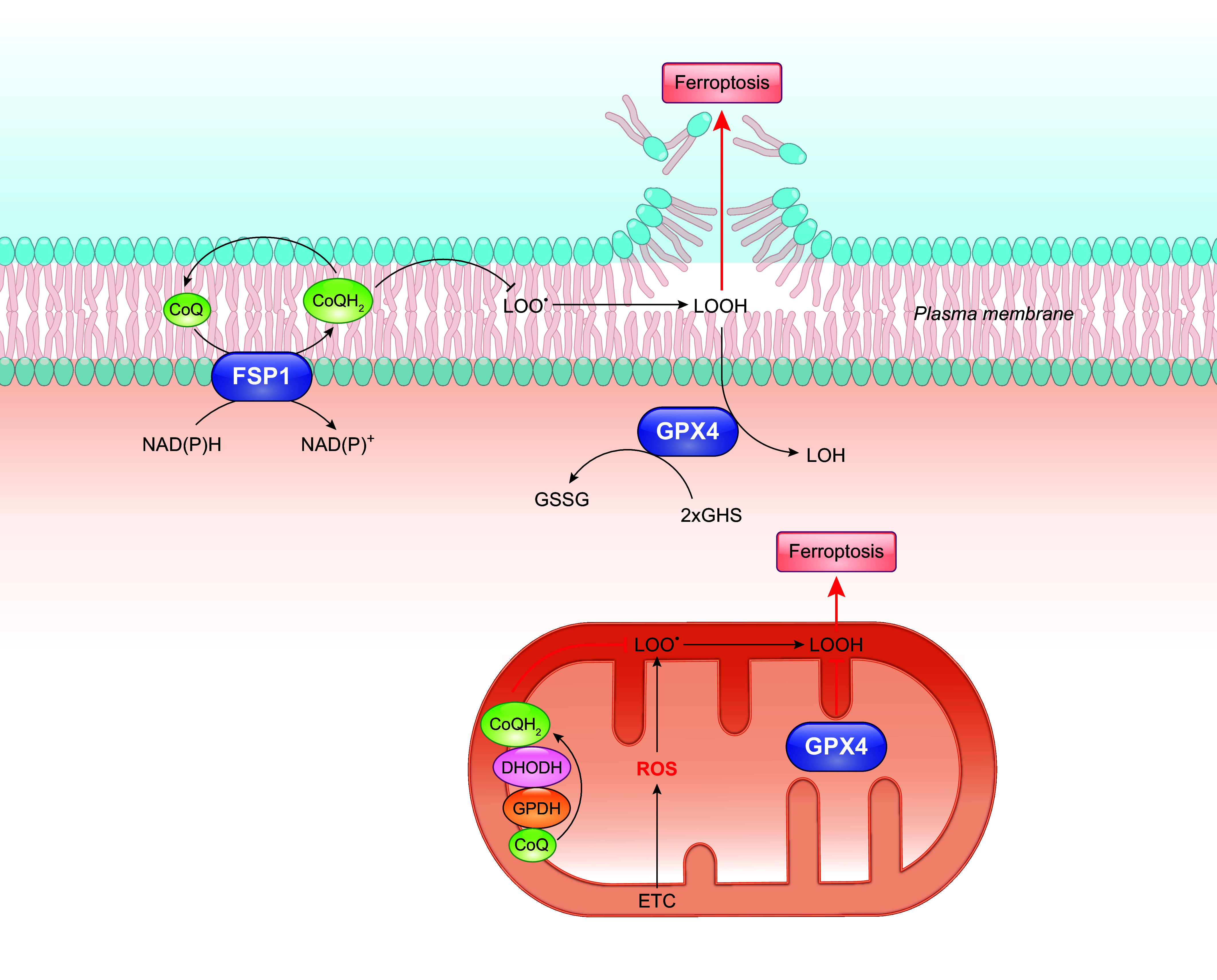
Role of CoQ in ferroptosis regulation. Ferroptosis suppressor protein 1 (FSP1) acts as an oxidoreductase mainly localized on the plasma membrane and reduces CoQ to CoQH_2_ using electrons from reduced nicotinamide adenine dinucleotide (NADH) or reduced nicotinamide adenine dinucleotide phosphate (NADPH). By directly scavenging lipid peroxy radicals (LOO^•^) generated from lipid peroxidation, the fully reduced form of CoQ, CoQH_2_, prevents excessive lipid peroxidation and thus inhibits ferroptosis. In the cytosol and mitochondria, glutathione peroxidase 4 (GPX4) converts glutathione (GSH) to oxidized glutathione (GSSG) while reducing lipid hydroperoxides (LOOH) to lipid alcohols (LOH), which is the main mechanism to regulate ferroptosis. Mitochondrial ROS production likely contributes to ferroptosis. On the other hand, CoQH_2_ generation from respiration and other dehydrogenases such as dihydroorotate dehydrogenase (DHODH) and glycerol-3-phosphate dehydrogenase (GPDH) likely enhance defense against ferroptosis by inhibiting lipid peroxidation. See glossary for other abbreviations.

Glutathione peroxidase 4 (GPX4), which is present in both the cytosol and mitochondria, is arguably the most important enzyme that keeps ferroptosis in check (394). The FSP1/CoQ pathway provides another distinct defense mechanism against ferroptosis at the plasma membrane (252, 256) (FIGURE 13). FSP1 reduces CoQ to CoQH_2_, using electrons from NAD(P)H (252, 256). CoQH_2_ can scavenge the lipid peroxyl radical (LOO^•^) generated during membrane PUFA oxidation and thus interrupt the spread of the lipid-radical chain reactions (see sect. 3.2.3). The G156A mutation in FSP1, which does not affect FSP1 expression or localization but impairs FSP1-mediated reduction of CoQ_10_, was shown to abolish the antiferroptotic activity of the protein (252). FSEN1, a small-molecule inhibitor of FSP1 that targets FSP1 CoQ oxidoreductase activity specifically, sensitized cancer cells to ferroptotic death induced by loss of GPX4 activity (396). Also, lowering CoQ_10_ levels was shown to hinder the ability of reexpression of FSP1 to rescue the GPX4 inhibitor RSL3-induced ferroptotic death of FSP1 knockout cells (252, 256). These cell culture findings were further corroborated by the in vitro finding that it is only in combination with CoQ_10_ that FSP1 significantly delays the autoxidation of the polyunsaturated lipids of chicken egg phosphatidylcholine liposomes (256).

Superoxide generated from the ETC in the mitochondrial matrix can be converted to H_2_O_2_ by SOD2. H_2_O_2_ subsequently can be converted to hydroxyl radicals (^•^OH) by the Fenton reaction. ^•^OH is one of the most reactive species known and can rapidly abstract hydrogen atoms from PUFA, triggering lipid peroxidation. Therefore, mitochondrial ROS production likely contributes to ferroptosis (397). On the other hand, CoQH_2_ generation from the ETC should enhance protection against ferroptosis by inhibiting lipid peroxidation (398, 399). Reduction in the level or activity of dihydroorotate dehydrogenase (DHODH) or mitochondrial glycerol-3-phosphate dehydrogenase (mGPDH, also referred to as GPD2) decreases the CoQH_2_-to-CoQ ratio, as expected, and this was shown to be associated with increased sensitivity to induction of ferroptosis by inhibition of GPX4, indicating an important role of these two IMM enzymes in the regulation of ferroptosis via the production of CoQH_2_ (398, 399). In addition, treatment with MitoQH_2_ (a mitochondria-targeted analog of CoQH_2_) attenuated the sensitizing phenotype resulting from the knockout of DHODH or mGPDH (398, 399).

The role of CoQH_2_ in ferroptosis is further supported by the observed sensitization to GPX4 inhibitors in cells with impaired CoQ_10_ biosynthesis (due to pharmacological inhibition of COQ2) or overexpression of the alternative oxidase (AOX), which oxidizes CoQH_2_ (252, 398, 400). On the other hand, how boosting CoQH_2_ levels affects ferroptosis is unclear. Overexpression of FSP1 was shown to inhibit ferroptosis and improve the loss of cell viability induced by RSL3 in H1703 and H446 lung cancer cells that have relatively low levels of FSP1 (252). A ferroptosis-inhibiting effect was also reported by overexpressing mGPDH (401). However, a direct link between the inhibition of ferroptosis and the level of reduction of CoQ remains to be clarified. MitoQH_2_ appears to protect against RSL3-induced cell death in HeLa and RPMI7951 (melanoma) cells, but only very little protection was observed in HT-1080 fibrosarcoma cells (252, 399). Idebenone, a short acyl side chain soluble CoQ_10_ analog, was shown to inhibit RSL3-induced ferroptosis in U2OS osteosarcoma cells [Bersuker et al. (252)]. Given potential variations in the integration of different ferroptosis defense mechanisms among cell types as well as variations in redox metabolism, it seems that the range of CoQ levels required for optimum defense against ferroptosis could be quite different among cell types and with different ferroptosis inducers.

### 4.2. CoQ in the Regulation of the Mitochondrial Permeability Transition Pore

The mitochondrial permeability transition pore (mPTP, also referred to as PTP or MTP) is a voltage-gated, nonselective, large IMM conductance channel. The IMM has a low permeability to ions and solutes in order to maintain the proton electrochemical gradient generated during electron transfer to drive ATP synthesis. The energy stored in the form of the transmembrane proton gradient is also used for other mitochondrial processes, such as ion homeostasis, protein import, etc. mPTP opening allows for all low-molecular weight molecules to equilibrate across the IMM, including H^+^. Transient mPTP opening is implicated in ROS signaling and mitochondrial Ca^2+^ homeostasis, whereas sustained opening can cause a collapse of the mitochondrial membrane potential (ΔΨm), uncoupling of OXPHOS, Ca^2+^ release into the cytosol, and eventually rupture of the outer membrane and cell death (402–404).

The mitochondrial membrane potential (ΔΨm) maintained by the H^+^ gradient across the IMM is intrinsically related to ROS generation from the respiratory chain. Indeed, high ΔΨm promotes ROS generation, whereas ROS production tends to decrease at lower ΔΨm (327, 405). It has been proposed that when ΔΨm is high some transients of the respiratory chain electron transport, capable of reducing O_2_ to O_2_^•−^, such as CoQ^•^**^−^**, become long-lived, thereby increasing the possibility of electron leak and ROS production (405). On the other hand, increased mitochondrial ROS can activate mPTP opening, which can lead to further ROS production and release (326). Thus, potentially, CoQ deficiency could induce mPTP opening through increased production of ROS (see sect. 3.3.1). However, this has not been supported by evidence so far. mPTP opening was found not to be significantly altered in the podocytes of mouse *Pdss2* mutants whose CoQ content is only ≈30% that of control mice (406–409).

On the other hand, studies with isolated mitochondria have documented that some CoQ analogs can directly act on mPTP opening. For example, the addition of CoQ_2_, CoQ_10,_ or decyl-ubiquinone (a CoQ derivative with an n-alkyl side chain) to rat liver mitochondria was shown to increase the capacity to retain calcium, suggesting an inhibition of the mPTP (410, 411). In contrast, in isolated rabbit heart mitochondria, the addition of CoQ_2_ was found to stimulate mPTP opening (411). In newborn rat cardiomyocyte mitochondria, the addition of decyl-ubiquinone caused an increase in respiration and a concomitant decline in membrane potential, with both effects being inhibited by Cyclosporine A (CsA) (412).

### 4.3. CoQ in the Regulation of Uncoupling Proteins

Uncoupling proteins (UCPs) are mitochondrial inner membrane proteins that function as H^+^ leak channels. By allowing H^+^ to flow back from the mitochondrial IMS to the matrix, UCPs catalyze the dissipation of the proton electrochemical gradient built up by the respiratory chain. Thus, one key consequence of UCP activation is the uncoupling of mitochondrial respiration from ATP synthesis, leading to energy dissipation as heat (413). In fact, the first UCP protein discovered, UCP1, is expressed mainly in brown adipose tissue (BAT), where it plays a well-defined role in nonshivering thermogenesis (414). The roles of other UCPs that are found throughout mammalian tissues (UCP2 in a wide variety of tissues, UCP3 predominantly in skeletal muscle, UCP4 and UCP5 in the brain) are still largely tentative, such as being possibly important in the immune response, in insulin secretion, and in the control of mitochondrial ROS generation (413, 415–418).

UCP-mediated uncoupling can be activated by free fatty acids (FFA) by a mechanism that is not fully elucidated (419). FFA-induced UCP activity is sensitive to purine nucleotides (PNs), which were first recognized as inhibitors of UCPs (420). In fat tissue, PN-dependent inhibition of respiratory rate accompanied by the restoration of ΔΨm has been considered diagnostic of UCP function (421). However, the PN sensitivity has been mostly demonstrated in artificial membrane models, whereas conflicting results were obtained with mitochondrial preparations where poor or even no sensitivity to PNs has been observed for the FFA-induced proton leak (413, 422–424). Moreover, the question arises as to how UCPs could ever effectively conduct proton in vivo, given the apparent affinity of UCPs for PNs and the high concentrations of nucleotides in vivo (in the millimolar range in cells) (425, 426).

A link between CoQ and UCP was first proposed more than two decades ago. Echtay and colleagues showed that UCPs (UCP1, 2, and 3) expressed in *E. coli* can be incorporated into liposomes to yield H^+^ transport activity. But no H^+^ influx into the vesicles was observed in the presence of an FFA (e.g., lauric acid) unless oxidized CoQ (CoQ_6_ or CoQ_10_) was provided, and the activated H^+^ uptake was sensitive to nucleotides (ADP or ATP) (427, 428). The findings were interpreted to indicate an obligatory role of CoQ in FFA-induced H^+^ transport by UCPs. This, however, was refuted in a subsequent study that used the same model system but with optimized conditions and showed that CoQ had no effect at all on lauric acid-dependent H^+^ transport by UCPs (429). Moreover, a yeast study reported that the activity of mouse UCP1 is similar whether expressed in CoQ-replete or CoQ-deficient mitochondria (714). Thus, it remains unclear whether there is any effect of CoQ on UCPs. Interestingly, a different mechanism by which CoQ could be involved in the regulation of UCP activity has been proposed. In experiments with isolated mitochondria, it was observed that the CoQ redox state modulates the sensitivity of the FFA-induced UCP activity to PNs (413, 423). A pioneering study with rat muscle mitochondria showed that GTP (a PN) was not able to inhibit lauric acid-induced UCP2/3-mediated uncoupling under respiratory state 3 (phosphorylating). However, an inhibitory effect was observed after the addition of the CII inhibitor malonate but not after the addition of the CIII Q_i_ site inhibitor antimycin A. The different sensitivities to PN were interpreted in the context of the differences in the mitochondrial CoQH_2_-to-CoQ ratios brought about by the addition of malonate and antimycin A. That is, CII inhibition decreases the level of reduced CoQ, whereas antimycin A prevents oxidation of CoQH_2_, even though it also slows respiration (423). Subsequently, it was further reported that malonate can prevent the suppression of UCP inhibition by PN when antimycin A is present, consistent with its effect on decreasing the ratio of reduced to oxidized CoQ (413).

**FIGURE 14.**
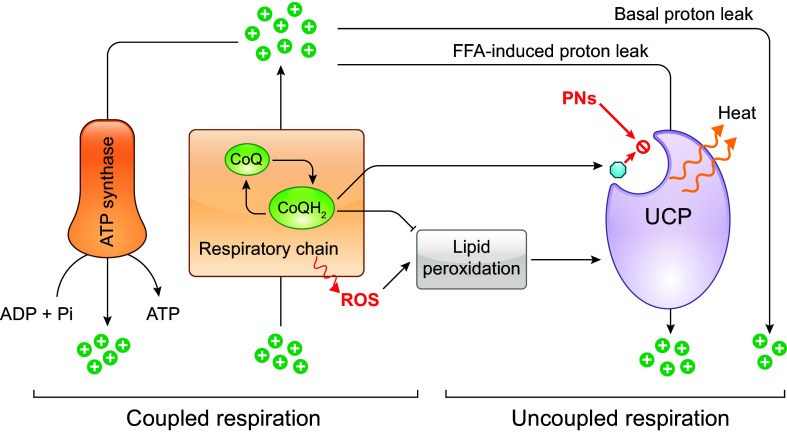
A tentative model of involvement of CoQ in UCP regulation. UCP-mediated uncoupling can be activated by free fatty acids (FFA), an effect that is sensitive to purine nucleotides (PNs). The CoQ redox state has no effect on basal and FFA-induced UCP-catalyzed H^+^ conductance in the absence of PNs, but it modulates the sensitivity of UCP to inhibition by PNs. At a given fatty acid concentration, increased CoQ reduction by the respiratory chain decreases the binding affinity of PN to UCP, possibly by directly interfering with PN binding to UCPs due to structural similarities, thereby promoting UCP activation. Conversely, at lower CoQH_2_ levels, no negative regulation occurs and UCP activity is inhibited by PNs. Additionally, it has been proposed that altered levels of CoQ or its redox state potentially could affect ROS-mediated UCP activation through its antioxidant activity against lipid peroxidation. See glossary for other abbreviations.

Similar results were obtained in later studies with BAT UCP1, various mitochondria under phosphorylating or nonphosphorylating conditions (from microorganisms, plants, or mammals), and different PNs (422, 430–432). The level of CoQ reduction under which the inhibition by PN becomes effective varies depending on conditions. For example, under phosphorylating, that is, coupled, conditions, relief of GTP inhibition was observed with a CoQ level of reduction above 85–88% in rat BAT mitochondria and above 57–64% in rat skeletal muscle mitochondria (423, 430). Of note, the endogenous CoQ redox state was shown to have no effect on the FFA-induced UCP activity in the absence of PNs (430–432). Considering these and taking into account the apparent affinity of UCPs for PNs and PN concentrations in vivo (in the millimolar range) (425, 426), it has been suggested that CoQ is involved in regulating the activity of UCP by modulating UCP sensitivity to PNs. The idea is that the high availability of CoQH_2_ in mitochondrial membranes promotes UCP activation by relieving the inhibition from PNs. Yet, under conditions leading to low CoQH_2_, such as low availability of respiratory substrates, PN sensitivity is turned on, leading to inactive UCP and more efficient ATP production (413, 421). This further implicates that different levels of mitochondrial CoQ reduction in different tissues or organisms could be a determinant of intrinsic UCP activity and metabolic efficiency. The exact mechanism for how CoQH_2_ prevents PN to inhibit FFA-activated UCPs remains to be elucidated. One postulate is that CoQH_2_ interferes directly with PN binding to UCPs (FIGURE 14) (421).

ROS-mediated UCP activation implicates a different pathway by which CoQ could play a role in the regulation of UCP activation. Incubating rat skeletal muscle and BAT mitochondria with xanthine plus xanthine oxidase to generate O_2_^•−^ was shown to increase mitochondrial proton conductance through the effects on UCPs (433). Later it was shown that the effect of O_2_^•−^ on UCP-mediated proton conductance is mediated through lipid peroxidation products, such as 4-hydroxynonenal (4-HNE) (434). CoQ could be involved as a site of ROS production during electron transport or CoQH_2_ could help to prevent lipid peroxidation (see sects. 3.1 and 3.2). However, as far as we know, altered levels of CoQ or its redox state have not been documented to affect UCP activation by O_2_^•−^. It also should be noted that activation of UCPs by O_2_^•−^ or HNE is still in dispute, as conflicting results have been reported (435, 436). The observed effects of O_2_^•−^ on stimulating uncoupling appear to be tissue specific by mechanisms unknown (437). Finally, and noteworthily, a more recent study reported that in UCP1-positive murine brown and beige adipocytes and in mouse brown adipose tissue, a 30–75% reduction of CoQ levels triggered a robust suppression of UCP1 expression that can be rescued by exogenous CoQ supplementation (119).

We have seen how CoQ has been implicated in modulating the activity of mPTP and UCPs, both of which are important players in the maintenance of the membrane potential (ΔΨm) across the IMM. It might be worth noting that there are several reports that measure ΔΨm in CoQ-deficient cells by using the fluorescence dye tetramethylrhodamine ethyl ester (TMRE), whose accumulation in mitochondria is strongly potential dependent. In human *COQ9* mutant cells with ≈20% residual CoQ_10_, a decrease of TMRE fluorescence was reported but no increase of mitochondrial ROS was found (as assessed by MitoSox staining intensity) (113). In contrast, *COQ2* mutant cells, which have ≈36% residual CoQ_10_, display an increase in MitoSox fluorescence but no change in TMRE fluorescence (113). Inhibition of COQ2 by 4-NB in wild-type or *ADCK3* mutant cells that lowers CoQ_10_ to below 50% increases the MitoSOX signal but does not always affect the TMRE signal in the mitochondria (336). With a similar approach, a ≈50% reduction in CoQ_10_ level in the T67 human glioma cells was found to be associated with a slight elevation of ΔΨm (338). Together these findings indicate a complex and bidirectional relationship between CoQ deficiency and the effect on ΔΨm.

### 4.4. Involvement of Plasma Membrane CoQ in the Regulation of the Cytosolic Redox State

As mentioned in a previous section, CoQ is a vital part of the constitutive PMRS (FIGURE 8). In addition to its antioxidant role in maintaining antioxidant levels in and around membranes, PMRS plays a role in the modulation of cytosolic NAD(P)^+^/NAD(P)H levels and the generation of NAD^+^ that is required to support glycolytic ATP production. This role is shown to be particularly essential for cell survival under conditions of mitochondrial energetic failure (438–440). Cells with damaged mitochondria, such as rho zero (ρ^0^) cells (devoid of mitochondrial DNA), upregulate PMRS activity, by inducing the expression of PMRS enzymes (438, 439, 441). The resulting elevated electron transfer rate across the plasma membrane, in addition to potentially strengthening antioxidant regeneration (CoQ, vitamin E, and vitamin C), generates more NAD^+^ from NADH (439, 441). Thus, by means of the PMRS, CoQ is involved in balancing the cytosolic NAD(P)^+^-to-NAD(P)H ratio, which is a key component of the redox metabolic state. But whether or when the availability of CoQ becomes limiting for the overall rate of electron flux across the plasma membrane is unknown. Interestingly, by inhibiting CoQ_10_ synthesis with 4-NB in T67 human glioma cells to ≈50% of normal levels, an increase in the overall activity of plasma membrane NADH oxidoreductase (measured as diphenyleneiodonium-sensitive oxygen consumption) was observed. This was likely mostly caused by the upregulation of the dehydrogenases of PMRS as a response to mitochondrial impairment (120). CoQ_10_ supplementation appeared to reverse the effect on NADH oxidoreductase but further increased the overall activity of plasma membrane NADH oxidoreductase, indicating that a ≈50% level of CoQ_10_ is not sufficient to support maximum stimulation of the PMRS (120).

### 4.5. Localization and Effects of CoQ in Lipid Membranes

Biological membranes are composed primarily of two layers of phospholipids, which are amphipathic molecules with a hydrophilic head group and a hydrophobic tail. Various biophysical methods, such as NMR spectroscopy, fluorescence, linear dichroism, differential scanning calorimetry, and neutron diffraction have been used to determine CoQ localization within lipid bilayers. However, no unequivocal conclusion about this can yet be drawn. Typically, two possible orientations are considered: one where CoQ molecules with tails of at least three isoprenoid units are fully embedded in the center of the bilayers lying flat parallel to the mid-membrane plane and one where the quinone ring resides near the region of the phospholipid head groups, with the isoprenoid chain encapsulated inside the bilayer toward the center (14–17, 20, 442–449). Other proposals include the formation of head-to-head aggregates, which are hypothesized to facilitate the location of the polar quinone ring in the hydrophobic middle of the bilayer (15, 17, 450). Empirical evidence is presented for both main models, leaving the question unresolved. Finally, an interesting variation is the suggestion that the CoQ redox state might affect where exactly the molecule is located, with one study suggesting that CoQ_10_H_2_ appears to be closer to the phospholipid head groups and the polar surface of the membrane than the oxidized form (17).

Does CoQ localization in lipid membranes affect membrane properties? A study with CoQ-deficient *E. coli* reported that *ΔubiG* mutants are more sensitive to high-salt stress and that supplementation with CoQ_10_ restores the mutants’ tolerance (19). More surprisingly, in response to hyperosmotic salt stress, the study reported to observe ≈100-fold increase in endogenous CoQ_8_ levels. Furthermore, in vitro liposome experiments showed greater hyperosmotic stress after the addition of CoQ_10_. However, a more recent study seems incompatible with the idea of a membrane-stabilizing and osmoprotective role of CoQ. Indeed, the function of the membrane protein ProP, a known protonmotive force-dependent osmosensory and osmoregulatory transporter, was found to be impaired in *ΔubiG* mutants (23). This suggests that the effect of CoQ_8_ deficiency on ProP function was the result of impaired respiration, not of altered physical properties of the membrane. Furthermore, this study showed that in wild-type *E. coli* respiration was significantly inhibited after osmotic upshift but was restored after prolonged culture in a high-osmotic medium. Taken together, the latter study proposed a key role of impaired respiration in the osmosensitivity induced by CoQ deficiency (23).

Nevertheless, multiple studies conducted in artificial liposomes reported other specific effects of CoQ_10_ on bilayer properties. In liposomes made of the phospholipid palmitoyl-2-oleoyl-sn-glycero-phosphocholine (POPC), the inclusion of CoQ_10_ leads to a membrane condensing effect, increased resistance toward rupture, and decreased membrane permeability (451). Also, in several model membranes, the addition of CoQ_10_ was shown to increase lipid packing, which could affect membrane rigidity and other physical properties such as mechanical stability and permeability (21–23). It might also be worth mentioning that solanesol, a nonquinone isoprenoid that has 9 isoprene subunits like CoQ_9_ but lacks the quinone head group, was used to determine whether the quinone ring is essential for the effects induced by CoQ_10_. Contradictory findings about this were reported. Solanesol was shown to be as effective as CoQ_10_ in protecting against collapse due to high-salt stress in liposomes prepared from a lipid mixture but had no, or very little, effect on lipid packing, membrane density, or permeability in POPC liposomes and in an IMM membrane model (19, 21, 22, 451).

Although the liposomes used in these investigations were built from biologically relevant lipids (such as POPC), the lipid composition did not exactly reflect that of native membranes. Between different species and tissues but also among different subcellular compartments, lipid content and compositions vary considerably, and thus so might the interactions with CoQ. For example, it has been suggested, but remains to be investigated, that CoQ in the IMM, where the content of the well-known membrane stabilizer cholesterol is low and protein load is high, might create a mechanical barrier for transmembrane proton leaks (16).

## 5. CoQ BIOSYNTHESIS

Endogenous synthesis is the main source of cellular CoQ. The biosynthesis of CoQ is highly conserved, especially among eukaryotes, and has been investigated in different model organisms. To date, it has been best characterized in *E. coli* and the budding yeast *S. cerevisiae*, but the knowledge obtained from the model systems has been shown to apply closely to higher organisms including humans. The process of CoQ biosynthesis begins with the formation of the lipophilic isoprenoid side chain and the synthesis of 4-hydroxybenzoic acid (4-HB), which is the universal precursor of the aromatic head group of CoQ. 4-HB is derived from different precursors depending on the species. The isoprenoid side chain is made in similar ways in all eukaryotes but by a different pathway in *E. coli* (452, 453). Briefly, after the attachment of the isoprenoid tail to the quinone ring, the ring group undergoes a series of modifications to yield the final product. These steps are catalyzed by several proteins in sequential steps. It has now been widely accepted that most of the pathway components need to assemble into a multisubunit protein complex for the biosynthetic reactions to occur efficiently and produce CoQ (25–28, 454). However, many molecular, structural, and mechanistic details remain to be elucidated. A related open question is how the whole process and thus the final yield of CoQ is regulated. Below we summarize the main understanding of CoQ biosynthesis in budding yeast *S. cerevisiae* and *Mus musculus* (mouse), which are the most detailed and most relevant models for humans, respectively. For additional information, as well as for CoQ biosynthesis in *E. coli* and other organisms, the reader can refer to many comprehensive review articles (7, 10, 11, 25–27, 37, 109, 455–465).

### 5.1. CoQ Biosynthetic Pathways in Eukaryotes

CoQ synthesis in eukaryotes takes place in the mitochondria, more specifically on the matrix side of the IMM. Most eukaryotic genes and proteins required for CoQ biosynthesis are given COQ names plus a number based on the order in which they were identified. They are nucleus encoded in all well-studied species and contain mitochondrial targeting sequences. The main precursor of the aromatic quinone ring used by both yeast and mammals is 4-HB, which can be derived from tyrosine, but some steps of its synthesis pathway remain unidentified (464, 466). Human cells can also use phenylalanine as a precursor for 4-HB because phenylalanine hydroxylase is present in human cells. However, this enzyme is absent in yeast (456). Furthermore, mammalian cells may also be able to use polyphenols in CoQ biosynthesis, such as kaempferol (467). The pathways by which polyphenols enter into the biosynthesis of CoQ remain to be fully delineated. See Ref. 458 for more details.

In eukaryotes, isoprenoid side chains are synthesized via the mevalonate pathway, which begins with acetyl-CoA and produces isopentenyl pyrophosphate (IPP). Isomerization of IPP generates dimethylallyl pyrophosphate (DMAPP or DPP). Catalyzed by farnesyl diphosphate synthase (FDPS), these two metabolites generate farnesyl pyrophosphate (FPP), which is predominantly localized to peroxisomes and is the precursor of several biomolecules with distinct functions including CoQ, dolichols, prenylated proteins, and sterols (455). In the mitochondria, condensation of FPP with several IPP molecules catalyzed by a trans-prenyl-transferase ultimately delivers an isoprenoid side chain of a specific length for CoQ biosynthesis. To date, it is not fully understood how the building block of the isoprenoid tail and the ring precursors are transported into mitochondria for CoQ biosynthesis. A recent study identified Hem25p, a mitochondrial glycine transporter, required for mitochondrial IPP transport for CoQ biosynthesis in the yeast *S. cerevisiae*. However, its mammalian homolog SLC25A38 does not function as an IPP transporter (468, 469).

How CoQ moves out of the mitochondria to other various membranous organelles is not yet understood. However, several possible routes have been considered to have a role in the intracellular distribution of CoQ. One or a combination of them could be responsible for CoQ transport in different eukaryotic cells. Here are a few proposals. An in vitro study that followed the appearance of newly synthesized, labeled CoQ_10_ in human leukemia cells (HL60) suggested that the endomembrane system might be involved (353). STARD7, a lipid-binding protein that is known to be required for phosphatidylcholine delivery to the mitochondria, has been shown to play a role in CoQ transport to the plasma membrane and protection of ferroptosis (400, 469). Saposin B, a small protein with lipid binding properties involved in glycosphingolipid metabolism, binds to CoQ, and its precursor prosaposin level was shown to affect the levels of CoQ in human HepG2 and CaCo-2 cells (470–473). Yeast studies showed that the bridgelike structure that forms at the membrane contact site between the endoplasmic reticulum (ER) and the outer mitochondrial membrane, named ERMES, is necessary for CoQ export from the mitochondria (474). Finally, it is worth noting that, in a mouse model, deficiency of COQ7 was shown to affect CoQ distribution within mitochondrial membranes, more specifically between the IMM and OMM (8). Readers interested in this topic can refer to Ref. 360 and the references therein for further information.

#### 5.1.1. The budding yeast *S. cerevisiae.*

As mentioned, in *S. cerevisiae* the benzoquinone ring precursor for CoQ 4-HB can be derived from the metabolism of tyrosine. Five aminotransferases (Aro8, Aro9, Bat2, Bna3, and Aat2) have recently been shown to each be able to catalyze the deamination of tyrosine to 4-hydroxyphenylpyruvate (4-HPP), the first step of 4-HB production from tyrosine (456, 475). The last step is the oxidation of 4-hydroxbenzaldehyde (4-HBz) to 4-HB, which is catalyzed by the aldehyde dehydrogenase Hfd1, mainly located in the OMM (456, 476, 477). Other details of the tyrosine-to-4-HB pathway are not yet clear. Of note, expression of ALDH3A1, a human homolog of yeast Hfd1, restored CoQ_6_ biosynthesis in the *hfd1* yeast mutant (456). However, it remains to be determined whether CoQ biosynthesis in mammals requires ALDH3A1 (478). In addition to tyrosine, 4-HPP can also be derived from the shikimate pathway, also known as the chorismate biosynthesis pathway, which is not present in animals (479, 480). Chorismate is utilized as a substrate in a number of biosynthetic pathways of aromatic compounds. One branch generates *para*-aminobenzoic acid (pABA) via a two-step reaction catalyzed by Abz1 and Abz2, respectively (481, 482). pABA, a well-known precursor of folic acid (an essential B vitamin), can serve as an alternative ring precursor of CoQ biosynthesis in yeast but not in *E. coli* or mammalian cells (482, 483). The CoQ intermediates derived from pABA contain an amino group (–NH_2_) at the C4 position instead of a hydroxyl group (–OH), which needs to be replaced to yield CoQ. The conversion of the NH_2_ into an OH is proposed to occur on the early intermediate 3-hexaprenyl-4-amino-5-hydroxybenzoic acid (HHAB) and relies on the Coq6 enzyme (484–486). Finally, like *E. coli* and mammals, *S. cerevisiae* is able to use *para*-coumarate and resveratrol as CoQ ring precursors (487). The discovery of the ability of yeast to use different sources as the ring precursor of CoQ biosynthesis raises the question of how the cells interpret particular media conditions and adapt to different nutrient mixtures. Competition experiments using ^13^C-aromatic ring precursors suggest that pABA and 4-HB provided exogenously are equally efficient at generating CoQ_6_ (482). However, their relative contribution to CoQ_6_ production under physiological conditions is not known.

**FIGURE 15.**
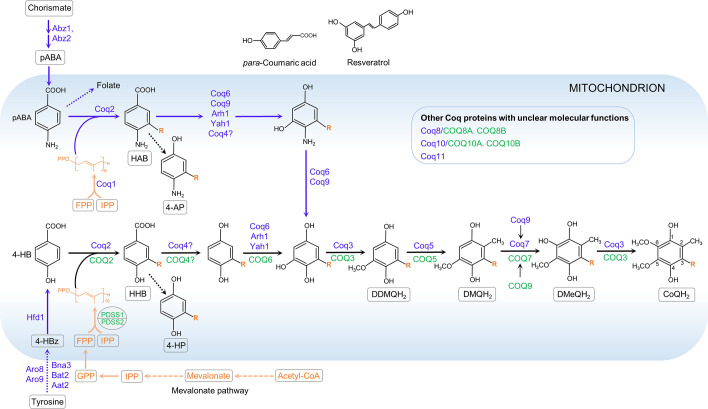
The CoQ biosynthesis pathway in the yeast *S. cerevisiae* and humans. The proteins are in blue (*S. cerevisiae*) or green (humans). Dotted arrows designate multiple-step reactions, and the steps that are specific to yeast are shown in blue. R indicates the poly-isoprenoid tail. The synthesis of the isoprenoid depends on the mevalonate pathway, which produces the biosynthetic precursors of isoprenoids. Coq1 in yeast and a heterotetrameric protein formed by PDSS1 and PDSS2 in humans determine the number of isoprene units in the polyisoprene tail. The main ring precursor used by both yeast and humans is 4-hydroxybenzoic acid (4-HB), synthesized from tyrosine in the cytosol. The first and last steps of this pathway have been defined in the yeast, where 5 aminotransferases, Aro8, Aro9, Bat2, Bna3, and Aat2, redundantly convert tyrosine to 4-hydroxyphenylpyruvate (4-HPP) and the last step is catalyzed by Hfd1. After its transport into mitochondria, Coq2/COQ2 attaches the isoprenoid tail to 4-HB. Subsequent to this step, the CoQ ring undergoes several sequential modifications before yielding CoQ. The intermediates detected in yeast are shown. A recent study using a human embryonic kidney cell line (HEK293) showed that the decarboxylation and hydroxylation of position C1 occur in a single oxidative decarboxylation step and it is catalyzed by COQ4. The reaction catalyzed by COQ4 preferentially occurs before the C5 hydroxylation by COQ6; however, the alternative sequence of reactions, that is COQ6 and COQ3 may act before COQ4, is also possible (488). Yeast can also utilize *para*-aminobenzoic acid (pABA) for CoQ synthesis, which is made from chorismate in 2 steps catalyzed by Abz1 and Abz2. The nitrogen-containing intermediates generated from its utilization are also depicted. Coq6 and Coq9 are able to deaminate the ring C4 position on the intermediate derived from pABA. Whether and where Coq4 is involved before the deamination step remains to be demonstrated. pABA is also an intermediate in the synthesis of folate in the yeast. Two additional compounds that can also serve as ring precursors both in *S. cerevisiae* and mammals are shown at *top*: *para*-coumaric acid and resveratrol. The absence of Coq6 activity leads to the accumulation of 3-hexaprenyl-4-aminophenol (4-AP) and 3-hexaprenyl-4-hydroxyphenol (4-HP) when yeast cells are grown in pABA and 4-HB, respectively. The Coq/COQ proteins with unclear molecular functions are listed in a box. CoQ and intermediates are shown in their reduced forms. DDMQH_2_, 3-hexaprenyl-5-methoxy-1,4-benzenediol; DMeQH_2_, 2-methyl-3-hexaprenyl-5-methoxy-1,4,6-benzenetriol; DMQH_2_, 2-methyl-3-hexaprenyl-5-methoxy-1,4-benzenediol; FPP, farnesyl diphosphate; GPP, geranyl pyrophosphate; HAB, 3-hexaprenyl-4-aminobenzoic acid; 4-HBz, 4-hydroxbenzaldehyde; HHAB, 4-amino-3-hexaprenyl-5-hydroxybenzoic acid; HHB, 3-hexaprenyl-4-HB; IPP, isopentenyl pyrophosphate. See glossary for other abbreviations.

At least 12 gene products, namely Coq1–Coq9, Coq11, Arh1, and Yah1, have been identified in *S. cerevisiae* to be necessary for making CoQ_6_ from the ring and side chain precursors (FIGURE 15) (25–27, 483, 489–495). Coq2 is an integral membrane protein, whereas other Coq polypeptides (Coq1, Coq3–Coq11) are localized to the matrix side of the IMM. Coq1 (trans-prenyl-transferase) synthesizes the hexaprenyl diphosphate tail. Coq2 (polyprenyl diphosphate transferase) then attaches the tail to aromatic ring precursors such as 4-HB. At this time, four Coq proteins have been identified to participate in the subsequent modifications of the CoQ benzoquinone ring. They include Coq3 (O-methyltransferase), Coq5 (C-methyltransferase), and Coq6 and Coq7 (hydroxylases). The function of Coq6 requires ferredoxin (Yah1) and ferredoxin reductase (Arh1), both of which are mitochondrial iron-sulfur [2Fe-2S] redox proteins with a known role in heme A biosynthesis and in Fe-S cluster assembly (483, 489). Their function in CoQ_6_ biosynthesis is predicted to be as electron donors for Coq6’s activity. *coq1*–*coq9* deletion mutants lack CoQ_6_ and are therefore respiration incompetent, as they are unable to grow on nonfermentable carbon sources such as glycerol or ethanol (25, 496, 497).

It has been well established that a subset of CoQ_6_ pathway components in *S. cerevisiae* form a supramolecular protein complex that carries out all the reactions subsequent to the Coq2-catalyzed prenylation. This large multi-Coq protein complex in the mitochondria containing Coq3–9 and Coq11 has been termed the CoQ synthome or complex Q. To avoid confusion, we spell it out as “the biosynthetic CoQ complex” for the rest of this review. The complex is necessary for the stability and function of its individual constituents, which is supported by the findings that *1*) decreased levels of other Coq polypeptides are generally found in strains harboring *coq* gene deletions but no or a smaller effect was observed on the expression of other Coq proteins in mutants harboring point mutations instead of deletions and *2*) only the early intermediates of CoQ_6_ biosynthesis, 3-hexaprenyl-4-hydroxybenzoic acid (HHB) and 3-hexaprenyl-4-aminobenzoic acid (HAB), produced by the prenylation of 4-HB and pABA, respectively, are readily detected in each of the *coq3*–*coq9* null yeast mutants. The intermediates that should accumulate because they are the substrates of the missing enzymatic reactions are not accumulating in detectable amounts (TABLE 3) (25, 465, 485, 492, 498–506). A high-molecular weight protein complex comprising Coq proteins was detected by size exclusion chromatography, and several Coq polypeptides were shown to comigrate in blue-native polyacrylamide gel electrophoresis (BN-PAGE) as a high-molecular weight band (498, 499, 503, 506). Nevertheless, further characterization of the CoQ biosynthetic complex remains necessary to determine its exact composition, subunit stoichiometry, and three-dimensional (3-D) structure and the likely dynamic nature of its assembly.

**Table 3.** Yeast S. cerevisiae CoQ biosynthetic mutants

Gene	CoQ_6_ Synthesis	Respiratory Growth	CoQ Intermediates	
			−	+ Coq8 OE
*coq1*	Absent	Absent	Not found	Not found
*coq2*	Absent	Absent	Not found	Not found
*coq3*	Absent	Absent	HHB↑, HAB↑*	HHB↑, HAB↑*
*coq4*	Absent	Absent	HHB↑, HAB↑*	HHB↑, HHAB↑*
*coq5*	Absent	Absent	HHB↑, HAB↑*	DDMQ_6_↑, DDMQ_6_↑*
*coq6*	Absent	Absent	HHB↑, HAB↑*	4-HP↑, 4-AP↑*
*coq7*	Absent	Absent	HHB↑, HAB↑*	DMQ_6_↑, DMQ_6_↑*
*coq8*	Absent	Absent	HHB↑, HAB↑*	CoQ_6_
*coq9*	Absent	Absent	HHB↑, HAB↑*	DMQ_6_↑, 4-HP↑, 4-AP↑*
*coq10*	Impaired	Impaired	HHB↑, DMQ_6_↓ HAB↑ *	HHB↑, HAB↑*
*coq11*	Impaired	Not affected	Not found	Not found

*In the presence of pABA as the labeled precursor instead of labeled 4-HB. For references, see sect. 5.1.1. 4-AP, 4-aminophenol; 4-HP, 3-hexaprenyl-4-hydroxyphenol; DDMQ_6_, demethyldemethoxy-CoQ_6_; DMQ_6_, demethoxy-CoQ_6_; OE, overexpression. See glossary for other abbreviations.

Among the Coq proteins whose functions are not fully understood, namely Coq4, Coq8, Coq9, Coq10, and Coq11, Coq4 and Coq8 are proposed to be involved in the organization or maintenance of the biosynthetic CoQ complex, though this has not yet been directly demonstrated (484, 498, 499, 501, 506). A recent study, however, shows that, in addition to the proposed structural role in the organization of the complex, human COQ4 acts as an oxidative decarboxylase, substituting in a single step the carboxylic acid group with a hydroxyl group on carbon C1 of CoQ precursors (488). For Coq8, its overexpression (OE) in *coq3Δ*, *coq5Δ*, *coq6Δ, coq7Δ*, or *coq9Δ* cells has been shown to elevate the steady-state levels of other Coq proteins and to stabilize the biosynthetic CoQ complex and, as a result, to allow the accumulation of the corresponding CoQ biosynthetic intermediates (484, 501). Furthermore, Coq8 might also enable CoQ intermediate extraction from the IMM to facilitate their availability to CoQ aromatic ring-modifying enzymes (507, 508). Finally, it has been proposed that Coq8 and its ATPase activity may have a role in the organization of the biosynthetic CoQ complex into discrete domains within mitochondria, observed as puncta located near endoplasmic reticulum (ER)-mitochondria contact sites (502). Such localization might be required for orchestrating CoQ synthesis in the IMM and its export from mitochondria (474, 502).

Coq9 is a member of the biosynthetic CoQ complex and is required for optimal function of Coq6 and Coq7 (484, 485, 493, 498). In fact, a physical association between human COQ9 and COQ7 was demonstrated in vitro in a cell-free protein expression system, and conserved residues around the lipid-binding site of COQ9 are shown to be important for maintaining the interaction with COQ7 (509). Of all Coq proteins, Coq11 is the most different and, so far, the least understood. Unlike the other *coq* mutants lacking one of the components of the biosynthetic CoQ complex, *coq11Δ* cells have no decrease but even a slight elevation in the levels of other Coq proteins and also lack evidence for destabilization of the biosynthetic CoQ complex (502, 510). Moreover, *coq11Δ* cells retain the ability to make CoQ_6_, though their de novo CoQ_6_ biosynthesis is significantly impaired. These findings, taken together, have prompted speculation that the absence of Coq11 does not impair but enhances the CoQ biosynthetic machinery and that the net loss of CoQ biosynthesis in *coq11Δ* mutants suggests a function for Coq11 that is related to other aspects of CoQ metabolism rather than to biosynthesis per se. On this note, Coq11 was identified as part of the mitochondrial organization of gene expression (MIOREX) complexes, which are large assemblies of ribosomes comprising factors that are involved in mitochondrial gene expression (511). No functional homolog of Coq11 has so far been found in animal genomes. In silico analysis indicates that it belongs to the atypical short-chain dehydrogenase/reductase (SDR) superfamily and the closest higher eukaryotic Coq11-like protein is NDUFA9, a subunit of CI (492).

Along with Coq1 and Coq2, Coq10 appears to not be part of the biosynthetic CoQ complex (498). Despite reduced de novo CoQ_6_ synthesis efficiency, nearly normal steady-state levels of CoQ_6_ were detected in *coq10Δ* cells. Nevertheless, the defect in respiratory function as well as a higher sensitivity to PUFA indicate compromised CoQ function (331, 512). Structural determination of a Coq10 homolog, CC1736 from the bacterium *Caulobacter crescentus*, identified a lipophilic START (steroidogenic acute regulatory-related lipid transfer) domain, which is known to bind lipid in a hydrophobic binding pocket and to be involved in the nonvesicular intracellular transport of lipids and sterols (512). Thus, it has been hypothesized that Coq10 functions as a chaperone to facilitate CoQ delivery to its sites of action, especially in the IMM (331, 512, 513). How loss of Coq10 affects de novo synthesis of CoQ_6_ is not understood, though destabilization of the biosynthetic CoQ complex in the absence of Coq10 was suggested by some observations (331, 498, 501, 502, 510, 512). Finally, it is worth noting that a more recent study reported a functional interaction between Coq10 and Coq11. Specifically, deletion of *COQ11* alleviates the CoQ deficiency phenotype of *coq10Δ* mutants and this was associated with a significant elevation of mitochondrial CoQ_6_ (510). Furthermore, the presence of Coq11 appears to be needed for Coq8 overexpression to be able to confer a beneficial effect in the *coq10Δ* mutant (510).

#### 5.1.2. Mouse CoQ biosynthesis mutants.

Except for *coq11*, homologs of the yeast *coq* genes are all present in mammals (408, 512, 514–520). In rodents and humans, the enzymes responsible for the first committed step of CoQ, the assembly and elongation of the isoprenoid side chain, are heterotetramers of two protein subunits rather than monomeric enzymes as in budding yeast (Coq1) and *E. coli* (IspB) (521). The genes encoding the two subunits are designated *Pdss1* and *Pdss2* in mice and *PDSS1* and *PDSS2* in humans. In mice, complete elimination of CoQ biosynthesis causes embryonic lethality, as has already been demonstrated with mutations in four *Coq* genes: *Pdss2*, *Coq3*, *Coq7,* and *Coq8b* (8, 29–31, 522). This is not surprising since a lethal phenotype is typical for mice with mutations in genes encoding essential ETC components, e.g., the “Rieske” iron-sulfur protein (*Risp*) of CIII, *cyt c*, and CIV assembly factor *Cox10* (523–525). TABLE 4 lists all the CoQ deficiency mouse models reported in the literature so far.

**Table 4.** Mouse CoQ biosynthesis mutants

Mouse Mutant	CoQ and CoQ Intermediates	Gross Phenotype
*Spontaneous mutation*		
*Pdss2^kd/kd^*	Widespread moderate to severe CoQ deficiency	Nephrotic syndrome, kidney failure, impaired motor behavior, plasma lipid abnormalities, death by 9.5 mo of age
*Germline mutation*		
*Coq3^+/−^*	Normal CoQ_9_ levels	Wild-type appearance, normal life span
*Coq7^+/−^*	Normal level of total CoQ_9_, mild CoQ_9_ loss in the IMM	Wild-type appearance, 30% longer life span
*Coq8a/ADCK3^−/−^*	Moderate CoQ_9_ loss in skeletal muscles, kidney, and liver, not in cerebellum and serum	Progressive cerebellar ataxia and mild exercise intolerance, normal life span
*Coq9^R239X^*	Global and severe CoQ_9_ loss	Poor overall growth, impaired motor function, progressive paralysis, death between 3 and 6 mo of age
*Coq9^Q95X^*	Moderate CoQ_9_ loss in the kidney and cerebrum, severe CoQ_9_ loss in skeletal muscle	Adult-onset mild myopathy
*Conditional knockout*		
*Pdss2* podocyte KO *Nphs1-cre;Pdss2^loxP/loxP^*	ND	Nephrotic syndrome
*Pdss2* renal tubular epithelium KO *PEPCK-cre;Pdss2^loxP/loxP^*	ND	Absence of overt phenotype
*Pdss2* Purkinje cell KO *Pcp2-cre;Pdss2^loxP/−^*	ND	Ataxia-like symptoms at 9.5 mo
*Pdss2* Purkinje cell KO *Pax2-cre;Pdss2 ^loxP/−^*	ND	Death within the first 36 h of life
*Pdss2* liver KO *Alb-cre;Pdss2 ^loxP/loxP^*	Severe CoQ_9_ loss in the liver	Absence of overt phenotype, altered amino acid and DNA metabolism
*Pdss2* dopaminergic neuron KO *DAT-cre;Pdss2^loxP/loxP^*	ND	Loss of TH-positive neurons, motor deficit
*Coq6* podocyte *KO Nphs2-cre;Coq6^loxP/loxP^*		Renal dysfunction, death at ≈10 mo
*Coq7* liver KO *Alb-cre;Coq7 ^loxP/loxP^*	Severe CoQ_9_ loss and DMQ_9_ accumulation in the liver	Absence of overt phenotype, normal life span
*Coq7* whole body inducible KO *CAG-creER^T2^;Coq7 ^loxP/loxP^*	Global and severe CoQ_9_ loss and DMQ_9_ accumulation	Weight loss, loss of coat hair, kidney dysfunction, elevated blood lactate, short life span
*Coq8a/ADCK3* Purkinje cell KO (*Pcp2-Cre; Coq8a^loxP/loxP^*)	ND	Ataxia
*Coq8b/ADCK4* podocyte KO (*Nphs2-Cre; Coq8b^loxP/loxP^*)	ND	Renal disease, death at ≈12 mo

IMM, inner mitochondrial membrane; KO, knockout; ND, not determined; TH, tyrosine hydroxylase. For references, see sect. 5.1.2.

##### 5.1.2.1. pdss2 mouse mutants.

The first reported mutation in *Pdss2* appeared spontaneously in an inbred strain of mice and was designated the “kidney disease” (*kd*) allele because the most prominent phenotype of homozygous mutant mice is kidney dysfunction leading to renal failure (344). The *kd* allele is a missense mutation resulting in the amino acid change V117M (30). *Pdss2^kd/kd^* mice develop nephrotic syndrome recognizable at ≈10 wk of age, with proteinuria, excessive drinking, and visceral epithelial abnormalities, accompanied by collapsing glomerulopathy. The mice die by 9.5 mo from renal failure (526, 527). Plasma lipid abnormalities such as high levels of serum triglycerides and cholesterol have also been previously observed to develop with age in *Pdss2^kd/kd^* mice (526). No overt manifestations were observed in all other tissues examined, including brain, retina, liver, and skeletal muscles (30, 87). Widespread CoQ deficiency was observed in *Pdss2^kd/kd^* mice: in the liver (≈60–70% of the mean of control mice), brain (20–28%), kidney (14–28%), and muscle (20–35%) (30, 124). The currently prevailing idea is that kidney disease in *Pdss2^kd/kd^* mice, and perhaps in some other CoQ deficiency conditions as well, results from podocyte dysfunction and is caused by oxidative stress mediated by impairment of the sulfides oxidation pathway (87, 90, 124).

Tissue-specific conditional *Pdss2* KO models targeting renal glomeruli or tubules demonstrated that renal glomerular podocytes indeed have a particularly high sensitivity to *Pdss2* deletion. Specifically, *Podocin-cre;Pdss2^loxP/loxP^* mice with podocyte-specific deletion of *Pdss2* were shown to exhibit a phenotype similar to *Pdss2^kd/kd^* mice, whereas no renal abnormality was observed in *PEPCK-cre;Pdss2^loxP/loxP^* mice that express Cre predominantly in the renal tubular epithelium (30). More recently, a single-nucleus RNA-Seq study on kidneys of *Pdss2^kd/kd^* mice revealed a podocyte-specific perturbation of the Braf/Mapk pathway, which is linked to altered PUFA metabolism and elevation of *Gpx4*, which, as mentioned in sect. 4.1, encodes a glutathione peroxidase playing a key role in protecting against lipid peroxidation and ferroptotic cell death. Moreover, it was shown that GDC-0879, a selective B-Raf inhibitor, can ameliorate kidney injury in *Pdss2^kd/kd^* mice, pointing to a new pathway that may contribute to the renal pathophysiology induced by CoQ deficiency (406). Models with *Pdss2* specifically ablated in the cerebellum, dopaminergic neurons, liver, or monocytes were also reported. *Pcp2-cre;Pdss2 ^loxP/−^* mice with postnatal *Pdss2* deletion in cerebellar Purkinje cells and retinal bipolar neurons exhibited a significant loss in Purkinje cells by 6 mo of age and developed ataxia-like symptoms at 9.5 mo (528). On the other hand, *Pax2-cre;Pdss2^loxP/−^* mice with a targeted deletion of *Pdss2* in the midbrain-hindbrain at embryonic day 9.5 died shortly after birth (528). Conditional *Pdss2* KO in dopaminergic neurons was shown to cause loss of tyrosine hydroxylase (TH)-positive neurons from the substantia nigra and consequently have decreased motor coordination and locomotive activities (529). Of note, although *Pdss2^kd/kd^* mice suffer mostly from kidney dysfunction, they were also reported to display motor phenotypes (529). On the other hand, mice with liver-specific KO of *Pdss2* (*Alb-Cre*;*Pdss2 ^loxP/loxP^)* showed no overt manifestations of liver dysfunction, despite very low CoQ levels (30). Significantly elevated plasma cholesterol was seen in both the podocyte- and liver-specific *Pdss2* KOs, indicating that *Pdss2* gene defects in hepatocyte and podocytes both contribute to the plasma lipid abnormalities observed in *Pdss2^kd/kd^* mice (30).

##### 5.1.2.2. coq6 mouse mutants.

A podocyte-specific *Coq6* KO mouse model (*Nphs2-cre;Coq6^loxP/loxP^*) was generated and described to recapitulate aspects of the pathology of focal segmental glomerulosclerosis (FSGS) observed in patients with *COQ6* mutations (530). *Nphs2-cre;Coq6^loxP/loxP^* mice showed an onset of proteinuria at 5 mo, and progressive FSGS was observed in 10-mo-old mice, which then became moribund. 2,4-DHB significantly inhibited disease progression and improved life expectancy. This is a surprising result. Indeed, 3,4-3,4-dihydroxybenzoate (3,4-DHB), but not 2,4-DHB, was shown to rescue yeast *COQ6* deletion mutants, which is also the logical outcome (see sect. 5.1.2.3 and sect. 5.4 for additional details about 2,4-DHB). No mechanism for the reported benefit of 2,4-DHB treatment in this conditional *Coq6* KO model has been proposed, and the effect of 2,4-DHB treatment on the tissue levels of CoQ was not determined in the study.

##### 5.1.2.3. coq7 mouse mutants.

Both heterozygous *Coq3^+/−^* and *Coq7^+/−^* mice look superficially wild type and show normal tissue levels of CoQ_9_. However, in contrast to *Coq3^+/−^* mice, a variety of phenotypes were found in *Coq7* hemizygotes (*Coq7^+/−^*), including respiratory chain deficiency, decreased ATP production, higher mitochondrial oxidative stress, increased expression of HIF-1α, and more, all of which were not observed with *Coq3^+/−^* mice (8, 122, 531). *Coq7^+/−^* mice also display an increased life span of up to 30% (532). The total amount of CoQ was the same in the mitochondria of *Coq7^+/−^* mice as in wild-type control mice, but CoQ quantification in mitochondrial membrane fractions revealed a decrease in the IMM with a concomitant elevation in the OMM. This local CoQ deficiency in the IMM is the most likely underlying cause of the phenotypic abnormalities seen in *Coq7^+/−^* mutant mice (8). These findings suggest that, at least under certain conditions, a mild reduction in CoQ level in the IMM is sufficient to induce mitochondrial dysfunction leading to phenotypic consequences.

A global conditional KO mouse model for *Coq7* was generated using a transgene expressing a tamoxifen-dependent CRE recombinase (*CreER^T2^*) (123). Induction of *Coq7* KO by tamoxifen injection at ≈2 mo of age led to a global, gradual loss of CoQ, impairment of mitochondrial function, gradual development of disease phenotypes, and shortened life span. The mice’s phenotypes, examined at 6 mo after KO induction, include severe growth retardation, loss of coat hair, kidney dysfunction, the elevation of blood lactate, and decreased levels of fasting blood glucose and nonfasting plasma triglycerides. Although the ultimate cause of death is unknown, it is unlikely that the mice died from heart failure, as at ≈8 mo after KO induction, shortly before the mice started to die, echocardiographic examination of the mutant and control mice found similar baseline parameters of systolic and diastolic function, despite the fact that there was hardly any CoQ left in the heart (123). At ≈2 mo after *Coq7* KO induction there was already an ≈80% reduction in CoQ_9_ levels in all the tissue examined, including the heart, kidney, skeletal muscles, and intestine. At 6 mo, the level of CoQ in the strongly affected tissues averaged only 10–15% of controls. However, the mutant mice survived for almost an additional 6 mo, with a median survival of ≈276 days.

2,4-DHB is a structural analog of 4-HB. It can be used as an alternative ring precursor of CoQ when provided and allows for the bypass of a COQ7 defect (see sect. 5.4 for additional details about 2,4-DHB). 2,4-DHB treatment was shown to lead to a dose-dependent phenotypic rescue of *Coq7* KO mice (115). More strikingly, at the dose of 1 g/kg body wt/day, provision of 2,4-DHB starting 2 wk after completion of tamoxifen injection led to only partial restoration of tissue CoQ levels but that nonetheless was sufficient for an almost complete rescue of the mutant phenotypes. In the heart, kidney, and skeletal muscles, the CoQ_9_ levels of treated KO mice were increased to ∼30–40% of the wild-type levels. However, except for their lower body weight, treated KO mutants were visually indistinguishable from the wild type and could live a full life span (123). These findings indicate that CoQ content in most tissues is probably maintained at largely excess levels for viability, at least under laboratory conditions. Furthermore, a late-onset 2,4-DHB treatment starting as late as 6 mo after KO induction, a time point when untreated KO mice already displayed severe, sublethal, phenotypes, was also able to successfully rescue the KO mice, demonstrating that most or all CoQ deficiency phenotypes are reversible (123).

##### 5.1.2.4. coq8 mouse mutants.

Two coorthologs of yeast *COQ8* have been identified in mice: *Coq8a* (also known as *Adck3*) and *Coq8b* (also known as *Adck4)* (507). A study reported that *Coq8a* KO (*Coq8a*^−/−^) mice are viable and show normal growth and life span. However, they nonetheless develop a slowly progressive cerebellar ataxia and mild exercise intolerance. A specific defect in the cerebellar Purkinje cell layer was found in the central nervous system of *Coq8a*^−/−^ mice. Histological analyses revealed abnormal mitochondrial morphology in the skeletal muscle, but mitochondrial respiration and metabolites of central carbon metabolism (e.g., lactate) were not altered. A variable reduction in CoQ levels was observed in the skeletal tissue, kidney, and liver of *Coq8a*^−/−^ mice, ranging from 20% to 50% decrease at 7 mo of age. However, cerebellar CoQ levels were normal, suggesting that CoQ loss is most likely restricted to Purkinje cells, which only make up a small fraction of the cerebellum (<0.1%) (507). The study also reported loss of the biosynthetic CoQ complex proteins (COQ3–COQ9), in several tissues, to varying degrees. The mild and tissue-specific phenotype of *Coq8a*^−/−^ mice is consistent with a regulatory function of COQ8 in CoQ biosynthesis, with its role in assisting in biosynthetic CoQ complex formation and stability, and with the existence of two close homologs (COQ8A and COQ8B), which might slightly differ in biochemical activity and in tissue-specific expression but nonetheless complement each other’s loss. It has been speculated that more distantly related COQ8A/B homologs (ADCK1, ADCK2, and ADCK5) could also contribute (507).

The high sensitivity of Purkinje neurons to *Coq8a* mutation is further corroborated by the finding that deletion of COQ8A in Purkinje cells was sufficient to cause ataxia in a conditional mouse model (533). Of particular importance is to note that cerebellar ataxia is a typical symptom of human patients harboring mutations in *COQ8A*, which is well recapitulated in these mouse models (507, 533). In contrast, whole body KO of *Coq8b* in mice is lethal. As *COQ8B* mutations have been implicated in steroid-resistant nephrotic syndrome (SRNS) in patients, a podocyte-specific *Coq8b* KO model (*Nphs2-Cre;Coq8b^loxP/loxP^*) was generated and the mice were shown to present with glomerulopathy and renal dysfunction that started at ∼4 mo (522, 534). The kidney function of the mutant mice continued to decline with time and progressed to renal failure and death by 12 mo (522). It was also observed that the protein levels of COQ3, COQ5, and COQ9 were decreased in *Coq8b* KO podocytes. Surprisingly, treatment with 2,4-DHB was shown to rescue the disease phenotypes and the shorter survival of *Nphs2-Cre;Coq8b^loxP/loxP^* mice (522). As mentioned above, 2,4-DHB is an alternative ring precursor that allows for the bypass of a COQ7 defect (see sect. 5.4 for additional details). Thus, the beneficial effects of 2,4-DHB treatment may indicate a deficiency of COQ7 activity in *Coq8b* KO cells. CoQ level was not determined in the kidney of *Nphs2-Cre;Coq8b^loxP/loxP^* mice; thus we do not know about the severity of CoQ deficiency in the affected podocytes and whether they accumulate DMQ (the substrate of COQ7).

##### 5.1.2.5. coq9 mouse mutants.

Two *Coq9* knockin models of different severity were described. *Coq9^R239X^* mice carry a homozygous truncation mutation that is a homolog to the human mutation R244X identified in the first *COQ9* patient reported (349, 518). Severe CoQ deficiency was found in all tissues tested, including cerebrum, cerebellum, heart, kidney, liver, and skeletal muscles, i.e., there was ≥80% reduction of CoQ levels in *Coq9^R239X^* tissues compared to wild type (349). *Coq9^R239X^* mice exhibit predominant encephalomyopathy as a phenotype, and in the brain significant impairment of mitochondrial respiratory function, neuronal death, and profound demyelination were found in the hindbrain area, resulting in early death between 3 and 6 mo of age. Similar to what was found in the skin fibroblasts from the COQ9 (R244X) patient, *Coq9^R239X^* tissues showed a severe reduction in COQ7 protein levels and accumulation of DMQ, the substrate of the COQ7 enzyme, indicating the dependence of full COQ7 expression level and activity on the presence of COQ9 (349, 518). In comparison, *Coq9^Q95X^* mice have higher residual CoQ levels than *Coq9^R239X^* mice. In the cerebrum and kidney, 40–50% residual CoQ_9_ levels were detected in *Coq9^Q95X^* mice, whereas the CoQ_9_ levels declined by 85–90% in the same tissues of *Coq9^R239X^* mice (349, 535). In muscle, however, CoQ levels are similar in the two models: both have 10–20% CoQ compared to their respective wild-type controls. Consistent with less severe loss of CoQ, *Coq9^Q95X^* mice have a much milder phenotype. The female mice, but not the males, showed signs of mild mitochondrial myopathy at 6 mo of age (535). Perturbation of the mitochondrial sulfide oxidation pathway was observed in both *Coq9^Q95X^* and *Coq9^R239X^* tissues, and the decrease in SQOR protein levels and activity appears to correlate with the severity of the CoQ loss (89). Finally, only *Coq9^R239X^* but not *Coq9^Q95X^* mice were found to respond to 2,4-DHB treatment, which is consistent with a more pronounced reduction of COQ7 protein levels and more severe loss of CoQ in *Coq9^R239X^* mutants (121, 535).

### 5.2. CoQ Deficiency in the Invertebrate Model C. elegans

Here we propose to review some studies in the *C. elegans* invertebrate model to give a flavor of the research on CoQ in the wider field of biology and because of the links of this research to the aging process and the ROS theory of aging. An exhaustive description of CoQ research in other invertebrates and plants would go beyond the scope of the present review.

The nematode *C. elegans* synthesizes CoQ_9_, but it also acquires CoQ_8_ from its bacterial food source (33, 35). Knockout mutations in *C. elegans* CoQ biosynthesis genes such as *coq-1, coq-2, coq-3,* and *coq-8* result in animals that can only survive for one generation on the normal bacterial food supply (*E. coli* strain OP50 that produce CoQ_8_) but whose progeny cannot develop normally and become fertile (36, 536–538). The mechanism by which the first homozygous generation can survive appears to be via sufficient gene product and CoQ being deposited in the egg by the heterozygous mother to last one generation, underscoring how very little CoQ is actually necessary for survival (539). The situation is different for mutants of the *clk-1* gene (the ortholog of yeast *COQ7*) (540). *clk-1*, which stands for clock, was identified in a forward genetic screen for mutants with slow and irregular development and behavior but a long life span (541). In fact, *clk-1* was one of the very first longevity genes ever identified and studied at the molecular level (540). Unlike other *C. elegans coq* deletion mutants, *clk-1* mutant strains can grow indefinitely on the normal bacterial food supply (the *E. coli* strain OP50) but cannot grow on bacteria that do not produce CoQ_8_ (35). In *clk-1* mutants, the biosynthetic intermediate demethylubiquinone-9 (DMQ_9_; FIGURE 15) accumulates instead of CoQ_9_ (33, 35). This strongly suggests that DMQ_9_ has functional properties that partially compensate for the loss of CoQ_9_, which allows these mutants to grow as long as they can obtain dietary CoQ_8_ (see also Ref. 83 for a study of the same situation in mammalian cells). However, how and where in the cell DMQ functions to allow survival is not yet known (see sect. 5.3). The properties of DMQ_9_ clearly cannot fully compensate for the loss of CoQ_9_, as *clk-1* mutants cannot grow without CoQ_8_ (32, 35, 542). On the other hand, although the presence of endogenously synthesized DMQ_9_ supplemented with dietary bacterial CoQ_8_ allows for viability, this cannot fully replace CoQ_9_ functionally, as the loss of *clk-1* produces a long list of complex phenotypes including slow embryonic and postembryonic growth rates, slow rhythmic behaviors, lower brood size, and increased life span (540, 543, 544). A particularly interesting type of phenotype shown by *clk-1* mutants is loss of normal acclimation to changes in temperature (543, 545). Interestingly, the life span of *clk-1* mutants has been reported to be unresponsive to another environmental parameter, failing to display life span changes in response to food availability (546).

All these phenotypic effects of *clk-1* mutations must result from the profound alteration in CoQ content of the mutants (no CoQ_9_, some CoQ_8_, and plenty of DMQ_9_). The *clk-1* phenotype, although resulting in lower oxygen mitochondrial respiratory chain function and a long life span, is quite different from the phenotype of other longevous mutants that affect mitochondrial function (544, 547, 548). Thus, it is likely that it is not low mitochondrial function per se that results in these complex phenotypes. Of course, as reviewed in sects. 3 and 4, CoQ has many other functions besides participating in energy generation. Any one of them could be involved in the complex *clk-1* phenotype. CoQ is also expected to be deeply involved in ROS biology, as reviewed in sect. 3, and ROS are signaling molecules that modulate numerous signal transduction pathways. The CLK-1 protein could also have additional functions besides CoQ biosynthesis, as has been suggested (549). However, this does not seem to be the case, as providing *clk-1* mutants with 2,4-DHB, which is an alternative, unnatural, biosynthetic precursor of CoQ synthesis (see sect. 5.4), fully rescues the *clk-1* mutant phenotypes in the total absence of the CLK-1 protein (550).

Finally, it is notable that a genetic screen for suppression of the slow growth phenotype of the *clk-1(e2519)* mutant, which is a partial loss of function allele with a glutamic acid-to-lysine substitution at position 148 of the protein, has identified several suppressor mutations. But despite the high number of genomes screened, all the identified suppressor mutations were mapped to tRNA^Glu^ genes whose anticodons were altered to read the substituted Lys codon of *clk-1(e2519)* (32). Furthermore, when the null mutation *qm30* was used in a similar screen, no suppressor was found. These findings strongly suggest that no genetic change in *C. elegans* can bypass the need for endogenously synthesized CoQ_9_. The irreplaceability of endogenously synthesized CoQ_9_ suggests that exogenous CoQ_8_ (from the diet) cannot reach all cells or subcellular locations where CoQ is needed, or the length of the side chain is more crucial than anticipated. But it also means that where CoQ_9_ is missing, no process can be genetically tweaked to compensate for the absence of CoQ_9_. Another insight that the study of *clk-1(e2519)* phenotype suppressors provided is that the effects of lacking CLK-1 and CoQ are cell autonomous. The suppressor mutations mapped to five distinct tRNA^GLU^ genes, and although the mutation in these genes were always the same (a C-to-T transition in the anticodon at position 36 of the gene), they suppressed different subsets of *clk-1* phenotypes to different degrees. This means that the different tRNA^GLU^ genes are expressed to different levels in different cell types and thus participate there more or less in the translation of proteins (here CLK-1). And the differences in the phenotypic rescue by the different suppressors mean very little, or no CoQ synthesized in any cell has the opportunity to move to another cell.

### 5.3. The CoQ Biosynthetic Intermediate DMQ

DMQ is the only late intermediate capable of accumulating in mutants with *COQ* gene defects. Its accumulation results from reduced activity of COQ7 (31, 33, 542, 551, 552). DMQ differs from CoQ only by missing one of the two methoxy groups (FIGURE 15). There have been considerations of its possible role in the phenotypes of *COQ7* mutants, by fulfilling (or interfering with) some of the functions of CoQ. In the nematode *C. elegans*, the *clk-1 (coq7* ortholog) mutant is the only *coq* mutant that can be maintained as a homozygous line (see sect. 5.2 for more information about *clk-1* worm mutants). All other *C. elegans* mutants completely devoid of endogenous CoQ or DMQ are not viable, even when feeding *E. coli* able to produce CoQ_8_, without which even *clk-1* mutants are not viable (35, 36). The large amount of DMQ_9_ present in *clk-1* mutants most likely determines the viability of the *clk-1* mutants with access to dietary CoQ_8_, but it is not yet clear how (33, 34). An in vitro study examining the effects of the addition of DMQ_9_ on CoQ-dependent electron transport activity by replenishing CoQ_9_-depleted mitochondria membranes with DMQ_9_ (prepared from *clk-1* worms) suggested that DMQ_9_ competes with CoQ in the respiratory chain of worms, specifically in the electron transport from CI to CIII (35). However, this competition is likely not seriously deleterious, as it was shown that the presence of DMQ does not contribute much, or anything, to the Clk-1 phenotype (32, 553). This is best demonstrated by missense tRNA suppressors of the *clk-1(e2519)* point mutation that restores endogenous CoQ_9_ biosynthesis only to a very small extent (but still allows for the accumulation of large amounts of DMQ_9_) yet leads to full rescue of the Clk-1 phenotype (32). Along these lines, as mentioned above, in liver-specific CoQ deficiency mouse models it was found that a large depletion of CoQ in hepatocytes causes only mild impairment of respiratory chain function for both *Pdss2* KO livers that make no DMQ and DMQ-accumulating *Coq7* KO livers (30, 82).

As mentioned above, total KO of *Coq7* in mice is embryonic lethal, similar to mutations in other CoQ biosynthetic enzymes (8, 29, 31, 522, 528). However, mouse embryonic fibroblasts in which COQ7 activity is absent are viable in vitro. They lack CoQ_9_ and show an accumulation of DMQ_9_ (31, 82). The viability of *Coq7* KO cells under in vitro culture conditions is in fact due to the minimal amount of CoQ_10_ present in the normal culture media (83), similar in this way to *C. elegans clk-1* mutants that require bacterial CoQ_8_ to survive. However, although mitochondrial oxidative phosphorylation was compromised in *Coq7* KO MEFs, it was to a much lesser extent compared to *Coq7/Pdss2* double-KO cells that have neither CoQ nor DMQ (83). This demonstrates that DMQ is actually capable of some respiratory electron transport. However, the fact that *Coq7* KO cells, like other ETC mutants, cannot grow in the respiration-dependent (galactose) medium and their lethality in galactose can be rescued by provision of exogenous CoQ indicates that DMQ is a much less efficient electron transporter than CoQ and that the C6-methoxy group in the CoQ ring is crucial for its electron transport function in the respiratory chain (82, 83).

Another possible function of DMQ that has been considered is to act as an antioxidant. This could explain why oxidative stress is not elevated in *Coq7* mouse KO and the expression of antioxidant enzymes is low (123). However, so far this has not been thoroughly tested. In yeast cells, DMQ_6_ was shown not to be effective in protecting against oxidative stress generated by H_2_O_2_ or PUFA linolenic acid (554). In nematode, it was shown that DMQ_9_ present in the plasma membrane is unlikely to be active in redox reactions (555).

### 5.4. Chemical Analogs of 4-HB That Can Bypass Deficiencies in Some Steps of CoQ Biosynthesis

**Figure 16.**
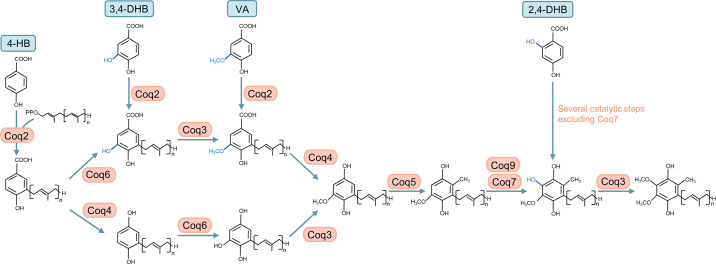
The chemical structure of 2,4-dihydroxybenzoic acid (2,4-DHB), 3,4-dihydroxybenzoic acid (3,4-DHB), and vanillic acid (VA) and their use as alternative benzoquinone ring precursors for CoQ biosynthesis. Of note, as mentioned in FIGURE 15. the order of Coq3-, Coq4-, and Coq6-catalyzed reactions has not been clearly established. A representative scheme of their use in the CoQ biosynthetic pathway is shown. However, it is possible that different sequences of reactions coexist or that the sequence of reactions changes depending on the availability of different precursors. See glossary for other abbreviations.

Some structural analogs of 4-HB, specifically vanillic acid (VA), 3,4-dihydroxybenzoate (3,4-DHB), and 2,4-dihydroxybenzoate (2,4-DHB), were first shown in yeast to be usable as alternative CoQ biosynthetic precursors (484, 489). Because their aromatic part already carries the chemical groups that are normally added by specific enzymes, such as COQ6 and COQ7, their use allows for bypass of the specific synthesis blocks due to mutations in these enzymes and thereby enables endogenous CoQ biosynthesis despite the absence of the enzymes (FIGURE 16). In yeast, this requires that the biosynthetic CoQ complex can still form, which can be achieved by overexpressing *COQ8* or by expressing a stable yet inactive mutant enzyme to prevent a complete collapse of the complex. In the case of *COQ6*, it was shown in yeast Δ*coq6* cells expressing enzymatic inactive but structurally stable yeast or human COQ6 protein that treatment with VA or 3,4-DHB leads to a partial restoration of endogenous CoQ_6_ biosynthesis (489, 556). Another hydroxylated form of 4-HB, 2,4-DHB, differs from 4-HB by already having a hydroxyl group at the position of the aromatic ring that COQ7 normally hydroxylates, thereby obviating the need for Coq7 for CoQ_6_ biosynthesis (484).

These findings have been verified in metazoans (123, 479, 550, 557). For example, VA is shown to increase CoQ_10_ production in a CRISPR *COQ6* KO in human HEK293 cells (558), and 2,4-DHB has been shown to successfully restore CoQ biosynthesis in mouse and human cells with partial or total loss of *COQ7* (112, 115, 123). Importantly, 2,4-DHB administration has been demonstrated to reverse disease phenotypes in *C. elegans clk-1* mutant, conditional *Coq7* knockout (KO) mice, and a *Coq9* mouse model with particularly low COQ7 activity (*Coq9^R239X^*) (121, 123, 559). But 2,4-DHB does not show a CoQ-increasing effect in all conditions in which there is a partial loss of COQ7 (99, 115). We postulate that in mammalian cells in which a loss of COQ7 does not result in a complete loss of the biosynthetic CoQ complex, 2,4-DHB likely would benefit only very severe CoQ deficiency in which there is very little remaining COQ7 activity. This is because if there is a residual native CoQ biosynthetic pathway using 4-HB as the aromatic ring precursor, this residual pathway would compete for the same CoQ pathway enzymes (the other enzymes in the pathway besides COQ7). So, treatment of COQ7 mutants with 2,4-DHB does not necessarily result in increased CoQ production because lower CoQ production from 4-HB might offset the gain from CoQ produced via 2,4-DHB. The observed lesser accumulation of DMQ in the mutant cells and a decrease of CoQ in normal cells after 2,4-DHB treatment are consistent with this model (112, 115, 123). Future studies are needed to understand the mechanisms at play to be able to explore the possibility of using 2,4-DHB as a treatment for certain types of CoQ deficiency.

## 6. HUMAN PRIMARY CoQ_10_ DEFICIENCY

### 6.1. Human Patients with Primary CoQ_10_ Deficiency

In animals, CoQ is synthesized in all tissues, and dietary intake is not known to have any impact. The presence of CoQ in the blood reflects mostly the amount carried in lipoproteins, not intracellular levels in the cells of solid tissues. Primary CoQ_10_ deficiency is a clinically heterogeneous and rare disorder that is caused by mutations in genes implicated in the CoQ_10_ biosynthesis pathway (11, 26, 27, 39, 460). The first case of CoQ deficiency was described in 1989 (560). In the last few decades, with the increasing availability and affordability of genomic sequencing technology, whole genome or exome sequencing is increasingly becoming the first-line diagnostic test for patients suspected of having genetic disorders, including primary CoQ deficiency. This has accelerated the discovery of novel primary CoQ deficiency disease variants. The prevalence of primary CoQ_10_ deficiency was conservatively estimated to be a total of 123,789 individuals worldwide and 1,462 in the United States (561). Disease-causing mutations have now been reported for *PDSS1*, *PDSS2*, *COQ2*, *COQ4*, *COQ5*, *COQ6*, *COQ7*, *COQ8A*, *COQ8B*, and *COQ9* genes, with a total of 383 patients from 276 families reported so far in the literature (TABLE 5) (38–40, 562).

**Table 5.** Primary CoQ_10_ deficiency patients reported in the literature

Gene	No. of Patients (no. of Families)	No. of Pathogenic Variants	Age of Onset (range)	Common Clinical Manifestations
*PDSS1*	3 (2)	3	Infancy to 2 yr	Encephalopathy, development delay, SRNS, SND
*PDSS2*	7 (5)	5	Infancy to 2 yr	Kidney disease mainly SRNS, ataxia, SND
*COQ2*	31 (23)	23	Infancy to 68 yr	Kidney disease mainly SRNS, encephalopathy
*COQ4*	35 (26)	22	Infancy to 9 yr	Encephalopathy, development delay, seizure, hypotonia, cardiomyopathy
*COQ5*	5 (3)	3	Infancy to childhood	Ataxia, dysarthria, encephalopathy, development delay
*COQ6*	33 (24)	16	2 mo to 16 yr	SRNS, SND
*COQ7*	32 (25)	9	Infancy to 15 yr	Spasticity, limb weakness, hypotonia, neuropathy, difficulty walking, hearing loss
*COQ8A*	133 (104)	89	Infancy to 75 yr	Cerebellar ataxia, development delay, muscular symptoms
*COQ8B*	97 (60)	38	10 days to 32 yr	Kidney disease, mainly SRNS
*COQ9*	7 (4)	5	Infancy to 9 mo	Encephalopathy, seizure, renal tubulopathy, development delay

NS, nephrotic syndrome; SND, sensorineural deafness; SRNS, steroid-resistant nephrotic syndrome. Infancy: 0–1 yr. For references, see sect. 6.1.

Generally speaking, typical primary CoQ_10_ deficiency patients present symptoms that resemble those of inborn mitochondrial respiratory chain disorders, including early onset, multiorgan involvement, and prevalent neurological and muscular manifestations. However, there is great heterogeneity in the clinical manifestations of CoQ deficiency, which is not fully understood. Nonetheless, mutations disrupting an enzymatic activity in the CoQ biosynthetic pathway or a key organizer of the CoQ biosynthetic complex lead more frequently than not to severe disease outcomes. Fatal infantile multisystem disease has been reported for *PDSS2*, *COQ2*, *COQ4*, *COQ7*, and *COQ9* (111, 115, 518, 563–565). However, in the majority of patients, primary CoQ_10_ deficiency affects only a few organs or tissues. Mutations in *PDSS2*, *COQ2*, *COQ6*, and *COQ8B*/*ADCK4* are frequently associated with glomerular disease, mainly steroid-resistant nephrotic syndrome (SRNS) (534, 566, 567). Most SRNS cases are accompanied by focal segmental glomerulosclerosis (FSGS) and are characterized by childhood onset of proteinuria and a high risk of progression to kidney failure. To date, *PDSS2* mutations have been identified in seven patients, and all of them have nephrotic syndrome (NS) presenting at a young age. For most of them (6/7) other organs are also affected (407, 563, 568–570). Twenty-two out of 31 identified *COQ2* patients present with renal dysfunction (mainly SRNS). In most of these cases (17/22) renal symptoms had already occurred within the first 2.5 yr of life, and 14 showed no sign of extrarenal involvement (81, 516, 564, 565, 571–581). Out of a total of 34 identified *COQ6* patients, 29 (≈85%) have renal manifestations, mainly isolated SRNS, and about one-third (≈35%) have sensorineural deafness with or without a renal phenotype (408, 569, 577, 582–588). Of the 100 *COQ8B/ADCK4* patients who have been described, almost all present with renal dysfunction, with SRNS being the most frequent disease manifestation (36/97). Thus, mutations in the *COQ8B/ADCK4* gene account for the highest number of primary CoQ_10_ deficiency patients with kidney disease. Extrarenal symptoms were scarce in *COQ8B/ADCK4* patients, and their symptoms are relatively milder, mostly likely owing to less disruption of the CoQ_10_ biosynthetic machinery and a selective glomerular phenotype (534, 566, 567). The kidney focus of the phenotype suggests that COQ8B/ADCK4 might be particularly limiting for CoQ_10_ production in the kidney. But it is unknown whether the pathogenic variants of *COQ8B* actually cause a more severe CoQ deficiency in the kidney, in particular in the glomeruli.

Cerebellar ataxia is one of the most common presentations of primary CoQ_10_ deficiency, which has been considered to reflect a higher susceptibility of cerebellar Purkinje cells to CoQ deficiency and mitochondrial dysfunction. Ataxia is not observed in patients with *COQ8B* mutations, but the dominant clinical feature associated with *COQ8A/ADCK3* mutations is progressive cerebellar ataxia, variably combined with muscular and other neurological symptoms. *COQ8A*-associated ataxia is also known as autosomal recessive cerebellar ataxia 2 (ARCA2) or autosomal recessive spinocerebellar ataxia (SCAR9). Overall, it is highly heterogeneous in terms of age at onset and degree of severity, ranging from severe childhood-onset to milder adult-onset forms. In the available literature, we found 133 patients reported to harbor *COQ8A/ADCK3* mutations, and all of them suffer from cerebellar ataxia of varying degrees of severity (589–620). It should be noted, however, that, because of the known high prevalence of ataxia in *COQ8A/ADCK3* patients, individuals with unexplained ataxia are more likely to be tested for mutations in *COQ8A* than in other *COQ* genes. Besides *COQ8A*, all four *COQ5* patients for whom clinical data are available are also found to show a cerebellar ataxic phenotype (621, 622).

*COQ4* patients often present with a broad spectrum of symptoms, and most (≈83%, 29/35) have an age of onset of <8 mo of age. About half of the patient cases (14/35) were reported to have poor outcomes, with death within 2 yr of life (111, 114, 623–628). The high clinical severity of mutations in the *COQ4* gene is consistent with its predicted central role in the physical organization of the CoQ biosynthetic complex. Mutations in *PDSS1* and *COQ9* have been reported in a few numbers of patients, with variable phenotypes ranging from infancy-onset multisystemic disorder to a milder disease (516, 518, 629–632). Interestingly, *COQ7* patients appear to frequently suffer from neuropathy and muscular disorders such as spasticity, limb weakness, pure motor neuropathy, and difficulty in walking (99, 112, 115, 357, 633–639). The mechanism that underlies the high frequency of motor axonal damage in *COQ7* patients is unknown.

Variable tissue- or cell type-specific expression levels of *COQ* genes, varying degrees of pathogenicity of different mutations, and differences in tissue requirement for CoQ are the likely causes of the high variability of disease presentation and severity of primary CoQ_10_ deficiency. For example, unlike other tissues, glomerular podocytes are shown to express *COQ8B* at high levels but their expression of *COQ8A* is very low (566). This could influence the largely kidney-limited phenotypes of *COQ8B* patients. It also remains to be clarified whether DMQ accumulation in *COQ7* mutants has any role in the phenotypes that distinguish *COQ7* patients (83). It is worth noting that, in contrast to yeast *coq7* null mutants, mammalian cells and tissues accumulate DMQ in the complete absence of COQ7 and show no significant loss of other COQ proteins, indicating possible differences in the constituent proteins or assembly control of the CoQ biosynthetic complex between phyla (82, 115, 123).

### 6.2. Treatment of Primary CoQ_10_ Deficiency

After diagnosis, primary CoQ_10_ deficiency patients are immediately given oral CoQ_10_ as replacement therapy. Exogenous CoQ_10_ supplementation is the only treatment option currently available for CoQ deficiency and, to the best of our knowledge, is only available in an oral formulation (640). Its effectiveness, however, is highly controversial (38–40). Overall, in the cases where a positive response to CoQ_10_ supplementation treatment was described, the reported responses were very partial improvement of only some symptoms, and the reports, which necessarily lack controls, are often plagued by a lack of details and follow-up (40). On the other hand, animal studies suggest that most phenotypes due to severe CoQ deficiency can be completely rescued by a partial restoration of endogenous CoQ biosynthesis (121, 123). Hence, there is a need to develop alternative strategies for providing CoQ_10_ or different treatment strategies that act by boosting the residual activity of the CoQ biosynthetic pathway. For some specific primary forms of CoQ deficiency, that are due to mutations in *COQ6*, *COQ7,* or *COQ8B*, modified precursors of the quinone ring of CoQ_10_, for example, 2,4-dihydroxybenzoic acid (2,4-DHB), have been considered as a potential alternative treatment option (115, 123, 489, 522, 530, 535, 556). As mentioned in sect. 5.4, these molecules, by providing the lacking chemical group on the quinone ring due to defects in specific COQ enzymatic activities, allow the restoration of CoQ biosynthesis in the cells lacking a specific *COQ* gene product (484). However, further work is needed to explore and clarify the possibilities of their use in treating primary CoQ deficiency, especially given that the patient cells still always retain some degree of residual activity of the affected proteins, and that this residual activity can be inhibited by the alternative pathway triggered by the presence of alternative precursors (99, 115).

## 7. SECONDARY CoQ DEFICIENCY

**Figure 17.**
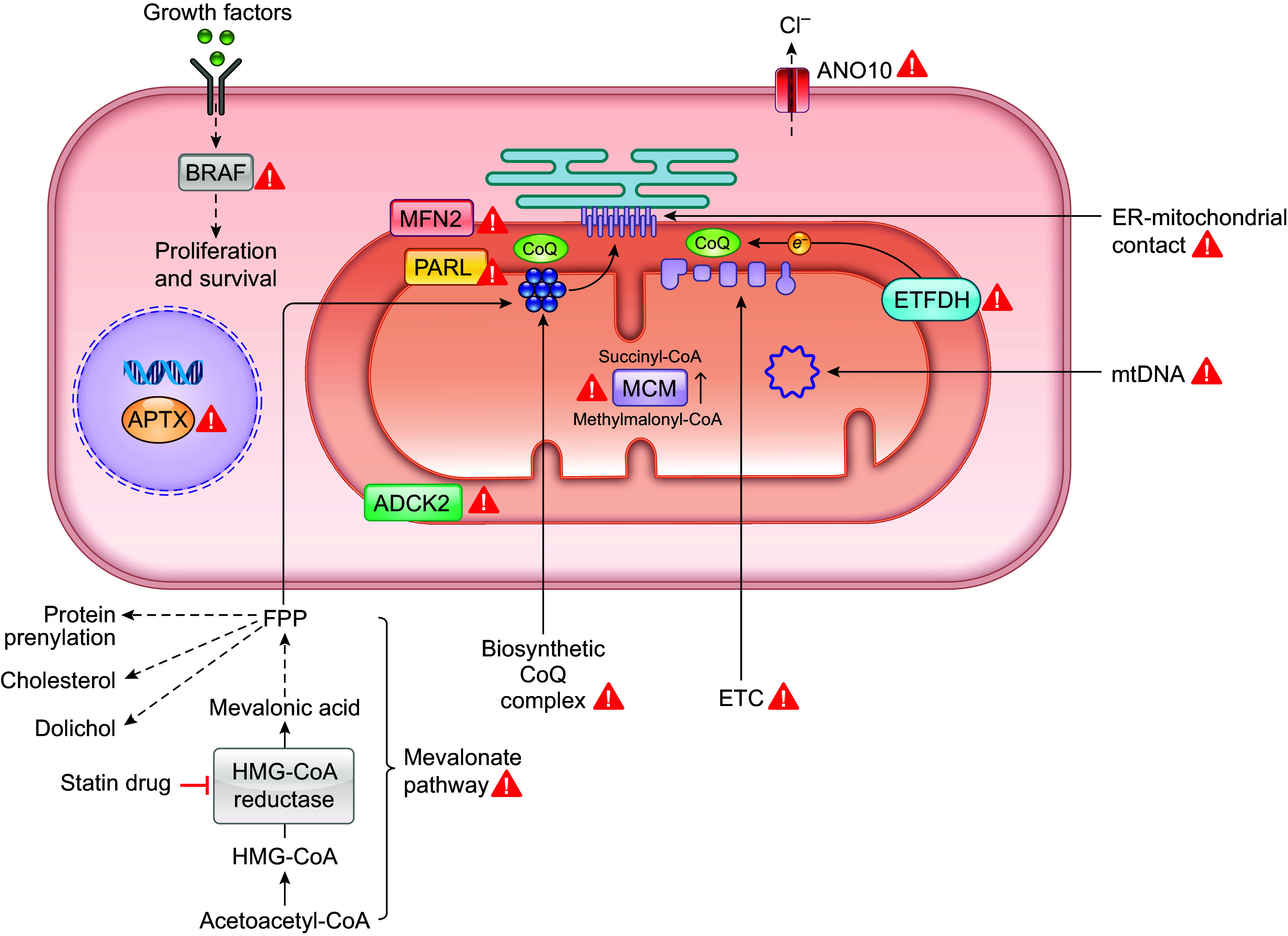
Gene products and pathways whose defects are shown to cause secondary CoQ deficiency. See text for details. See glossary for abbreviations.

Secondary CoQ deficiencies caused by defects that are not directly related to the CoQ synthesis machinery have been reported to be associated with various diseases (641, 642). Moreover, we can expect that more cases will be reported with the increasing awareness of the existence of such secondary deficiencies and their potential role as aggravating factors in disease pathophysiology. Below, we list and summarize secondary CoQ deficiencies described in the available literature (FIGURE 17). We have grouped them based on the link between the primary cause of disease and the cellular CoQ biosynthetic machinery.

### 7.1. CoQ Deficiency Induced by Inhibition of the Mevalonate Pathway

The most well-known condition associated with secondary CoQ deficiency is the use of the cholesterol-lowering drug statin. The function of statin drugs is to inhibit 3-hydroxy-3-methylglutaryl coenzyme A (HMG-CoA) reductase, a rate-limiting enzyme of the mevalonate pathway that synthesizes farnesyl pyrophosphate (FPP) as an intermediate. FPP is a direct precursor of squalene, an isoprenoid containing six isoprene subunits that is the precursor of cholesterol biosynthesis. But FPP is also the source of the isoprenoid side chain of CoQ and a substrate for a number of other biosynthetic processes including the biosynthesis of dolichol and isoprenylated hemes and proteins (643). Therefore, inhibition of the mevalonate pathway that leads to reduced FPP production likely inhibits CoQ biosynthesis as well, in addition to lowering cholesterol levels. However, it is not completely understood how the metabolic flux of FPP toward the biosynthesis of different isoprenoids is regulated. According to the flow diversion hypothesis, the regulation is mediated by the different affinities of the branch-point enzymes for FPP. This predicts that the first committed enzyme of cholesterol synthesis (squalene synthase) should have a lower affinity for FPP compared to the branch-point enzymes involved in CoQ and dolichol synthesis. This, in turn, would mean that an alteration of the FPP level would mainly affect the synthesis of cholesterol since the other branch-point enzymes are saturated even at low substrate concentrations (643).

Nevertheless, since its introduction to the market decades ago, statin therapy has been reported to lower blood CoQ_10_ levels (644–649). However, notably, one randomized crossover study failed to find any decrease in blood CoQ_10_ levels in healthy volunteers (650). Animal studies reported similar findings of reduced blood levels of CoQ after statin treatment (651, 652). However, the blood CoQ levels are thought to be mainly determined by circulating lipid levels, especially lipoproteins. Thus, a change in blood CoQ level may not reflect the CoQ biosynthetic status in tissues but only inform on the liver, where CoQ is incorporated into lipoprotein particles. In fact, in some studies a parallel decrease in CoQ and blood lipids was observed (648, 653).

One of the most well-known adverse effects of statin drugs is myopathy. The reported incidence of statin-induced myopathy ranges from 0.1–0.2% to as high as 25% (654, 655). The mechanism of statin-induced myotoxicity is not fully understood. CoQ deficiency, possibly resulting from reduced availability of FPP for the synthesis of the isoprenoid side chain of CoQ, has been hypothesized to be a possible cause. However, it is unclear whether statin therapy actually reduces CoQ in muscles. Among the few studies that have examined the muscle levels of CoQ_10_ in patients receiving statin treatment, one reported an ≈34% decrease of muscle CoQ_10_ levels in patients treated with simvastatin, but no significant effect, or even an increase, in skeletal muscle CoQ_10_ concentrations, was reported in three other studies, including one where the CoQ_10_ level was examined in patients with statin-associated myopathy (656–659). Another recent randomized trial with 37 patients taking simvastatin found no change in muscle CoQ_10_ levels after 8 wk of supplementation with CoQ_10_, despite a 4.8-fold increase in plasma CoQ_10_ concentration (660). The data from animal studies are also inconsistent (661–663). Impaired mitochondrial function, the primary consequence expected from CoQ depletion, has also not been unambiguously shown to occur (657, 658, 664–666). Furthermore, there is currently a lack of concrete evidence to support the beneficial effects of oral CoQ_10_ supplementation on the myopathic symptoms of statin users (see sect. 7.5). In conclusion, the effect of statins on CoQ levels and their potential role in statin myopathy remain to be convincingly demonstrated.

### 7.2. Secondary CoQ Deficiency in Mitochondrial Disorders

Mitochondrial disorders have been most frequently reported to be associated with CoQ deficiency. Low levels of CoQ have been described in muscle biopsies or cultured fibroblasts from patients with various types of mitochondrial defects, especially those involving mitochondrial DNA (mtDNA) mutations or depletion. A study involving 39 patients with a clinical phenotype suggestive of mitochondrial DNA depletion syndromes (MDS) showed that 75% of patients with mtDNA depletion presented with a decreased level of muscle CoQ but only 21% of patients with other mitochondrial disorders showed decreased muscle CoQ levels (667). A study including 72 cases showed that 44% of OXPHOS disorders (*n* = 44) displayed different degrees of CoQ deficiency in either their muscle or their fibroblasts. However, similar rates of low CoQ values were detected in the non-OXPHOS disorder group that includes 11 mitochondrial disease patients and 17 patients with diagnoses of other nonmitochondrial diseases (668). In the same vein, a high frequency of CoQ_10_ deficiency (28 over 76 patients) was reported to be present in patients presenting mitochondrial myopathy. However, no clear correlation was found between clinical muscle phenotypes (such as weakness and exercise intolerance) and the severity of CoQ deficiency in the muscle (669). Furthermore, fibroblast cultures obtained from two patients with mitochondrial encephalomyopathy, lactic acidosis, and strokelike episodes (MELAS) were reported to have an ≈40% reduction in CoQ_10_ levels. MELAS is a mitochondrial disorder caused by pathogenic variants in mtDNA (670). These two patients carry an A-to-G mutation at nucleotide 3243 in the tRNALeu^(UUR)^ gene, which inhibits mitochondrial synthesis and predominantly results in CI defect. A more recent study that analyzed CoQ_10_ levels in muscle mitochondria from 118 patients with mitochondrial disease of a variety of genetic etiologies found low levels (2 standard derivations below the normal mean) in about one-third (31%) of the samples (671).

According to the current model, CoQ is produced in the IMM by a supramolecular protein complex on the matrix side, the biosynthetic CoQ complex (see sect. 5.1.1). Moreover, yeast studies suggest that the biosynthetic CoQ complexes cluster into discrete foci (called CoQ domains) and are positioned adjacent to the ER-mitochondria contact sites. In yeast, loss of the ER-mitochondria encounter structure (ERMES) complex that tethers the ER to the mitochondria causes a disruption of the biosynthetic CoQ complex and impairs CoQ production (474, 502, 672). The IMM has an intricate ultrastructure. It is highly folded (into cristae) to create more surface area for chemical reactions to occur. Mitochondria and IMM cristae are also believed to constantly change in shape and volume in response to the metabolic state of the cell. Thus, it is reasonable to speculate that reduced respiratory chain activity and alterations in the IMM’s size, ultrastructure, or other properties, which are common consequences of mitochondrial disorders, could perturb the formation of the biosynthetic CoQ complex and its proper positioning in the IMM and thus CoQ production. In fact, abnormal mitochondria and crista morphology are common features of mitochondrial disorders (673). Mitochondrial dysfunction is also often associated with the accumulation of abnormal mitochondria or decreased mitochondrial mass. CoQ occurs in all cellular membranes, but its concentration is highest in the mitochondria. Therefore, lower cellular steady-state levels of CoQ also reflect changes in mitochondria volume when CoQ is measured in total cell or tissue lysate and normalized to total proteins, as is often the case in most CoQ assays. Nevertheless, implied consequences on cellular health, especially on energy production, are probably not less significant even if a secondary CoQ deficiency state mainly stems from mitochondrial loss. However, it is important to distinguish the two conditions, because different treatment strategies might be necessary.

Model animal studies provide direct evidence in support of the connection between mitochondrial defects and secondary CoQ deficiency. In a series of heart conditional KO models targeting essential factors required for mtDNA gene expression (*Twnk*, *Tfam*, *Polrmt*, *Lrpprc*, *Mterf4*), leading to OXPHOS dysfunction, a profound decrease of CoQ levels was observed as a result of a significant decrease in levels of CoQ biosynthetic pathway components (COQ3, COQ5, COQ6, COQ7, COQ8A, COQ9, and COQ10A) suggestive of disruption of the biosynthetic CoQ complex (674). Interestingly, the regulator components COQ8A and COQ8B showed strong opposite responses at both transcript and protein levels, indicating that they may be reciprocally regulated, but how, by what mechanism, and for what purpose is not known (674). Of note, this molecular response was not noted in other mouse models with mitochondrial dysfunction (675). Ablation of mitofusin2 (MFN2), an OMM GTPase that mediates outer membrane fusion as well as ER-mitochondria tethering, also causes a significant decrease of the levels of CoQ (676, 677). This was shown in the mouse heart and MEFs. No change in the levels of CoQ biosynthetic enzymes was found, but instead proteomic and metabolomics analyses showed a downregulation of the cytosolic mevalonate pathway (676). Cardiomyocytes isolated from *Mfn2* knockout hearts showed mitochondrial morphological heterogeneity with the appearance of enlarged mitochondria. It remains an interesting possibility that structural alterations of ER-mitochondria contact sites that lead to aberrant assembly or organization of CoQ biosynthetic domains are causative, at least partially, of the effect of *Mfn2* deletion on CoQ levels.

Presenilin-associated rhomboid-like (PARL) is a protease located in the IMM and plays an essential role in mitochondrial homeostasis (678). A significant reduction of CoQ levels was reported in the brain and testis of *Parl^−/−^* mice without changes in mitochondrial mass (675, 679). The mitochondria in *Parl^−/−^* brain or spermatocytes also showed severe and progressive ultrastructural abnormalities (swollen mitochondria with crista malformations and loss of matrix density) and assembly alterations in multiple respiratory chain complexes and, in consequence, impaired mitochondrial respiration (675, 679). Increased CoQH_2_-to-CoQ ratio was also observed, which is believed to be due to CIII dysfunction caused by TTC19 depletion (675, 679). TTC19 is a mitochondrial protein, embedded in the IMM, and is important for correct CIII assembly (680). In addition to TTC19, a downregulation of several CoQ biosynthetic proteins (COQ3, COQ5, COQ6, COQ7, COQ9) and SQOR was observed by a mitochondrial proteomic analysis of *Parl^−/−^* brain (675). Among these, the disruption in the COQ4 levels was the most prominent (675, 679). Given the severe mitochondrial morphological changes in the *Parl^−/−^* neurons, it is attempting to speculate that the mitochondrial structural defect results in the observed secondary CoQ biosynthesis defect. Notably, *Parl* ablation in skeletal muscle decreased COQ4, but it was not associated with a reduced level of CoQ (675). The apparent tissue-specific effect of PARL loss is not understood.

Mammalian mitochondria contain five atypical aarF domain containing kinase (ADCK) kinases (681). ADCK3 (COQ8A) and ADCK4 (COQ8B) have been identified as the orthologs of the yeast protein Coq8 that are required for CoQ biosynthesis (see sect. 5.1). A heterozygous nonsense mutation in *ADCK2* that led to severe myopathy and liver dysfunction has been identified in a human patient with histological signs of mitochondrial myopathy associated with lipid storage in skeletal muscle (117). An *Adck2* knockout mouse model was generated to understand the pathogenesis resulting from the loss of *ADCK2*. *Adck2^−/−^* mice are embryonically lethal, whereas heterozygous inactivation was shown to lead to mitochondrial myopathy in skeletal muscle and impaired fatty acid β-oxidation, recapitulating the phenotype of a human patient (117). A mild to moderate decrease of CoQ levels as well as CoQ biosynthesis rate, accompanied by impaired CoQ-dependent respiratory activities, were found in the MEFs and skeletal muscle (not in the brain, liver, heart, and kidney) of *Adck2^+/−^* mice. CoQ deficiency, though not very severe, is proposed to be causative of the *Adck2^+/−^* mouse myopathy and liver dysfunction, based on the observation that both the human patient and the *Adck2^+/−^* mouse mutant appear to respond to CoQ_10_ treatment (117). The cause of CoQ deficiency in *Adck2*-deficient cells is not understood. It is thought, however, to be secondary to defective lipid transport into mitochondria (117).

### 7.3. Secondary CoQ Deficiency in Cerebellar Ataxia

*ANO10* encodes anoctamin-10, a member of a family of putative calcium-activated chloride channels. Lower CoQ_10_ levels in muscle were reported in two patients with adult-onset cerebellar ataxia who carry *ANO10* mutations (682). Of note, skin and muscle biopsies are the only readily accessible tissue samples for CoQ measurement in human patients. For genetic diseases, low levels of muscle CoQ are used to suggest whether other organs, such as the cerebellum, might have similar CoQ deficits. It has been postulated that cerebellar ataxia in patients with ANO10 deficiency may be due to deranged calcium signaling in Purkinje cells (683). Secondary CoQ deficiency was also described in association with mutations in the *ATPX* gene, which encodes the DNA strand-break repair protein aprataxin (APTX) (684–687). *APTX* mutations cause ataxia with oculomotor apraxia type 1, a neurodegenerative disorder with early-onset cerebellar ataxia, oculomotor apraxia, and severe axonal polyneuropathy. A lower-than-normal CoQ_10_ level was demonstrated in the muscle and fibroblasts of *APTX* patients. APTX functions in both nuclear and mitochondrial DNA maintenance (688). In vitro studies described mitochondrial functional and morphological changes in ATPX-depleted human U2OS cells, such as fragmentation and reduced crista density (689). However, morphology appears to be normal in HeLa cells after shRNA knockdown of *APTX*, which resulted in a 76% reduction in APTX protein levels and a 29% reduction in CoQ_10_ level (687). Two separate studies reported a decrease of CoQ_10_ levels in the cerebellum of multiple system atrophy (MSA) patients in comparison with normal control subjects and other neurodegenerative diseases (715, 716). Studies also reported reduced protein expression of PDSS1 and COQ5 and decreased *COQ2* and *COQ7* expression in disease-affected regions of the MSA patients (717). It should be mentioned that the samples used for analyses were collected postmortem. MSA is an adult-onset, fatal neurodegenerative disease characterized by a combination of motor abnormalities (parkinsonism and ataxia are the 2 main types) and symptoms that affect the involuntary (autonomic) nervous system. The neuronal loss in the affected area (autonomic centers, basal ganglia, and cerebellar circuits) is often accompanied by oligodendrocytic accumulation of α-synuclein. However, the etiology of MSA is largely unknown. A study with neural progenitors derived from induced pluripotent stem cells (iPSCs) of MSA patients showed mitochondrial morphology changes toward a more tubulated phenotype in MSA cells, but CoQ levels were not explored (690). Ataxia is one of the most common clinical phenotypes associated with CoQ_10_ deficiency. Reduced CoQ_10_ levels may act as a separate pathogenic effector causing ataxia in these genetic or acquired conditions associated with ataxia.

### 7.4. Secondary CoQ Deficiency in Other Conditions

The genetic conditions that have been linked to CoQ deficiency include mutations in *ETFDH* and *BRAF.* As mentioned in sect. 2.2, ETFDH, located in the IMM, mediates electron transport from flavoprotein dehydrogenases to the CoQ pool. ETFDH defects are often associated with impaired fatty acid oxidation. One study reported CoQ deficiency in the muscles of seven patients with *ETDFH* mutations presenting isolated myopathy (691), but a few later studies did not always find CoQ deficiency in other patients carrying *ETFDH* mutations (692, 693). A single patient with cardiofaciocutaneous syndrome due to a *BRAF* gene mutation was shown to have a muscular CoQ_10_ deficiency (694). To the best of our knowledge, no other case has been reported.

Fibroblasts from patients with methylmalonic acidemia due to a deficit of methylmalonyl-CoA mutase (MCM or MUT) activity were also reported to have CoQ_10_ contents below normal values. However, the CoQ content measured in a muscle sample from one of the patients was not affected. Methylmalonic acidemia is an organic acidemia caused by a deficiency of the mitochondrial enzyme MCM or its cofactor cyanocobalamin, or in rare cases by a deficient activity of methylmalonyl-CoA epimerase (MCE). MCM is involved in the catabolism of propionyl-CoA, a degradation product of cholesterol, branched-chain amino acids, and the β-oxidation of odd-chain fatty acids, converting L-methylmalonyl-CoA to succinyl-CoA, which then enters the TCA cycle (695). Methylmalonic acidemia is characterized by methylmalonic acid (the metabolite of methylmalonyl-CoA) accumulation in tissues and body fluids, and severe metabolic acidosis, neurological symptoms (such as mental impairment), and kidney failure are among the severe consequences of this disease. A further study reported a decrease in renal CoQ_10_ content in an *MCM* knockout mouse model (696). However, the level of the dominant CoQ species, CoQ_9_, was comparable to the control level, pointing to a strange CoQ isoform-specific effect.

Finally, CoQ levels were reported to decline with age, and some age-dependent diseases were reported to be associated with secondary CoQ deficiency, which could be a risk factor in disease development or progression (697). For example, decreased levels of CoQ were reported in the brains of Parkinson disease patients (698). The potential factors responsible for eliciting the decrease in cellular CoQ levels with age could include age-related decline of mitochondrial content and functional integrity, increased CoQ demand (e.g., for higher antioxidant protection capacity), and poor nutritional status.

### 7.5. Treatment of Secondary CoQ Deficiency

There are two crucial questions that need to be answered about secondary CoQ deficiencies: what are the underlying mechanisms leading to the deficiencies and do these secondary deficiencies add to the pathophysiology of the diseases in which they are observed? Answering these questions is especially challenging because the relevant diseases are highly heterogeneous. A lack of reliable methods for CoQ supplementation also presents an impediment to addressing the latter question (see sect. 3.4). Moreover, it is worth remarking that, given the difficulty of obtaining appropriate controls, it is difficult to establish whether the levels of CoQ in a patient are actually abnormally low. Nevertheless, given the high prevalence of some of these diseases, it is important to understand what role CoQ plays. For example, more than 200 million people around the world are estimated to take cholesterol-lowering statin drugs, and the prevalence of mitochondrial disease has been estimated at ∼1/5,000 live births worldwide (699, 700).

Whatever the underlying mechanisms for secondary deficiency may be, the implication for clinical practice is that diseases that present it could benefit from CoQ supplementation. Indeed, a number of studies, including clinical trials, have reported the effects of CoQ_10_ supplementation in a number of diseases. However, overall, the results are quite variable. For example, one meta-analysis of randomized controlled trials concluded that CoQ_10_ supplementation can ameliorate statin-associated muscle symptoms (SAMS), whereas two other similar studies did not succeed in demonstrating that CoQ_10_ supplementation was beneficial for patients with SAMS (701–703). One of the early randomized clinical trials in patients with mitochondrial diseases reported a 5.5-fold increase of plasma CoQ_10_ levels after 60 days of CoQ_10_ treatment (1,200 mg/day), and this was associated with a slight increase in exercise aerobic capacity (Vo_2_/kg lean mass after 5 min of cycling) and an attenuation of postexercise rise in lactate, whereas no effects of supplementation were observed on other clinically relevant variables such as forearm grip strength (370). Furthermore, a phase III trial of CoQ_10_ in children with a deficiency of ETC complexes or a molecular diagnosis of mitochondrial disorder reported that receiving CoQ_10_ at 10–400 mg/kg daily for 6 mo made no significant difference in the two primary outcome measures: McMaster gross motor function and pediatric quality of life scales (https://clinicaltrials.gov/ct2/show/NCT00432744). For further details on this topic, interested readers can consult references such as Refs. 43, 649, 651, 704–709.

Supplementation with CoQ_10_ is arguably effective in some cases, but the reported effects are often of a very small magnitude. Assessment of the actual effect on tissue CoQ levels, except for the plasma and muscle, cannot routinely be performed on human patients. Therefore, it is not known whether any beneficial effect stems from an elevation of CoQ level at its key sites of action, including mitochondria, as is generally presumed, or even whether the observed effect correlates with restoration of CoQ levels in the affected tissues. Another obvious difficulty in assessing CoQ_10_ treatment outcome is that different CoQ_10_ formulations are employed that could differ in the extent or rate of absorption and bioavailability, but such parameters are not always monitored, not even the maximum plasma concentration of CoQ_10_ achieved by the treatments. Finally, it has been suggested that some individuals may have an inherently low capacity to absorb dietary CoQ_10_ for reasons unknown (382).

Despite very limited evidence for treatment effectiveness, taking CoQ_10_ supplement is still being highly recommended for statin drug users, and CoQ_10_ is widely prescribed to mitochondrial disease patients, often as part of a “multivitamin cocktail” (641). Therefore, it is of great clinical significance to elucidate the exact pathophysiological role of secondary CoQ deficiency in these conditions. CoQ_10_ supplementation has also been recommended as adjunctive therapy for various other conditions, such as heart failure, diabetes, and Parkinson’s disease (43). Currently, only mixed and contradictory findings can be found concerning the benefits of CoQ_10_ for these disorders (372, 710–713). Thus, it remains a priority to develop truly effective CoQ_10_ treatments to clarify the potential contribution of partial CoQ deficiency in any disease pathophysiology.

## 8. SUMMARY REMARKS

CoQ is an essential membrane component that every cell synthesizes. Except for the length of the side chain, its exact structure is conserved from bacteria to humans. In other words, the molecule is billions of years old and still plays several vital roles in the life of all, or almost all, cells of the planet. It is indispensable for energy production, and loss of its function in mitochondria is the main factor for why CoQ deficiency can result in severe multisystem human disease. It remains less clear whether, or in which circumstances, the loss of other CoQ functions, such as its antioxidant function, also participates in the pathology associated with CoQ deficiency. In the past two decades, the complex multistep process of CoQ biosynthesis has in large part been worked out in model organisms from bacteria to yeast and to animals. Along with this, patients with primary CoQ deficiency presenting with a wide spectrum of pathologies continue to be identified worldwide. Despite all this, much of the biology of CoQ remains to be understood. We know little or nothing about cellular CoQ metabolism and trafficking, such as how CoQ is delivered from mitochondria to other membranes, how the level of CoQ at the different locations is regulated to match the local needs, what determines its turnover, or how it is broken down or otherwise eliminated. Nor do we understand the paths taken by supplemented exogenous CoQ to reach mitochondria. At the organism level, beyond the link to mitochondrial function, we know little about how much CoQ is required in different cell types and tissues and whether this contributes to the variability and complexity of the clinical presentation of CoQ deficiency. Finally, we know nothing firm about the causes of secondary CoQ deficiencies and the pathophysiological role of such deficiencies in the disorders in which they are observed, including some common age-related diseases.

From a clinical point of view, CoQ deficiency is potentially curable by replacement therapy with exogenous CoQ, of which we know that it can reach tissues and mitochondria. Furthermore, given its two best-known functions, as an indispensable electron transporter in the ETC and as an endogenous membrane antioxidant, CoQ is also a molecule that could have the widest therapeutic applications beyond alleviating deficiencies. However, as one of the most hydrophobic molecules occurring naturally, delivery of CoQ via the oral route has been challenging. Despite the great interest in CoQ’s therapeutic potential, it remains to be seen whether the problem of poor oral bioavailability can ever be sufficiently overcome by better formulations for delivery. On the other hand, providing CoQ supplementation via alternative routes may offer new solutions to the problem. Furthermore, a more profound understanding of all aspects of its synthesis, metabolism, and trafficking could lead to methods to boost intracellular levels of endogenous CoQ. We predict that the future will see many advances on all these fronts. Hopefully, the present review could contribute to raising the awareness and interest of researchers in these questions. It is exciting to look forward to unraveling the biology of this essential component of cellular life and using this knowledge to treat disease or enhance health.

## GLOSSARY

2,4-DHB: 2,4-Dihydroxybenzoic acid
3,4-DHB: 3,4-Dihydroxybenzoic acid
4-AP: 3-Hexaprenyl-4-aminophenol
4-HB: 4-Hydroxybenzoic acid
4-HBz: 4-Hydroxbenzaldehyde
4-HNE: 4-Hydroxynonenal
4-HP: 3-Hexaprenyl-4-hydroxyphenol
4-HPP: 4-Hydroxyphenylpyruvate
4-NB: 4-Nitrobenzoate
8-OHdG: 8-Hydroxy-2-deoxyguanosine
ADCK: aarF domain containing kinase
AFR: Ascorbyl free radical
AIFM2: Apoptosis-inducing factor mitochondria-associated 2
AOX: Alternative quinol oxidase
APG1: Albino or pale green 1
APTX: Aprataxin
ARCR2: Autosomal recessive cerebellar ataxia 2
Asc: Ascorbate
ATP: Adenosine triphosphate
BAT: Brown adipose tissue
BHT: Butylated hydroxytoluene
CHDH: Choline dehydrogenase
CI: Complex I
CII: Complex II
CIII: Complex III
CIV: Complex IV
CV: Complex V
CoQ: Coenzyme Q
CoQ^•−^: Ubisemiquinone
CoQH_2_: Fully reduced form of CoQ
CoQ_i_^•−^: Ubisemiquinone at the Q_i_ site of complex III
CoQ_o_^•−^: Ubisemiquinone at the Q_o_ site of complex III
CsA: Cyclosporine A
Cu^2+^: Cupric copper
CYB5R: NADH-cytochrome *b*_5_ reductase
Cyt: Cytochrome
DAP: Dihydroxyacetone phosphate
DCYTB: Duodenal cytochrome *b*
DDMQ_8_H_2_: 3-Octaprenyl-5-methoxy-1,4-benzoquinone
DHA: Dehydroascorbic acid
DHAR: Dehydroascorbate reductases
DHHB: 3-Hexaprenyl-4,5-dihydroxybenzoic acid
DHODH: Dihydroorotate dehydrogenase
DMAPP: Dimethylallyl pyrophosphate
DMeQ_8_H_2_: 6-Demethyl-CoQ_8_H_2_
DMQ_8_H_2_: 2-Methyl-3-octaprenyl-5-methoxy-1,4-benzoquinone
*E. coli*
: *Escherichia coli*
ENOX: Cell-surface NADH oxidase
ER: Endoplasmic reticulum
ERMES: Endoplasmic reticulum-mitochondria encounter structure
ERP: Electron paramagnetic resonance
ESR: Electron spin resonance
ETC: Electron transport chain
ETFDH: Electron transport flavoprotein dehydrogenase
ETHE1: Encephalopathy protein 1
FAD: Flavin adenine dinucleotide
Fe^3+^: Ferric iron
FET: Forward electron transport
FFA: Free fatty acid
FMN: Flavin mononucleotide
FPP: Farnesyl diphosphate
FPS: Farnesyl pyrophosphate FPP-synthase
FSGS: Focal segmental glomerulosclerosis
FSP1: Ferroptosis suppressor protein 1
G3P: Glycerol 3-phosphate
G3PDH: Glycerol 3-phosphate dehydrogenase
GLUT: Glucose transporter
GPP: Geranyl pyrophosphate
GSH: Glutathione
GSSG: Glutathione disulfide
H_2_O_2_: Hydrogen peroxide
H_2_S: Hydrogen sulfide
HAB: 3-Hexaprenyl-4-aminobenzoic acid
HGA: Homogentisic acid
HHAB: 3-Hexaprenyl-4-amino-5-hydroxybenzoic acid
HHB: 3-Hexaprenyl-4-hydroxybenzoic acid
HMG-CoA: 3-Hydroxy-3-methylglutaryl coenzyme A
HMHB: 3-Hexaprenyl-4-hydroxy-5-methoxybenzoic acid
HRP: Horseradish peroxidase
II_Q_: CoQ-binding site of complex II
IMM: Inner mitochondrial membrane
IMS: Intermembrane space
IPP: Isopentenyl diphosphate
I/R: Ischemia-reperfusion
ISC: Iron-sulfur cluster
IspA: Farnesyl diphosphate synthase
IspB: Octaprenyl diphosphate synthase
KCN: Potassium cyanide
L^•^: Lipid carbon central radical
LDL: Low-density lipoproteins
LO^•^: Lipid alkoxyl radical
LOO^•^: Lipid peroxyl radical
LOOH: Lipid hydroperoxide
MCE: Methylmalonyl-CoA epimerase
MCM: Methylmalonyl-CoA mutase
MDA: Malondialdehyde
MDS: Mitochondrial DNA depletion syndromes
MEF: Mouse embryonic fibroblast
MELAS: Mitochondrial encephalomyopathy, lactic acidosis and strokelike episodes
MEP: 2C-methyl-D-erythritol 4-phosphate
MFN2: Mitofusin2
mGPDH: Mitochondrial glycerol 3-phosphate dehydrogenase
MIOREX: Mitochondrial organization of gene expression
mPTP: Mitochondrial permeability transition pore
MSA: Multiple system atrophy
mtDNA: Mitochondrial DNA
MUT: Methylmalonyl-CoA mutase
NADH: Reduced nicotinamide adenine dinucleotide
NADPH: Reduced nicotinamide adenine dinucleotide phosphate
NH_2_: Amino
NO^•^: Nitric oxide
NOX: NAD(P)H oxidases
NQO1: NAD(P)H:quinone oxidoreductase 1
NS: Nephrotic syndrome
O_2_: Oxygen
O_2_^•−^: Superoxide
ODP: Octaprenyl diphosphate
OE: Overexpression
OH: Hydroxyl
^•^OH: Hydroxyl radical
OHB: 3-Octaprenyl-4-hydroxybenzoate
OMM: Outer mitochondrial membrane
ONOO^•^: Peroxynitrite
OPHP: 3-Octaprenyl-5-hydroxyphenol/2-octaprenyl-6-hydroxyphenol
OPMP: 3-Octaprenyl-5-methoxyphenol/2-octaprenyl-6-methoxyphenol
OPP: Octaprenylphenol
OXPHOS: Oxidative phosphorylation
pABA: *Para*-aminobenzoic acid
PARL: Presenilin-associated rhomboid-like
PC: Phosphatidylcholine
PMET: Trans-plasma membrane electron transport
PMF: Protonmotive force
PMRS: Plasma membrane redox system
PN: Purine nucleotide
POPC: Phospholipid palmitoyl-2-oleoyl-sn-glycero-phosphocholine
PQ: Plastoquinone
PRDX: Peroxiredoxin
PRODH: Proline dehydrogenase
PUFA: Polyunsaturated fatty acid
RET: Reverse electron transport
RISP: Rieske iron-sulfur protein
RNS: Reactive nitrogen species
ROS: Reactive oxygen species
Rot: Rotenone
RQ: Rhodoquinone
*S. cerevisiae*
: *Saccharomyces cerevisiae*
*S. pombe*
: *Schizosaccharomyces pombe*
S1QELs: Suppressors of site I_Q_ electron leak

$S_{2}O_{3}^{2-}$

: Thiosulfate
S3QELs: Suppressors of site III_Qo_ electron leak
SAM: S-adenosylmethionine
SC: Supercomplex
SCAD: Short-chain acyl-CoA dehydrogenase
SCAR9: Autosomal recessive spinocerebellar ataxia
SDO: Sulfur dioxygenase

$SO_{3}^{2-}$

: Sulfite

$SO_{4}^{2-}$

: Sulfate
SOD: Superoxide dismutase
SRD: Short-chain dehydrogenase/reductase
SRNS: Steroid-resistant nephrotic syndrome
SQ_Nf_: Fast-relaxing ubisemiquinone
SQ_Ns_: Slowly relaxing ubisemiquinone
SQOR: Sulfide-quinone oxidoreductase
START: Steroidogenic acute regulatory-related lipid transfer
SUOX: Sulfite oxidase
TST: Thiosulfate sulfurtransferase
VA: Vanillic acid
UCK: Uridine kinase
UCP: Uncoupling protein
UHDBT: 5-Undecyl-6-hydroxy-4,7-dioxobenzothiazol
UMP: Uridine 5-monophosphate
UMPS: Uridine monophosphate synthase
VDAC: Voltage-dependent anion-selective channel
VE: Vitamin E
VE^•^: Vitamin E radical
VEQ: Vitamin E quinone
VLDL: Very low-density lipoprotein
αLnn: Linolenic acid
Δ*p*: Protonmotive force
ΔΨm: Mitochondrial membrane potential

## GRANTS

This work was supported by the Canadian Institutes of Health Research, FDN-159916 (to S. Hekimi).

## DISCLOSURES

No conflicts of interest, financial or otherwise, are declared by the authors.

## AUTHOR CONTRIBUTIONS

S.H. conceived and designed research; Y.W. prepared figures; Y.W. and N.L. drafted manuscript; Y.W., N.L., and S.H. edited and revised manuscript; S.H. approved final version of manuscript.
